# The Clinical Utility of Soluble Serum Biomarkers in Autoimmune Pancreatitis: A Systematic Review

**DOI:** 10.3390/biomedicines10071511

**Published:** 2022-06-26

**Authors:** Ana Dugic, Cristina Verdejo Gil, Claudia Mellenthin, Miroslav Vujasinovic, J.-Matthias Löhr, Steffen Mühldorfer

**Affiliations:** 1 Department of Gastroenterology, Klinikum Bayreuth, Friedrich-Alexander-Universität Erlangen-Nürnberg (FAU), Medizincampus Oberfranken, 95445 Bayreuth, Germany; steffen.muehldorfer@klinikum-bayreuth.de; 2 Faculty of Medicine, Friedrich-Alexander-Universität Erlangen-Nürnberg (FAU), Schloßplatz 4, 91054 Erlangen, Germany; 3 Department of Gastroenterology, Hospital Universitario Fundación de Alcorcón, 28922 Madrid, Spain; cristinaverdej@hotmail.com; 4 Department of Surgery, HFR Fribourg, 1700 Fribourg, Switzerland; claudia.mellenthin@unifr.ch; 5 Department of Upper Abdominal Diseases, Karolinska University Hospital, 14186 Stockholm, Sweden; miroslav.vujasinovic@regionstockholm.se; 6 Department of Medicine, Huddinge, Karolinska Institutet, 14186 Stockholm, Sweden; matthias.lohr@ki.se; 7 Department of Clinical Science, Intervention, and Technology (CLINTEC), Karolinska Institutet, 14186 Stockholm, Sweden

**Keywords:** autoimmune pancreatitis, soluble biomarkers, immunoglobulins, autoantibody, cytokines

## Abstract

Autoimmune pancreatitis (AIP) is a rare etiological type of chronic pancreatitis. The clinical and radiological presentation of AIP often resembles that of pancreatic cancer. Identifying non-invasive markers for their early distinction is of utmost importance to avoid unnecessary surgery or a delay in steroid therapy. Thus, this systematic review was conducted to revisit all current evidence on the clinical utility of different serum biomarkers in diagnosing AIP, distinguishing AIP from pancreatic cancer, and predicting disease course, steroid therapy response, and relapse. A systematic review was performed for articles published up to August 2021 by searching electronic databases such as MEDLINE, Web of Science, and EMBASE. Among 5123 identified records, 92 studies were included in the qualitative synthesis. Apart from immunoglobulin (Ig) G4, which was by far the most studied biomarker, we identified autoantibodies against the following: lactoferrin, carboanhydrase II, plasminogen-binding protein, amylase-α2A, cationic (PRSS1) and anionic (PRSS2) trypsinogens, pancreatic secretory trypsin inhibitor (PSTI/SPINK1), and type IV collagen. The identified novel autoantigens were laminin 511, annexin A11, HSP-10, and prohibitin. Other biomarkers included cytokines, decreased complement levels, circulating immune complexes, *N*-glycan profile changes, aberrant miRNAs expression, decreased IgA and IgM levels, increased IgE levels and/or peripheral eosinophil count, and changes in apolipoprotein isoforms levels. To our knowledge, this is the first systematic review that addresses biomarkers in AIP. Evolving research has recognized numerous biomarkers that could help elucidate the pathophysiological mechanisms of AIP, bringing us closer to AIP diagnosis and its preoperative distinction from pancreatic cancer.

## 1. Introduction

Autoimmune pancreatitis (AIP) is a rare etiological subtype of chronic pancreatitis [1] first introduced by Yoshida et al. in 1995 as a diagnostic entity; it is described as a pancreatic disease that involves autoimmune mechanisms that are responsive to steroid treatment [2]. In 2001, Hamano et al. identified immunoglobulin G4 (IgG4) as a serological feature of AIP [3]. Several diagnostic criteria, some of which include serology as a cardinal component, have been established since then. The most recent International Consensus Diagnostic Criteria (ICDC) appreciate IgG4 as the most preferred serologic test for AIP diagnosis [4]. Presently, AIP has two types with distinctive clinical presentation, histology, and natural course.

Type 1 AIP is considered a pancreatic manifestation of a multi-organ IgG4-related disease (IgG4-RD), characterized by abundant tissue infiltration with IgG4+ plasma cells [5]. With typical onset in the seventh decade and male predominance of approximately 3:1, the most common clinical presentation is painless obstructive icterus (60–75%). Radiographically, focal or diffuse pancreatic enlargement is observed, accompanied by segmental or diffuse pancreatic ductal narrowing, which altogether make the preoperative distinction from pancreatic adenocarcinoma (PDAC) a major clinical challenge. This AIP type, as a part of IgG4-RD, is often accompanied by other organ involvement (OOI), such as IgG4-associated cholangitis (IAC), parotid/lacrimal gland involvement, retroperitoneal fibrosis, mediastinal lymphadenopathy, and interstitial pneumonia. OOI can be proven either histologically or by resolution after steroid treatment. Histologically known as lymphoplasmacytic sclerosing pancreatitis (LPSP)*,* type 1 AIP exhibits a typical pattern of periductal lymphoplasmacytic infiltration, storiform fibrosis, obliterative phlebitis, and - abundant IgG4+ plasma cell tissue infiltration (≥10 cells per high power field) [4]. Additionally, patients with type 1 AIP commonly display high levels of serum IgG4 and an excellent steroid therapy response. Interestingly, recent literature data report cases of “IgG4-unrelated type 1 AIP” (cases with the fulfillment of histologic criteria for type 1 AIP without serum IgG4 elevation) [6,7].

Type 2 AIP known as idiopathic duct centric pancreatitis (IDCP) with a granulocytic epithelial lesion (GEL), affects the younger age population and is sometimes (up to 25%) accompanied by inflammatory bowel disease. The main histological features of type 2 AIP comprise the neutrophilic epithelial lining infiltration of small and medium-size pancreatic ducts and the absence of or scant (<10 cells/HPF) IgG4+ plasma cells. Serum IgG4 elevation rarely occurs, whereas clinical presentation includes equally obstructive jaundice and pancreatitis. Type 2 AIP is thought to be more common in Europe and Western countries, without gender preponderance and with a low relapse rate [8].

The clinical and radiological presentation of both AIP types often resembles that of pancreatic cancer. Presently, a definite distinction is possible only by tissue specimen analysis obtained by surgery or other invasive procedures, which harbor a considerable risk of complications. Recent data suggested the presence of benign conditions in 8.4% of cases that underwent surgery due to suspicion of pancreatic cancer; of those, AIP accounted for up to one-third [9]. Hence, investigating non-invasive, preoperative methods for early AIP detection and its differentiation from pancreatic cancer is of utmost importance to avoid unnecessary surgery or a delay in steroid therapy. Particularly, markers other than IgG4 deserve close attention, especially in the context of the seronegative type 2 AIP (broadly prevalent in Europe) and the moderate IgG4 sensitivity in establishing type 1 AIP diagnosis.

A substantial number of original studies and narrative reviews can be found on different AIP biomarkers; however, to our best knowledge, this is the first systematic review that encompasses and integrates all relevant data on this topic.

### Aim

This study aims to systematize and revisit all current evidence on the clinical utility of different serum biomarkers in AIP diagnosis, distinguishing AIP from PDAC, and predicting disease course, steroid therapy response, and relapse.

## 2. Methods

### 2.1. Search Strategy

The literature review was performed for articles published up to 1 August 2021, by searching the electronic databases of MEDLINE, Web of Science, and EMBASE. The search was restricted to studies conducted on human subjects in English, German, and Spanish. The following search terms were used: “immunoglobulin G4–Related Disease”, “autoimmune pancreatitis”, “lymphoplasmacytic sclerosing pancreatitis”, “idiopathic duct centric pancreatitis”, “immunoglobulin G4”, “IgG4”, “anti-lactoferrin”, “anti-carbonic anhydrase-I”, “anti-carbonic anhydrase–II”, “SPINK”, “ubiquitin”, “trypsinogens”, “*N*-glycan”, “IgG/IgG4 ratio”, “eosinophilic”, “anti-plasminogen binding peptide”, “rheumatoid factor”, “antinuclear antibodies”, “antineutrophil cytoplasmic antibodies”, “amylase–alpha 2A”, “plasminogen binding protein”, and “miRNA”. After retrieving eligible records, the “snowball strategy” was employed by manually searching the references of relevant articles.

### 2.2. Inclusion/Exclusion Criteria

Studies on IgG4 were included subject to the following criteria: (1) related to autoimmune pancreatitis; (2) provided detailed diagnostic AIP criteria; and (3) provided the cutoff value for serum IgG4 and mean/median values of IgG4 if available, in tested sera. Studies that assessed markers other than IgG4 did not necessitate marker quantification to be included, but had to be related to AIP with clearly defined diagnostic criteria.

Studies that did not exclusively relate to AIP, but the IgG4-RD spectrum in general, were considered eligible only if the outcomes of patients with AIP could be extrapolated from the available data.

Exclusion criteria were as follows: (1) review articles, case reports, and editorials; (2) studies on IgG4 in which the extrapolation of patients with AIP data was not possible; (3) studies on IgG4 with no IgG4 quantification; (4) case series with less than 10 patients with AIP or <10 controls; and (5) studies that included non-soluble markers (immunohistochemistry, genes, and cell markers).

### 2.3. Data Extraction

Two independent investigators (AD and CVG) screened and reviewed articles to determine eligibility. All disagreements were resolved by consultations with senior authors (SM and JML). The following information was collected and extracted into excel datasheets: author, year of publication, country, candidate biomarker, detection method, study cohort, diagnostic criteria for AIP, marker cutoff value, marker prevalence, mean/median values across groups, sensitivity, and specificity. Differences in biomarker levels depending on OOI, steroid treatment response, and relapse were also assessed. The proportion of different AIP subtypes has been noted when available.

### 2.4. Reporting

The reporting followed the Preferred Reporting Items for Systematic Reviews and Meta-Analyses (PRISMA) guidelines [10].

## 3. Results

**Figure 1:**
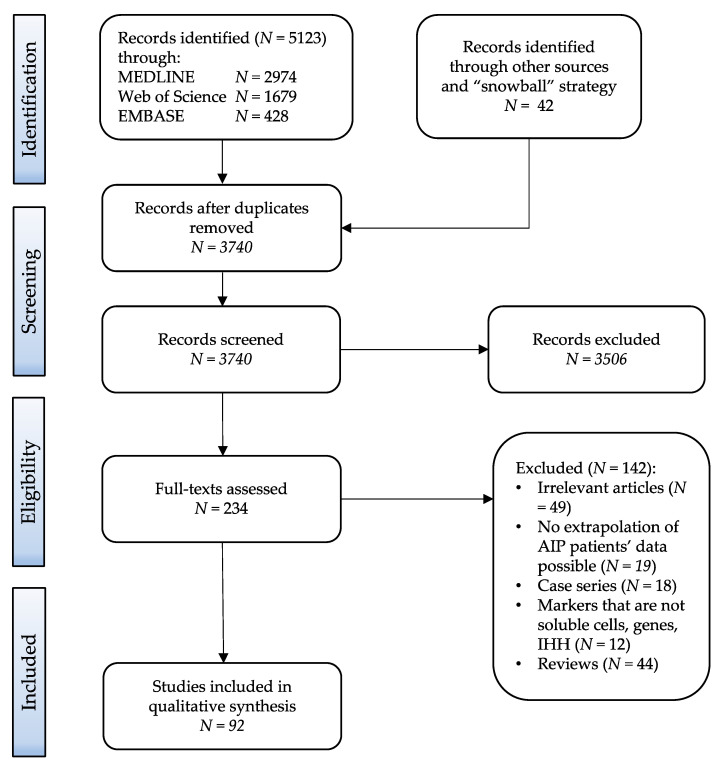
PRISMA flow diagram of the study selection process. After the search of databases, 3740 publications were screened, and 92 studies met the inclusion criteria. AIP—autoimmune pancreatitis; IHH—immunohistochemistry.

Among the 5123 identified records, 234 full-text articles were assessed for eligibility and 92 studies were included in the qualitative synthesis [3,6,7,11,12,13,14,15,16,17,18,19,20,21,22,23,24,25,26,27,28,29,30,31,32,33,34,35,36,37,38,39,40,41,42,43,44,45,46,47,48,49,50,51,52,53,54,55,56,57,58,59,60,61,62,63,64,65,66,67,68,69,70,71,72,73,74,75,76,77,78,79,80,81,82,83,84,85,86,87,88,89,90,91,92,93,94,95,96,97,98,99]. A PRISMA flow diagram with reasons for study exclusions is presented in Figure 1. Most of the studies originated from Asia (*n* = 64), followed by Europe (*n* = 20), the United States of America (USA) (*n* = 7), and Australia (*n* = 1).

Apart from IgG4, which was by far the most studied biomarker, we identified autoantibodies against the following: lactoferrin, carboanhydrase II, carboanhydrase IV, plasminogen binding protein (PBP), amylase-α2A, cationic (PRSS1) and anionic (PRSS2) trypsinogens, pancreatic secretory trypsin inhibitor (PSTI/SPINK1), type IV collagen, and type VII collagen. The identified novel autoantigens included laminin 511 (both full-length FL and truncated-form E8), annexin A11, HSP-10, and prohibitin. Other candidate serum biomarkers included cytokines, decreased complement levels, circulating immune complexes, *N*-glycan profile changes, aberrant miRNAs expression, decreased IgA and IgM levels, increased IgE levels and/or peripheral eosinophil count, and changes in apolipoprotein isoform levels.

## 4. Discussion

Over the last decades, remarkable progress has been made in understanding AIP immunopathogenesis. Consequently, novel biomarkers have continuously emerged. In this frontier article, all current evidence on soluble biomarkers in AIP published to date are summarized.

### 4.1. IgG4 Properties and Their Role in AIP Pathogenesis

**Figure 2:**
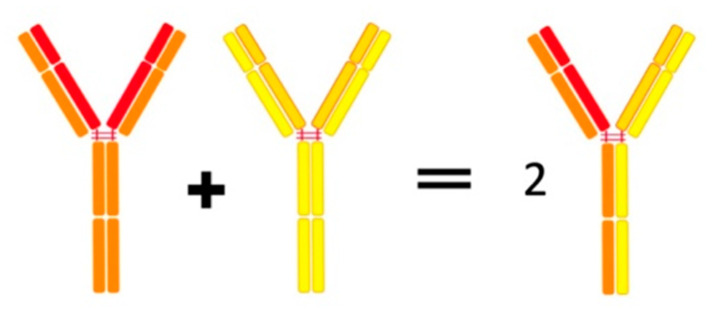
Configuration of IgG4 molecule after Fab exchange.

IgG1 antibodies seem to be the main culprit for pancreatic tissue injury in AIP; however, the exact role of IgG4 in inflammation remains unclear. The IgG4 antibodies display several unique properties suggesting anti-inflammatory and tolerance-inducing activity [100]. IgG4 exchange half molecules that transform into asymmetrical, bispecific antibodies with low affinity for Fcγ receptors and target antigens in a dynamic process called “Fab-arm exchange” presented in Figure 2 [101]. Another interesting aspect is that IgG4 can interact with other immunoglobulins via Fc-mediated aggregation, resembling rheumatoid factor activity [52]. Finally, IgG4 antibodies are incapable of activating the classical complement cascade because they cannot bind the Cq1 complement component [102]. However, IgG4 antibodies are shown as tissue destructive in some diseases, as observed in pemphigus vulgaris [103] and idiopathic membranous nephropathy [100]. The landmark study by Shiokawa et al. [104] demonstrated the pathogenic effects of IgG1 and IgG4 on neonatal mouse pancreas by passively transferring IgG purified from the sera of patients with IgG4-RD. Interestingly, IgG4 induced pancreatic injury when transferred alone, whereas IgG1 and IgG4 co-transfer resulted in reduced tissue damage caused by IgG1. Collectively, the role of IgG4 as a protective, proinflammatory that mediates AIP occurrence, or an epiphenomenon that reflects counter-regulatory response failure in which IgG4 concentrations progressively rise in an attempt to diminish the primary immune response, is unclear.

### 4.2. IgG4 as Diagnostic and Disease Severity Marker

The initial report by Hamano et al. [3] revealed a high prevalence of elevated IgG4 in patients with AIP and its rare presence in other diseases, indicating IgG4 as a promising marker for AIP diagnosis. Thereafter, numerous studies have validated its diagnostic accuracy, reporting variable sensitivity (53% [84], 76% [30], 87% [64], and 91% [76]) and specificity over 90% [19,76,84,87]. The reported differences are mostly explained by demographic heterogenicity with a significantly higher incidence of seropositive type 1 AIP in Asia, and a preponderance of seronegative type 2 AIP in Western countries [8]. Generally, serum IgG4 of >135 mg/dL has been widely accepted as a cutoff value for AIP diagnosis. IgG4 levels of >2× the upper limit of normal (ULN) are considered highly accurate in distinguishing AIP from other diseases, especially PDAC. Supplemental Table S1 includes studies on the diagnostic accuracy of IgG4 in AIP diagnosis.

Clinical AIP presentation has also been reported to differ based on serum IgG4 concentrations. Compared to patients with normal IgG4 levels, those with increased IgG4 are regarded as highly active, with a higher jaundice incidence at onset and worse radiological features [59,105]. There are a number of extrapancreatic manifestations correlated with IgG4 level [39]. Additionally, different organs seem to be involved based on the extent of IgG4 elevation [44,61]. The study by Ishikawa et al. [42] revealed that all patients with AIP with kidney involvement displayed elevated IgG4, of whom 81% (*n* = 17) had IgG4 higher than twice the ULN. Retroperitoneal fibrosis and lacrimal and salivary gland involvement are also accompanied by high IgG4 levels in patients with AIP [32,61,106]. Studies that investigate the relationship between IgG4 and the extension of extrapancreatic lesions in AIP are presented in Supplemental Table S2.

Special consideration should be given to AIP with bile duct involvement, known as IgG–4-associated cholangitis (IAC), which often mimics bile duct malignancy or primary sclerosing cholangitis (PSC). Great effort has been made to identify a reliable discriminatory non-invasive marker, because treatment and prognosis substantially differ between IAC and PSC [33,67,70,96]. Typically, IgG4 is widely used to distinguish IAC from PSC because normal IgG4 levels are most often found in PSC. A recent study from Sweden identified IgG1 and IgG2 as additional biomarkers in differentiating between PSC and IAC [96]. High IgG2 or IgG4 levels were proposed to identify patients with AIP, whereas high IgG1 in those with low or normal IgG2 and IgG4 identified patients with PSC. The relevant studies on this topic are presented in Supplemental Table S3.

### 4.3. Serological Distinction between Type 1 and Type 2 AIP

Two AIP types have been identified based on IgG4 level and histological features. Type 1 AIP is traditionally accompanied by IgG4 elevation (seropositive), whereas type 2 AIP is a seronegative form. Interestingly, not all patients with type 1 AIP have elevated IgG4 levels. Several studies have described a seronegative case of type 1 AIP defined as LPSP with typical IgG4+ plasma cell abundance on histology and IgG4 levels below the cutoff value (IgG4 of <135 mg/dL) [7]. Similar to patients with seropositive type 1 AIP, patients with seronegative type 1 AIP tend to be older than 50 years, with OOI and a high relapse rate. This is in contrast with patients with type 2 AIP who are usually younger by a decade, with no OOI and an absence of relapse after steroid treatment [6,50]. Several authors have attempted to define additional laboratory markers for distinguishing between AIP subtypes (Supplemental Table S4). Peripheral eosinophilia and elevated serum IgE were one of the proposed markers, but with inconsistent results [6,50]. Detlefsen et al. [21] reported significant c–ANCA elevations in some patients with type 2 AIP, whereas a group from Spain [79] proposed anti-alpha 2 amylases as a candidate marker. Nevertheless, to date, IgG4 remains the most utilized serum marker for AIP subtype distinction.

### 4.4. Serological Distinction between AIP and Pancreatic Cancer

Patients with seronegative type 1 and 2 AIP are more frequently subjected to surgery than those with seropositive type 1 AIP, especially if they exhibit focal pancreatic mass on imaging [6,7]. Interestingly, the co-occurrence of pancreatic tumors (benign and malignant) has been reported in up to 7% of patients with AIP [5,107]. Patients with AIP are at increased risk for malignant diseases compared to the general population; however, the relationship between pancreatic cancer and AIP remains a subject of ongoing research [108]. A recent review reported more frequent PDAC occurrence in type 1 AIP, which is typically metachronous in character and generally found in the part of the pancreas that is affected by AIP [109].

The main clinical concern is to safely diagnose AIP and avoid the misdiagnosis of cancer as AIP. The pancreas is not easily accessible for histological examination; however, a 2-week steroid trial is a widely accepted alternative non-invasive approach [110]. Among serum markers, IgG4 and carbonic anhydrase (CA) 19–9 have the highest diagnostic value (Supplemental Table S5). The two-fold elevation of IgG4 (>280 mg/dl) has been shown as most useful, with sensitivity ranging from 53% [30] to 77% [64] and high specificity (98% [64], 99% [30]); hence, it is incorporated as a Level 1 serology in ICDC. However, mild (<2-fold) serum IgG4 elevation is seen in up to 10% of subjects with pancreatic cancer, with 1–2.4% having more than two-fold IgG4 elevation [16,68]. As opposed to AIP, increased CA19-9 levels are typically observed in pancreatic cancer, with a sensitivity of 79–95% and specificity of 82–91% [111]. Interestingly, CA19-9 elevation is commonly encountered in patients with AIP, with up to 38% having values over >100 U/mL [94]. Collectively, measurements of either CA19-9 or IgG4 level alone are not accurate enough to distinguish AIP from cancer. Accordingly, investigators from the USA demonstrated the best diagnostic accuracy (86%) by combining IgG4 over 280 mg/dL and CA19-9 below 85 U/mL [30]. Similarly, a combination of IgG4 of >1.0 g/dL and CA19-9 of <74 U/mL yielded a sensitivity of 94% and specificity of 100%, as shown by a group from the Netherlands [94]. Among markers other than IgG4, serum eosinophilia and raised total serum IgE levels in AIP were suggested as potentially useful for discrimination from pancreatic cancer. However, a small sample [95] and the inclusion of only patients with type 1 AIP [98] are limitations that warrant further verification in larger series. In addition to single markers, biomarker panels based on broad proteomic analysis appear promising to discriminate AIP from PDAC [24,25].

Serum micro RNAs (miRNAs) have been reported to show high accuracy in differentiating pancreatic cancer from AIP (Supplemental Table S6). Akamatsu et al. [11] have identified four specific miRNAs (miR-7, miR-34a, miR-181d, and miR-193b) that are associated with constitutive mitogen-activated protein kinase activation, commonly seen in pancreatic neoplastic diseases (PDAC and IPMN). Thus, it is hypothesized that these miRNA elevations can differentiate between PDAC and AIP, with overexpression in PDAC and absence/normality in AIP. Another study from Japan [31] identified the significant upregulation of miR-150-5p in AIP compared to CP, pancreatic cancer and healthy controls. Collectively, circulating miRNAs seem to be promising novel biomarkers and therapeutic targets in AIP. However, the significance of their differential expression in AIP needs further validation.

### 4.5. Role of Biomarkers in Therapy Monitoring and Relapse Prediction

Corticosteroid therapy has become standard for inducting AIP remission [112,113]. Unfortunately, high relapse rates following remission have been commonly encountered in everyday practice. The occurrence of relapse is relatively high, ranging from 38% [81] to 53% in Europe and the USA, where steroids are usually withdrawn after remission due to potential adverse effects [30]. Data from Asian countries implicate that the use of low-dose maintenance steroid therapy may provide beneficial outcomes [36]. However, substantial inconsistencies were found regarding optimal therapy and follow-up after remission. Another clinical concern is the absence of reliable relapse predictors. Diffuse pancreatic enlargement [77] and proximal bile duct involvement [56] are considered clinical relapse predictors; however, a reliable laboratory marker that would enable objective risk interpretation remains unavailable. Some studies report a positive association between relapse and increased IgG4 level at diagnosis (>upper limit of normal (ULN), >2× ULN) [41], whereas others fail to observe any association [7,114]. Most studies recognize a decreased IgG4 shortly after initiating CST; however, IgG4 level normalization has been achieved in less than two-thirds [35,56]. Accordingly, continuously high IgG4 titer after steroid treatment [56,82] has been proposed to be linked with a higher relapse rate. Low decrease or re-raise of IgG4 after remission are also recognized as potential relapse predictors [115,116]. Nevertheless, due to the large heterogeneity of studied cohorts and different therapy regimens, the confidence of these predictors remains questionable.

A recently published study from Japan proposed autotaxin (ATX) as a promising marker for therapy monitoring and relapse prediction. [28]. ATX is a secreted enzyme that is essential for lysophosphatidic acid production, with some evidence suggesting a role in tissue remodeling and fibrosis. [117] Fukiage et al. assessed 24 male patients with type 1 AIP who displayed significantly decreased serum ATX levels after CST induction and maintenance, compared to ATX levels before treatment. Additionally, increased ATX level from induction to maintenance therapy was associated with relapse. However, these findings were based on a small sample of male subjects and need to be verified in larger series. Studies that include candidate biomarkers for therapy monitoring and prediction of relapse are presented in Supplemental Table S7.

### 4.6. Autoantibodies

Diverse autoantibodies have been described in the sera of patients with AIP, corroborating the hypothesis that AIP is an immune-mediated disease (Supplemental Table S8). Studies have implicated the role of microbial antigens as initiating agents in AIP pathogenesis, with H. pylori and Hepatitis E virus [118] as the most investigated. An emerging body of evidence indicates not only pathogenic but also commensal microorganisms may trigger AIP through molecular mimicry mechanisms [99]. Moreover, environmental factors, such as chronic exposure to industry solvents and oils in “blue-collar” workers might be associated with autoimmunity induction in AIP [119]. The following section presents an overview of the autoantibodies that might be involved in AIP pathogenesis.

#### 4.6.1. Antibody against Carboanhydrase II (anti-CA II)

Early evidence of an association between H. pylori infection and AIP came from in silico protein analysis by Guarneri et al. [120], who found homology in amino acids between H. pylori carbonic anhydrase and human CA II). He proposed *H. pylori* infection as a trigger for autoantibody activation against the enzyme of the ductal pancreatic epithelium (CA II) in genetically predisposed subjects. Thereafter, the clinical significance of anti-CA II was assessed by several studies worldwide. Okazaki et al. [71] demonstrated high serum levels of anti-CA II in 59% of AIP (*n* = 17) but none in the alcoholic patients with CP (*n* = 17). The prevalence of anti-CA II among different AIP series was also relatively high and varied between 66% (*n* = 13) [23], 83% (*n* = 12) [80], and 89% (*n* = 9) [38]. Aparisi et al. [13] showed a parallel anti-CAII and IgG4 increase in patients with AIP. Contrastingly, a study from Poland showed the low sensitivity and specificity of anti-CA II (45.3% and 74.3%, respectively, at a cutoff of 38.4 ng/ml), which was increased not only in patients with CP but also in patients with pancreatic cancer. This is in line with Detlefsen et al. [21] who found no significant differences in mean serum anti-CA II concentrations between AIP (*n* = 29), pancreatic cancer (*n* = 17), and alcoholic CP (*n* = 41). Altogether, the significance of anti-CA II antibodies in differentiating AIP from other diseases remains equivocal. Carboanhydrase IV (CA IV), another CA isoenzyme, was proposed as a potential target antigen in AIP, with AIP patients having a higher prevalence of elevated anti-CA IV compared to normal controls [69]. However, a small study cohort warrants further validation of this biomarker.

#### 4.6.2. Antibody against PBP

Another example of *H. pylori* antigen mimicry was proposed by Frulloni et al. [26], who identified amino-acid sequence homology between the PBP of *H. pylori* and human protein ubiquitin-protein ligase E3 component n-recognin 2 (UBR2) expressed in pancreatic acinar cells. IgG antibodies against PBP peptides were found in 94% of AIP sera and in only 5% of PDAC sera, suggesting the PBP antibody is likely a useful diagnostic tool to discriminate AIP from PDAC (94% sensitivity and 95% specificity). In the same study, bacterial involvement was further corroborated by a high prevalence of *H. pylori* seropositivity in patients with AIP (81–83%) compared to other groups (40–50% seropositivity). Contrastingly, the subsequent studies from the Netherlands [15], Denmark [21], UK [121], and Germany [24] could not confirm the diagnostic utility of PBP antibodies in diagnosing AIP. No difference was found in H. pylori seropositivity between patients with AIP and those with other pancreatic diseases [24]. No correlation was determined between PBP status and H. pylori seropositivity in healthy subjects [15]. Additionally, Jesnowski et al. [122] could not identify H. pylori nucleic acids or proteins in tissue samples from individuals with AIP. This was in line with the results by Culver et al. [121], who also found no evidence to support the association between H. pylori infection and AIP.

#### 4.6.3. Antibody against Lactoferrin (anti-LF)

Lactoferrin (LF) is an iron-binding glycoprotein, which is a major mediator in immune defense, pathogenic response, and non-pathogenic injury [123]. In the pancreas, LF is present in zymogen granules of acinar cells. An early report by Okazaki et al. [71] demonstrated a high prevalence of serum anti-LF in AIP versus chronic pancreatitis and normal subjects. However, two recent studies [21,80] could not confirm the diagnostic utility of anti-LF due to its low specificity for AIP.

#### 4.6.4. Antibody against Alpha 2A Amylase (Anti-Amylase α-2A) and HSP–10

CA II and LF are present in the normal pancreas and also in other organs, including the lactating breast, bile ducts, distal renal tubules, and some exocrine glands; amylase α is exclusively found in pancreatic tissue (organ-specific antigen) [80]. Several laboratories have reported a higher prevalence of anti-amylase α-2A than that of IgG4, ranging between 76% [79] and 100% [23] in patients with AIP. The sensitivity and specificity in diagnosing AIP were shown as 76% and 78% by the Spanish group, respectively, which was lower than that shown in a study from Japan (88% and 99%, respectively). The combination of anti-amylase α-2A with other markers (IgG4 and/or anti-CA II) lowered its sensitivity, but the specificity increased up to 99% [79,80]. Anti-amylase α-2A positivity in PDAC was significantly lower than in AIP; thus, this marker combination might likely be useful in differentiating AIP cases from PDAC. The potential role of anti-amylase α-2A in differentiating AIP subtypes was also speculated, but the results were inconclusive due to the small sample size [79].

Surprisingly, a high prevalence (88%) of anti-amylase α-2A was detected in patients with fulminant type 1b diabetes mellitus, which is a form of diabetes with an absence of autoantibodies typical for type 1 diabetes mellitus (T1DM) [23]. Takizawa et al. [90] identified antibodies to heat shock protein 10 (HSP–10) in 92% of AIP and 81% of patients with T1DM, whereas only 8% of patients with chronic alcoholic pancreatitis and 1.4% of healthy controls exhibited these antibodies. However, the significance of this finding is not entirely clear. Hence, larger studies are needed to evaluate the pancreatic specificity of amylase α-2A because the involvement of this antigen is of interest in the pathogenesis of both AIP and T1DM.

#### 4.6.5. Antibodies against Cationic (PRSS1) and Anionic (PRSS2) Trypsinogens and Pancreatic Secretory Trypsin Inhibitor (PSTI/SPINK1) Antibodies

Antibodies against PSTI and trypsinogen are proposed as markers of acinar cell damage in AIP [14,25,58]. Löhr et al. demonstrated the loss of acinar cells accompanied by elevated IgG antibody titers against PRSS1, PRSS2, and PSTI in patients with AIP. The serum autoantibody data analysis showed an accuracy of 80% (sensitivity 68%, specificity 90%) in distinguishing AIP from non-AIP CP subjects. The serum trypsinogen concentration remained unaffected by pancreatic tissue damage, and the ratio of PRSS1 to PRSS2 was 1:2 in patients with AIP. This was notably different from patients with non-AIP CP, in whom the cationic to anionic trypsinogen ratio was inversed [58]. However, the significance of this phenomenon has not yet been elucidated. Asada et al. [14] reported elevated anti-PSTI antibodies of IgG1 type in 30–40% of patients with AIP. Interestingly, no correlation was found between IgG4 and anti-PSTI antibody levels, which is in accord with Löhr, who found no differences in the antibody level between types 1 and 2 AIP. Both studies demonstrated inadequate PSTI and trypsinogen antibodies in the control groups, implying that these antibodies might be typical for AIP.

#### 4.6.6. Novel Candidate Antigens

In recent years, several autoantigens have been identified as novel targets in IgG4-RD. These include galectin 3, prohibitin, annexin A11, and laminin 511. The latter two will be described in more detail, as they are particularly involved in AIP pathogenesis.

Annexin A11, the calcium-dependent phospholipid-binding protein, is proposed as a novel target autoantigen. Hubers et al. [102] detected annexin A11-specific IgG4 and IgG1 antibodies in the serum of multiple patients with AIP/AIC (*n* = 50) and not in those with PSC (*n* = 20) and pancreatobiliary malignancies (*n* = 27). Cell injury is proposed to give rise to intracellular annexin exposure, which in turn activates IgG1 and IgG4 responses. Its predominant expression in ductal cells corresponded to the distribution of tissue damage observed in histological specimens in the pancreas. As shown by Hubers et al., IgG4 competitively blocks the binding of IgG1 for shared annexin A11 epitopes. This observation corroborates assumptions that increased IgG4 levels might represent a regulatory phenomenon that aims to attenuate proinflammatory IgG1 activity.

Laminin 511 is a heterotrimer that belongs to extracellular matrix (ECM) proteins and mediates cell–ECM adhesion. Its truncated form, laminin 511–E8, was identified as a target autoantigen in a recent study by Shiokawa et al. [83]. IgG/IgG1 antibodies against laminin 511–E8 were detected using an enzyme-linked immunosorbent assay in 51% (26/51) of patients with AIP compared to only 1.6% (2/122) in controls. After immunization with human laminin 511–E8, AIP-like histological changes were found in murine pancreata. This indicated that IgG and IgG1 bind to laminin 511–E8 in the pancreas. IgG4 response has also been documented, although with non-specific IgG4 antibodies that were not directed toward laminin. Presently, their role is not completely clear.

Prohibitin was identified by Du et al. [22] as a potential target antigen in IgG4-RD. Prohibitin antibodies were found in 73.5% (*n* = 34) of patients with definite AIP, 53.3% (*n* = 15) with Mikulicz’s disease, 54.5% (*n* =11) with retroperitoneal fibrosis, and 89.7% (*n* = 29) with other probable IgG4-RD but in only 1.4% (*n* = 70) of healthy donors. Galectin–3 is another promising antigen described in the context of IgG4-RD [124]. Serum anti-galectin-3 antibodies of predominantly IgG4 isotype were identified in patients with IgG4-RD, whereas the response to rituximab was associated with decreased antibody levels. Again, it is not clear whether these antibodies have a causative role in disease pathogenesis, or if they represent a secondary phenomenon.

#### 4.6.7. Other Antibodies

Other non-specific markers of autoimmunity, such as antinuclear antibodies and rheumatoid factors, show variable prevalence in the sera of patients with AIP [12,51,59,75,84,88,97]. Initially thought to play a diagnostic role in AIP, these markers are not useful due to low sensitivity.

Serum IgM and IgA antibodies have been found to be decreased in patients with untreated AIP [89]. Increased ratios of IgG/IgM and IgG/IgA in patients with AIP have been shown to be useful in differentiating AIP from other diseases, including PDAC and chronic pancreatitis. Interestingly, these ratios are also shown to simultaneously decrease with improved clinical symptoms after CST initiation.

The study by Hao et al. demonstrated an increased prevalence of an IgG4 antibody subtype, hybrid κ/λ antibody, in patients with AIP. This asymmetric molecule consists of two IgG4 heavy chains plus one ĸ and one ƛ light chain due to “Fab-arm exchange.” In combination with the IgG4 antibody, hybrid κ/λ increased the sensitivity of IgG4 in diagnosing AIP and differentiating it from pancreatic cancer, without compromising specificity [125].

An antibody against anti-collagen IV antibodies (ACIV-Ab) has been identified by Liu et al. [57], who found high collagen IV and ACIV-Ab expression in the pancreatic tissue of patients with AIP. Additionally, increased serum ACIV-Ab levels were also noted. However, it is not clear whether these antibodies contribute to inflammation, or whether they might just be a response to newly exposed collagen IV antigens in inflammation.

### 4.7. Miscellaneous Markers

#### 4.7.1. Changes in Serum *N*-Glycan Profile

Many proteins are modified by *N*-glycosylation, which refers to the attachment of *N*-acetylglucosamine to the nitrogen atom of an Asn side chain. *O*-Glycosylation occurs on amino acids with functional hydroxyl groups (most often Ser and Thr) [126]. Changes in the glycosylation pattern of serum IgG represent both predisposition and pathophysiological disease mechanisms and are recognized as potential biomarkers of activity in chronic inflammatory diseases [127]. The agalactosylation of G-bound glycan fraction is proposed to correlate with decreased galactosyltransferase activity in T and B cells. Agalactosylated G-bound glycan fraction upregulation was documented in patients with AIP in a study by Tomoda et al. [93]. Especially, glycans #3410, #3510, and #4510 were increased in patients with AIP regardless of their IgG4 level, which implies their potential role in diagnosing seronegative AIP forms. Moreover, they might help distinguish AIP from PDAC in IgG4-positive patients, as they were not elevated in patients with pancreatic cancer, chronic pancreatitis, and IPMN patents. No correlation was observed between *N*-glycan expression and clinical symptoms, OOI, steroid treatment, and IgG4 level.

#### 4.7.2. Complement

As previously discussed, IgG4 cannot bind the C1q complement component, which in turn blocks classical complement cascade activation. However, Muraki et al. [63] found decreased serum complement levels (C3, C4, and CH 50) and increased concentrations of circulating immune complexes (CIC) in AIP. In parallel, increased IgG4 levels and IgG4 subclass of CIC in AIP were also observed. While mannose-binding lectin and alternative pathways were not shown to contribute to the AIP pathogenesis, the IgG1-mediated activation of classical complement cascades may play an important role.

#### 4.7.3. Serum Apolipoprotein Isoforms

Apolipoprotein A2 (apoA2) is a major component of high-density lipoproteins (HDL) which stabilizes HDL particles. Reduced serum levels of apoA2 isoforms, especially apoA2-ATQ/AT and reduced specific apoA2 isoform hypo-processing patterns, have been proposed as useful in differentiating disease activity in AIP and could aid the differentiation between AIP and pancreatic cancer [53]. Interestingly, this reduction in apoA2-ATQ/AT heterodimer might not be accompanied by decreased total apo-A2 level. Contrarily, an increased serum level of total apo-A1 and apo-A2 has been documented in AIP versus pancreatic cancer [24].

### 4.8. T Helper Lymphocyte Response in AIP and the Role of Cytokines as Biomarkers

Both self-antigens and exogenous antigens (microbes, industrial solvents, and allergens) have been reported to trigger aberrant immune activity in AIP. Okazaki et al. [71] were one of the first to propose the predominance of T helper type 1 (Th1) over T helper type 2 (Th2) response in AIP pathogenesis by demonstrating an increased interferon γ in the peripheral blood of AIP patients, and no difference in IL-4, compared to controls. Nowadays, Th2 cytokines (IL-4 and IL-13) and T regulatory cell (Treg) cytokines (IL-10) are considered major promoters of excessive IgG4 production. Additionally, Tregs are responsible for fibrosis by producing transforming growth factor β. Meanwhile, Tregs maintain continued immune tolerance and prevent immunological reactions by releasing IL-35. Ito et al. [43] identified Treg anti-inflammatory activity through elevated plasma IL-35 and tissue IL-35 subunit expression in patients with type 1 AIP. Supplemental Table S6 includes studies that investigate cytokines in AIP.

Common atopic history (15% (12/78) [78], 36% (24/67) [55], and 44% (20/45) [45]), peripheral blood eosinophilia (12% (9/78) [78], 16% (10/62) [55], 43% (6/14) [128], and 52% (13/25) [98]), and raised IgE titers (34% (12/35) [45], 60% (32/53) [55], and 86% (36/42) [34]) further corroborate Th2 involvement in patients with AIP (Supplemental Table S9). T cells may also contribute to AIP/IgG4-RD pathogenesis through dysregulated follicular T helper cell (Tfh) activity. These cells are thought to induce the class switching of IgG4 by secreting IL-21 in correlation with IL-4 and IL-10 [129].

**Figure 3:**
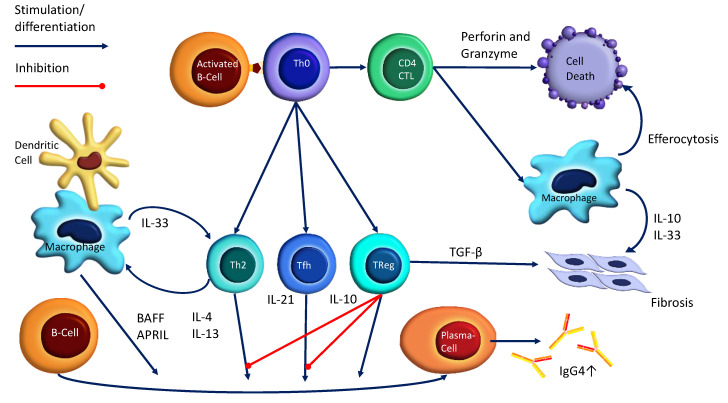
Schematic model of the major pathophysiologic pathways in AIP/IgG4-RD. Unknown antigens seem to be initiating events in the pathogenesis of AIP. Following the uptake and processing of an antigen by an activated B cell, peptide is presented to the CD4+ Th cell subpopulation in the context of MHC–II molecules. This, in turn, leads to the activation of different cytokine signaling pathways that ultimately determine their effector function. Th2 response and regulatory Th cells (Tregs) play a key role in the excessive production of IgG4 and tissue fibrosis. Additionally, IL-21, secreted by dysregulated follicular Th cells, is thought to induce the class switching of IgG4 [129]. Cytotoxic T lymphocytes (CD4^+^CTLs) induce apoptosis and activate macrophages. Activated macrophages clear apoptotic cells by efferocytosis and profibrotic function via IL-10 and IL-33 cytokine secretion [130]. In addition, IL-33, produced by macrophages and dendritic cells, activates the Th2 response, whereas Th2 cytokines (IL-4 and IL-13) in turn activate macrophages. Finally, macrophages and dendritic cells induce IgG4 class switching through B-cell-activating factor (BAFF) and a proliferation-inducing ligand (APRIL) secretion [132].

Cytotoxic T lymphocytes (CD4^+^CTLs) are proposed to have one of the key roles in fibrogenesis and inflammation by inducing apoptosis and stimulating innate immune response by macrophage activation. Activated macrophages clear apoptotic cells by efferocytosis and profibrotic function via IL-10 and IL-33 cytokine secretion [130]. Additionally, IL-33 that is produced by macrophages and dendritic cells might activate the Th2 immune response, whereas Th2 cytokines (IL-4 and IL-13) in turn activate macrophages. Accordingly, increased IL-33 and interferon-α, produced by dendritic cells, are reported in patients with definite type 1 AIP compared with chronic pancreatitis or healthy controls [62]. The pancreatic accumulation of these cells is also found in murine experimental AIP and human type 1 AIP [131]. Finally, macrophages and dendritic cells induce IgG4 class switching through the B-cell-activating factor (BAFF) and a proliferation-inducing ligand (APRIL) secretion [132]. The schematic model of AIP/IgG4 pathogenesis is shown in Figure 3.

## 5. Conclusions

To our knowledge, this is the first systematic review that addresses biomarkers in AIP. Evolving research has recognized numerous biomarkers that might help clarify the pathophysiological mechanisms of AIP. However, the specificity and sensitivity of these markers seem to be insufficient to serve as distinctive AIP evidence. Despite limited sensitivity, IgG4 remains the best available marker, with levels of >280 mg/dL as the most reliable AIP indicator. In addition to individual markers, panels of different markers appear as promising tools for early noninvasive differentiation between AIP and pancreatic cancer.

## Supplementary Materials

The following supporting information can be downloaded at: https://www.mdpi.com/article/10.3390/biomedicines10071511/s1, Supplemental Table S1. Role of IgG4 in diagnosing AIP; Supplemental Table S2. Relationship between serum biomarkers and EPL; Supplemental Table S3. Role of biomarkers in differentiating between AIC and PSC; Supplemental Table S4. Role of biomarkers in distinguishing type 1 AIP from type 2 AIP; Supplemental Table S5. The role of biomarkers in differentiating between AIP and pancreatic cancer; Supplemental Table S6. Miscellaneous biomarkers in AIP; Supplemental Table S7. Role of serum biomarkers in CST monitoring and the prediction of relapse; Supplemental Table S8. Role of autoantibodies in AIP; Supplemental Table S9. Role of Eosinophils and IgE in AIP.

## Abbreviations

AIP	Autoimmune pancreatitis
LPSP	Lymphoplasmacytic sclerosing pancreatitis
IDCP	Idiopathic duct centric pancreatitis (IDCP)
ICDC	International Consensus Diagnostic Criteria
IgG4	Immunoglobulin G 4
IgG4-RD	IgG4 related disease
GEL	Granulocytic epithelial lesion
CP	Chronic pancreatitis
CST	Corticosteroid therapy
CIC	Circulating immune complexes
PDAC	Pancreatic ductal adenocarcinoma
