# Supplementary material for: The Clinical Utility of Soluble Serum Biomarkers in Autoimmune Pancreatitis: A Systematic Review
Source: Biomedicines. 2022 Jun 26;10(7):1511. doi: 10.3390/biomedicines10071511 (PMC9312496; doi:10.3390/biomedicines10071511)
Supplement: Supplementary file 1 [file biomedicines-10-01511-s001.zip › biomedicines-1763439-supplementary.pdf]

Supplemental Table S1. Role of IgG4 in diagnosing AIP.

Author Year, Country	Candidate Biomarker	Diagnostic method	Cohort (n)	Criteria	Marker cutoff value	Frequency of marker elevation in AIP (%) vs. frequency in controls (%)	Mean/Median Sn, Sp, PPV, NPV	Conclusion
**Aoki (12) 2005, Japan**	γ- globulin, IgG, IgG4 ANA	N/A	AIP (n = 20)	JPS	IgG4 ≥ 135 mg/dL<br>IgG ≥ 1700 mg/dL<br>γ- globulin ≥ 1.25 g/dL	IgG4 was elevated in all pts (8/8), IgG in 61% (11/18), and γ-globulin in 75% (12/16). ANA was detected in 78.9% (15/19) of cases.	Mean serum γ-globulin was 2.14 g/dL, mean IgG was 2232 mg/dL, and mean IgG4 was 519.5 mg/dL.	The present immunohistochemical study shows the presence in the serum of an epithelial cell-specific auto-Ab that belongs to IgG4.<br>* Results of IHH analysis not displayed in this table.
**Choi (19) 2007, Korea**	IgG4, IgG	Nephelometry and single radial immunodiffusion	AIP (n = 35), ordinary CP (n = 67), pancreatic cancer (n = 76)	Kim	IgG > 1800 mg/dL<br>IgG4 > 135 mg/dL	IgG4 was increased in 11.9% (8/67), 1.3% (1/76), and 73.3% (22/30) of pts with CP, pancreatic cancer, and AIP, respectively. As for serum IgG levels, they increased in 10.4% (7/67), 2.6% (2/76), and 54.3% (19/35), respectively.	Median IgG:<br>AIP: 1880 mg/dL (range, 974-4570 mg/ dL);<br>CP: 1240 mg/dL (range, 713-2840 mg/dL);<br>Pancreatic cancer: 1245 mg/dL (range, 748-1940 mg/dL).<br><br>Median IgG4:<br>AIP: 473 mg/dL (range, 10-1764 mg/dL);<br>Ordinary CP: 47 mg/dL (range, 4-407 mg/dL);<br>Pancreatic cancer: 48 mg/dL (range, 2-180 mg/dL).<br><br>The IgG4 level at 141 mg/dL was the most optimal cutoff value with Sn and Sp of 73% and 95%, respectively (AUC 0.82), whereas for IgG, it was 1770 mg/dL, with Sn and Sp of 57% and 94% (AUC 0.79).	The Sn of serum IgG4 tended to be higher than that of IgG in the diagnosis of AIP. The IgG4 showed high Sp in the differential diagnosis of AIP from pancreatic cancer. Serum IgG4 should be included in the diagnostic workup for AIP.

**Ryu (75) 2008, Korea**	IgG, IgG4<br>ANA, RF	N/A	AIP (n = 67)	JPS<br>HISORt<br>Korean	IgG > 1800 mg/dL<br>IgG4 ≥ 135 mg/dL	IgG was elevated in 54% (34/67) of pts, and IgG4 was elevated in 25 pts (68%); 13 pts revealed isolated IgG4 elevation without IgG elevation, and either IgG or IgG4 elevation were detected in 76% (48/63) of pts. Auto-Ab was detected in 33% (22/67) of pts; ANA was detected in 23% of 57 pts, and RF was detected in 21% of 42 pts.	N/A	Among 67 cases of AIP, either IgG or IgG4 were elevated in 76% of pts, and 14 pts (21%) had OOI. New Korean diagnostic criteria are useful for the diagnosis of AIP.
**Frulloni (27) 2009, Italy**	IgG4<br>CA 19-9	Nephelometry	AIP (n = 87: focal type (n = 55); diffuse type (n = 32))	Histology, imaging, serology, OOI, response to steroids.	IgG4 > 135 mg/dL<br>CA 19-9 > 25U/I	Elevated IgG4 was observed in 50% (28/56) of pts. Serum levels of IgG4 were increased in 66% (22/33) of focal AIP and in 27% (6/23) of diffuse AIP (p = 0.006). Levels of CA 19-9 were increased in 25% (21/60) of pts, with no difference between focal and diffuse AIP.	The mean serum levels of IgG4 were higher in focal than in diffuse AIP (267.5 ± 332 mg/dL vs. 78.2 ± 65.8 mg/dL; p = 0.009). The highest observed value of CA 19-9 was 332 U/L.	Focal-type and diffuse-type AIP differ with regard to clinical symptoms and signs. Recurrences occur more frequently in focal AIP than in diffuse AIP. The OR to develop recurrences was 3.9 (CI 1.4 – 10.9) for smoking (no / yes) and 6 (CI 1.6 – 21.8) for IgG4 (< 135 mg / dL vs. >135 mg / dL).
**Raina (73) 2009, USA**	IgG, IgG4	Nephelometry	AIP (n = 26)	JPS<br>HISORt<br>Korean	IgG4> 140 mg/dL	IgG4 was elevated in 44 % (8/18) of pts at presentation. An increase in serum IgG4 level to > 140 and > 280 mg / dL was observed in 44% (8/18) and 28% (5/18) of pts, respectively. ANA was	N/A	The proportion of pts who had an increase in IgG4 (> 140 mg/dL in 44% and > 280 mg/dL in 28%) was lower than previously reported. Clinically significant vascular complications

						increased in 50% (10/20) of cases. CA 19-9 was increased in 27 % (4/15) of pts.		occurred in a subset of AIP pts.
**Tabata (87) 2009, Japan**	IgG4	Nephelometry	AIP (n = 39), CP (n = 18), idiopathic pancreatitis (n = 8), pancreatic cancer (n = 116), bile duct cancer (n = 34), sclerosing cholangitis (n = 4), Mikulicz's disease (n = 18), Sjogren's syndrome (n = 32), sialolithiasis (n = 10). Controls (n = 189) pts with other diseases except pancreas.	Asian	IgG4 > 135 mg/dL	IgG4 was increased in 77% (30/39) of AIP pts, 6% (1/18) in CP, 38% (3/8) in idiopatic pancreatitis, 4% (5/116) in pancreatic cancer, 6% (2/34) in bile duct cancer, 25% (1/4) in sclerosing cholangitis, 100% (18/18) in Mikulicz's disease, 0% (0/32) in Sjogren's syndrome, 0% (0/10) in chronic sialadenitis and 8% (15/189) in the control groups.	The median IgG4 concentration in AIP was 301.5 mg/dL and was greater than in other pancreato-biliary diseases (p <0.01), except for Mikulicz's disease (p = 0.22). Sn and Sp for increased serum IgG4 (>135 mg/dL) for diagnosis of AIP were 77% and 96%, respectively. To distinguish AIP from pancreatic cancer at optimal IgG4 cutoff value of >119 mg/dL, the Sn was 82%, the Sp was 95%, and the AUC was 0.90.	The serum IgG4 concentration was significantly greater in AIP than in other pancreato-biliary diseases. The optimal serum IgG4 cutoff value to distinguish AIP from pancreatic cancer was 119 mg/dL.
**Song (84) 2010, South Korea**	IgG, IgG4, ANA, RF	Nephelometry	AIP (n = 82) , pancreatic cancer (n = 110)	Asian HISORt	IgG4 ≥ 135 mg /dL IgG ≥ 1800 mg /dL ANA ≥ 1:80 RF	In pts with AIP, IgG was increased in 46.3% (38/82), IgG4 in 52.4% (43/82), and combined (either IgG4 or IgG) in 68.2% (56/82). An isolated elevation of IgG4 with normal total IgG was seen in 21.9% (18/82), whereas 15.8% of pts (13/82) had isolated IgG elevation despite normal IgG4.<br><br>In pts with pancreatic cancer IgG was increased in 3.6% (4/110), and IgG4 in 0.9 % (1/110). All but one	AIP vs. pancreatic cancer Mean total serum IgG level: 2019.2 ± 1026.0 mg/dL vs. 1230.0 ± 277.3 (p < 0.05) Mean total serum IgG4 level: 310.3 ± 408.3 mg/dL vs. 47.3 ± 36.8 md/dL (p < 0.05).<br><br>To diagnose AIP, the Sn of IgG4 (52.5%) was higher than that of IgG (46.3 %), (p < 0.05). The Sn of the combined measurement of IgG and IgG4 for AIP was 68.3 %, higher than that of IgG4 alone (p < 0.05). In the differentiation of AIP from pancreatic cancer, the Sp of IgG and IgG4 was 96.4 and 99.1 %, respectively.	The combined measurement of total serum IgG and IgG4 may increase diagnostic Sn without sacrificing Sp, compared with IgG4 alone. However, the measurement of ANA or RF may show no additional benefit when combined with total serum IgG and IgG4.

						patient who had positive results for ANA or RF also showed elevations of either total IgG or IgG4, respectively.	The Sp of the combined measurement of total IgG and IgG4 was 95.5 %, and it was NS different from that of IgG4 alone (p = 0.125). The Sn of ANA (≥ 1:80) and RF was 24 % and 20 %, respectively.	
**Kamisawa (48) 2011, Japan, Korea, Taiwan, China, and India**	IgG, IgG4	N/A	AIP (n = 327)	Asian	IgG4 ≥ 135 mg/dL<br>IgG ≥ 1800 mg/dL	Elevation of serum IgG was detected frequently in Japan (60% (74/124) compared with Taiwan (32% (15/47); p < 0.01). Elevated serum IgG4 was more frequent in Japan (86% (95/111) and Taiwan (100% (28/28) compared with Korea (58% (50/86); p < 0.01). ANA was present in 24% to 40%, and RF was present in 17% to 33% of pts.	The average serum IgG4 level was 336.8 mg/dL in Korea, 524.9 mg/dL in Japan, and 729.3 mg/dL in Taiwan.	The features of AIP are fundamentally similar in Japan, Korea, Taiwan, and China.
**Matsubayashi (59) 2011, Japan**	IgG4, IgG, ANA and RAPA, AMA and SMA, SS-B, anti-DNA Ab, SS-A and anti-RNP Ab	N/A	AIP n = 27: high IgG4 (n = 20); normal IgG4 (n = 7)	JPS 2006 Asian	Serum IgG ≥ 1800 mg/L<br>IgG4 ≥ 135 mg/dL<br>IgG1 ≥ 748 mg/dL<br>ANA and RAPA: ≥ 80-fold<br>AMA and SMA: ≥ 40-fold<br>SSB: > 10 U/mL<br>anti-DNA Ab: > 6	74% (20/25) had elevated serum IgG4 and 26% (7/25) had normal IgG4 levels. Serum IgA and IgM in high-IgG4 AIP were slightly lower than those in normal IgG4 AIP, but the difference was NS. Amongst analyzed antibodies, only RAPA, ANA, SS-A, SS-B and SMA were positive (p = 0.33). Eosinophilia was relatively more	Normal IgG4 vs. high IgG4 AIP:<br>Mean IgG4: 48.0 ± 37.1 vs. 516.5 ± 302.4 (p = 0.0001);<br>Mean IgG: 1318.9 ± 405.2 vs. 1883.9 ± 431.9 (p = 0.006);<br>Mean IgG1: 679.9 ± 170.0 951.5 ± 243.5 (p = 0.01).<br><br>Compared to pts with normal serum IgG4 levels, pts with elevated IgG4 had a higher incidence of jaundice at onset (14.3% vs. 80%, respectively; p = 0.002).<br>When all the lesions at all sites	Clinical features of AIP are different based on level of serum IgG4. Compared to pts with normal serum IgG4, pts with elevated IgG4 had higher incidence of jaundice at onset, more frequent diffuse pancreatic enlargement at imaging, and more frequent EPL.

					IU/mL,<br>SS-A and anti-RNP Ab: any positive	frequent in high-IgG4 AIP (45%) than in normal IgG4 (14.3%) (p = 0.15).	were included, the frequency of EPL was significantly higher in the high IgG4 group than in normal IgG4 group (85% vs. 42.9%, respectively; p = 0.03).	
**Sadler (76) 2011, UK**	IgG, IgG4	Nephelometry	AIP (n = 21: IgG4+ (n = 19); IgG4- (n = 2)), cancer (n = 57: pancreatic, cholangio, and gallbladder adenocarcinoma). Control group: pancreatitis (n = 67: acute (n = 22); chronic (n = 32); alcoholic (n =13)). PSC (n = 51).	HISORt	IgG >13 g/L<br>IgG4 > 1.3 g/L	About 62% (13/21) of AIP pts and 16% (9/57) of cancer pts had increased total serum IgG. In the AIP group, only 9.5% of pts (2/21) gave normal serum IgG4 levels, whereas the cancer group showed 91% (52/57) of its pts to have an IgG4 level below 1.3 g/l.	The mean IgG in AIP was significantly higher compared to all other groups (19.0 g/L ± 2.5, p < 0.001). The mean serum IgG4 for AIP (3.7 g/L ± 0.5) was higher than in other groups (mean IgG4 levels <1 g/L,p < 0.001).<br><br>IgG4 for detecting AIP: Sn 91%, Sp 92% if all samples were included. IgG to determine AIP: Sn of 62% and Sp of 59%. Malignancy vs. AIP: 91 % Sn, 91% Sp. PPV was 79% if IgG4 was >1.3 g/L.	Circulating IgG4 levels are an accompanying diagnostic marker to imaging, histology and clinical presentation. This may help in differentiating between AIP and pancreatic carcinoma.
**Tabata (88) 2012, Japan**	IgG4, IgG<br>IgE,<br>peripheral Eo<br>ANA, RF	Nephelometry	AIP (n = 67: D-type (n = 34), H-type (n = 19), B/T type (n = 14))	Asian	IgG4 > 135 mg/dL<br>IgE > 580 IU/L<br>Eosinophil > 600/μl	Serum IgG4 levels were more frequently elevated in D-type 88% (28/32) than in B/T type 50% (7/14) pts (p = 0.03). There was NS differences in the number of pts with elevated IgE and peripheral Eo counts among the groups. No differences for ANA and RF either.	IgG4 levels were significantly higher in D-type (median 309 mg/dL) than in B/T-type (133.5 mg/dL) pts (p = 0.042). There were no significant differences in IgE levels or peripheral Eo counts among the groups. No differences for ANA and RF either.	The serum IgG4 level was significantly lower in B/T-type than in D-type pts, and the elevation of serum IgG4 levels was less in B/T type than in D type. B/T-type AIP may involve a disease other than LPSP.
**Chatterjee (18) 2014, UK**	IgG, IgG4<br>CA 19-9	N/A	AIP (n = 22)	HISORt	IgG4 ≥ 2.4 g/L<br>CA 19-9 ≥ 33 kU/L	Total IgG was elevated in 10 pts and 64% (14/22) had an elevated IgG4 level. CA 19-9 was elevated in 73% (16/22) of the pts.	Mean IgG was 19.96 g/L (range 15.8-42), mean IgG4 was 10.5 g/L (range 3.4–31 g/L), and mean CA 19-9 was 262 kU/L (range 1–2127 kU/L).	Diagnosis of AIP should be based on accepted criteria as this significantly reduces the chances of overlooking

								malignancy. Steroid responsiveness is characteristic but should never be used as a substitute to an aggressive search for malignancy.
**Xin (97) 2014, China**	IgG, IgG4 ANA, anti-SSA, anti-SSB	N/A	AIP (n = 100)	Asian HISORt ICDC	IgG > 15.0 g/L IgG4> 2.0 g/L	Serum IgG > 15.0 g/L was seen in 69.4% (43/62) pts and increased IgG4 levels in 92% (69/75), with 81.1% (56/75) having IgG4 > 4.0 g/L. The positive rate of IgG4 was higher than that of IgG (92.9% vs. 71.4%, p = 0.008). ANA, anti-SSA Ab, and anti-SSB Ab were positive in 17.1% (6/35), 11.4% (4/35) and 8.6% (3/35) of pts, respectively, with all of them showing elevated serum IgG4.	N/A	Clinical manifestations, laboratory tests, imaging and pathological examinations could increase the diagnostic rate of AIP. Autoimmune antibodies (ANA, anti-SSA, anti-SSB) only play a suggestive role when serum IgG4 is not available.
**Kanno (51) 2015, Japan**	IgG4, IgG ANA, RF	N/A	AIP (n = 936: 77.5% definitive (n = 725); 4.5% probable (n = 42); 10.7% possible (n = 100); 6.8% not diagnosed (n = 64); and 0.5% N/A (n = 5)).	JPS 2011	IgG4 ≥ 135 mg/dL IgG ≥ 1800 mg/dL ANA ≥ 80-fold RF ≥ 20 IU/mL	IgG4 was elevated in 86.4% (739/936), 56.4% (486/862) had high levels of IgG, ANA was positive in 33.5% (263/785), and 21.7% (125/576) were positive for RF.	Mean IgG4 533.0 ± 540.9 mg/dL	Of the 761 pts, the initial dose was 40 mg/d in 146 (19.2%) pts, 30 mg/d in 478 (62.8%), 20 mg/d in 35 (4.7%), and other doses in 102 (13.4%). Maintenance steroid treatment was performed for 644 (84.6%) pts.

IgG4: immunoglobulin G4; AIP: autoimmune pancreatitis; Sn: sensitivity; Sp: specificity; PPV: positive predictive value; NPV: negative predictive value; IgG: immunoglobulin G; CP: chronic pancreatitis; pancreatic cancer: pancreatic cancer; AUC : area under the curve; PSC: primary sclerosing cholangitis; ANA: anti-nuclear antibodies; RF: rheumatoid factor; IgE: immunoglobulin E; Eo:

eosinophils; D type: diffuse type; H type: focal head type; B/T type: focal body and/or tail type; JPS: Japan Pancreas Society; LPSP: lymphoplasmacytic sclerosing pancreatitis; IHH: immunohistochemistry; anti-SSA: anti-Sjögren's syndrome-related antigen A; anti-SSB: anti-Sjögren's syndrome-related antigen B; ICDC: international consensus diagnostic criteria; CA 19-9: cancer antigen 19-9; OOI: other organ involvement; CI: confidence interval; N/A: not available; RAPA: rheumatoid arthritis particle agglutination; SMA: anti-smooth muscle antibody; AMA: anti-mitochondrial antibodies; anti-RNP: anti-nuclear ribonucleoprotein antibodies; IgM: immunoglobulin M; EPL: extrapancreatic lesions; NS: not significant.

Supplemental Table S2. Relationship between serum biomarkers and EPL.

Author Year, Country	Candidate Biomarker	Detection method	Cohort (n)	Criteria	Marker cutoff value	Frequency of marker elevation in AIP (%) vs frequency in controls (%)	Mean (±SD)/Median Sn, Sp, PPV, NPV	Extrapancreatic lesions (EPL)	Conclusion
**Hamano et al. (32) 2007, Japan**	IgG4, CIC	Single radial immunodiffusion, the monoclonal RF method	AIP (n=64)	JPS	IgG4 > 135 mg/dL CIC > 4.2 μg/mL	A high IgG4 concentration was found in 92% (59/64) of pts; 5 pts with normal IgG4 concentrations had either ANA or RF. A high serum CIC concentration was found in 67% (43/64) of pts.	A high IgG4 concentration was found in 92% of pts with a median IgG4 concentration of 617.5 mg/dL. Median serum CIC concentration was 6.45 μg/mL.	Number and type of OOI (hilar lymphadenopathy or lachrymal and salivary gland lesions) were positively associated with IgG4 level.	Pts with hilar lymphadenopathy or lachrymal and salivary gland lesions had higher IgG4 levels than those without (p = 0.004 and 0.02, respectively). Pts with 3 lesions had higher IgG4 levels than those with no lesions (p=0.049), and pts with multiple lesions tended to have higher IgG4 values than those with few lesions. No significant differences in CIC levels in relation to number of EPL were found.
**Kamisawa et al. (46) 2008, Japan**	IgG4	Single radio diffusion	AIP (n=40)	Histology, imaging, serology, response to steroids	IgG4 ≥ 135 mg/dL IgG4 ≥ 220 mg/dL (AIP with EPL vs. AIP	32 pts had serum IgG4 levels greater than 135 mg/dL, and 8 pts had serum IgG4 < 135 mg/dL.	The mean serum IgG4 level of the 40 AIP pts was 411±448 mg/dL. On the basis of the ROC curve data, the optimal serum IgG4 cutoff value to	AIP pts with serum IgG4 levels of more than or equal to 220 mg/dL frequently have EPL.	78% (18/23) had EPL, whereas 24% (4/17) of pts whose serum IgG4 was less than 220mg/dL had EPL (p=0.001). Sclerosing cholangitis and sclerosing cholecystitis were

					without EPL)		distinguish between AIP pts with and without EPL was 220 mg/dL (Sn 81%, Sp 72%, AUC 0.81).		more frequent in pts with serum IgG4 > 220 mg/dL than in those with lower IgG4 (p=0.0002 and 0.02, respectively). The number of associated EPL was significantly greater in pts with high IgG4 (p=0.0003)
**Igarashi et al. (39) 2012, Japan**	IgG, IgG4	N/A	Type 1AIP (n=41) and IgG4-RD without pancreatic lesions (n= 5)	JPS Asian HISORt	IgG4 level ≥ 1.35 g/L	N/A	Serum IgG level (g/L, mean ± SD) 21.25 ± 9.85 Serum IgG4 level (g/L, mean ± SD) 6.68 ± 6.27.	A correlation between serum IgG4 level and the number of regions involved was found. Serum IgG4 was higher in pts with multiple lesions when accompanied by sclerosing sialadenitis.	Mean serum IgG levels were 15.1, 18.7, 20.9, 23.3 and 31.0 g/L, while the mean IgG4 levels were 4.0, 4.7, 4.7, 9.9 and 16.5 g/L in group A (one region involved), B (two regions involved), C (3 regions), D (4 regions) and E (5-7 regions), respectively. IgG4 was positively correlated (r=0.61) with the number of regions involved (p = 0.001). Serum IgG4 was higher in pts with multiple lesions when accompanied by sclerosing sialadenitis.
**Kaji et al. (44) 2012, Japan**	IgG4	Nephelometry	Type 1 AIP pts (n=35) and non-AIP pts (n=71: pancreatic cancer (n=17); 24 CP (n=24); PSC (n=7); and biliary system cancer (n=23))	Asian ICDC	IgG4 ≥ 135 mg/dL (AIP vs. non-AIP) IgG4 ≥ 346 mg/dL (abdominal vs. systemic AIP)	Elevated IgG4 was found in 91.4% (32/35) of type 1 AIP, 0% (0/17) of pancreatic cancer, 4.2% (1/24) of CP, 0% (0/6) of PSC, and 4.3% (1/6) of bile system cancer.	The medians (range) for serum IgG4 in type 1 AIP and non-AIP were 336.0 (63.6–2740) mg/dL and 29.8 (3–243) mg/dL, respectively. IgG4 in type 1 AIP was higher than those of IgG4 in non-AIP (p< 0.001).<br><br>The IgG4 (≥ 135 mg/dL) for differentiating type 1 AIP from non-AIP had 91% Sn, and 97% Sp. Serum IgG4 (≥ 346 mg/dL) for	Serum IgG4 was useful in both the diagnosis of type 1 AIP and the detection of systemic EPL.	EPL had 94.3% (33/35) pts among type 1 AIP, with correlation between serum IgG4 and the number of EPL (r= 0.75, p < 0.001). Both serum IgG4 and total numbers of EPL in systemic EPL were remarkably higher than those in abdominal localized EPL. Serum IgG4 cutoff value was 346 mg/dL to distinguish between abdominal localized EPL and systemic EPL.

							distinguishing between abdominal localized EPL and systemic EPL had 79% Sn, 88% Sp, and an AUC of 0.89.		
**Suzuki et al. (86) 2018, Japan**	IgG4	N/A	AIP (n = 73)	ICDC	IgG4: normal (< 135 mg/dL); level 1 (≥ 270 mg/dL); level 2 (135–269 mg/dL).	There were 39 (95%) cases in the level 1 group, 2 (12%) cases in the level 2 group, and 0 (0%) cases in the normal group. Extrapancreatic lesions were observed in 30 (73%) cases in the level 1 group, 8 (47%) cases in the level 2 group, and 4 (27%) cases in the normal group, and there was a significant difference between the level 1 group and the normal group.	When the cutoff value for the presence or absence of EPL was set to 316 mg/dL, the sensitivity and specificity were 69% and 68%, respectively. The AUC was 0.71.<br><br>The average number of EPL was 1.0 in the level 1 group, 0.5 in the level 2 group, and 0.3 in the normal group. The average number of extrapancreatic lesions was significantly higher in the level 1 group.	Elevated serum IgG4 levels are closely associated with the existence of multiple IgG4-related EPL, but not with an increased risk of relapse. However, posttreatment serum IgG4 rises of more than 1.25 times during the follow-up period might be predictive of relapse. In cases where such rises are detected, the tapering or withdrawal of CST should be carefully considered.	Sclerosing cholangitis (30%) and sclerosing dacryoadenitis/sialadenitis (25%) were most commonly observed.
**Ishikawa et al. (42) 2021, Japan**	IgG, IgG4	N/A	AIP (n=153: definitive type 1 (n=134); probable type 1	ICDC	IgG > 1700 mg/dL IgG4 > 135 mg/dL IgG4 > 270 mg/dL	The serum IgG4 level was elevated in all pts in the KD group, and 15 pts (82.4%) had	The median serum IgG and IgG4 levels were significantly higher in the KD group than in the non-KD group (2214 (1928–2896) vs.	IgG4-RKD was identified as an independent factor for serum IgG4 elevation (> 270 mg/dl) and	IgG4-RKD was identified as an independent factor for serum IgG4 elevation (> 270 mg/dl) and relapse compared with other organ involvement.

			(n=17); NOS (n=12)).<br><br>Kidney disease group (n = 17), and non-kidney disease group (n = 136).			a value of at least 270 mg/dL, which is twice the upper limit of normal.	1747 (1374–2061) mg/dL, p = 0.001; and 663 (IQR 340.5–1036) vs. 304.5 (IQR 145.5–618.25) mg/dL, p = 0.014.)	relapse compared with other organ involvement.	
**Rogger et al. (74) 2021, Italy**	IgG4 in IgG4 -RI	N/A	Type AIP 1 (n=33)	ICDC	IgG4 ≥ 135 mg/dL IgG4 ≥ 240 mg/dL	Serum IgG4 concentration was increased in 94% (31/33) pts and normal in 6% (2/33)	Median serum IgG4 was 413 ±303 mg/dL. Mean IgG4 was lower at time 1 compared with time 0 (209±173 vs. 413±303 mg/dL, respectively; p < 0.0001) but not at time 2 compared with time 1 (251±245 mg/dL vs. 209 ±173 mg/dL, respectively; p = 0.37).	Serum IgG4 level, both before and after CST, seemed to have no role as a prognostic surrogate marker, as it was not associated with disease activity or risk of disease relapse, using both absolute value and different cutoffs, despite a significant reduction in serum IgG4 after CST.	Serum IgG4 did not correlate with IgG4-RD RI or EPL at time 0.

EPL: extrapancreatic lesions; AIP: autoimmune pancreatitis; Sp: specificity; Sn: sensitivity; PPV: positive predictive value; NPV: negative predictive value; SD: standard deviation; IgG4: immunoglobulin G4; CP: chronic pancreatitis; PSC: primary sclerosing cholangitis; ICDC: international consensus diagnostic criteria; AUC: area under the curve; ROC: receiver operating characteristics; RI: responder index; N/A: not available; CST: corticosteroid treatment; IgG: immunoglobulin G; NOS-AIP: not-otherwise-specified AIP by ICDC; IQR: interquartile range; CIC: circulating immune complexes; RF: rheumatoid factor; JPS: Japan Pancreas Society; ANA: anti-nuclear antibodies.

Supplemental Table S3. Role of biomarkers in differentiating between AIC and PSC.

Author Year, Country	Candidate biomarker	Marker cutoff value	Criteria	Cohort (n)	Mean/Median Sn, Sp, PPV, NPV	Frequency of marker elevation in AIP (%) vs. frequency in controls (%)	Conclusion
**Nakazawa et al. (67) 2005, Japan**	Peripheral Eo count IgG, IgG4, ANA	Eo > 6.0% IgG ≥ 1700 mg/dL IgG4 ≥ 135 mg/dL ANA positive	JPS	PSC (n = 27), 20 AIP (n = 20)	AIP– AIC vs. PSC (mean): Eo: 3.1±2.0 vs. 7.1±6.0, p < 0.01; IgG: 2035±912 vs. 2210±686, NS; IgG4: 234±172 vs. 32.5±29.0, p < 0.01;	PSC vs. AIP– AIC: ANA (positive): 14/19 (73.7%) vs. 12/27 (44.4%), p < 0.05.	Abrupt obstructive jaundice, IgG4 and other organ involvement present at AIC, whereas PSC presents more often with right-sided ulcerative colitis and no IgG4 elevation.
**Hirano et al. (33) 2006, Japan**	IgG4	IgG4 ≥ 135 mg/dL	JPS	AIP (n =35), non-AIP CP (n = 24), PSC (n = 11), pancreatic cancer (n = 23), islet cell tumor (n = 3), papilla cancer (n = 2), bile duct cancer (n = 15), and gallbladder cancer (n = 3)	Serum IgG4 [mean ±SD (range)]: AIP: 532±405 (65.2– 2170); non-AIP CP: 37.2±20.9 (6.8–67.2); pancreatic cancer: 38.1±24.7 (9.8–98.5); islet cell tumor: 26.9±13.3 (8.6–39.8); papillary cancer: 38.1±24.7 (9.8–98.5); PSC: 186±241 (10.1– 828); bile duct cancer: 62.4 37.8 (12.5–127); gall bladder cancer: 46.8±20.9 (6.8–67.2).	Increased serum IgG4 was observed in 95% (33/35) of pts with AIP and in 36% (4/11) with PSC. No IgG4 elevation was observed in pts with CP (0/24), pancreatic cancer (0/23), islet cell tumor (0/3), duodenal papilla cancer (0/2), bile duct cancer (0/15), or gallbladder cancer (0/3).	Serum IgG4 was increased in AIP but remained within normal range in other conditions except for PSC. Serum IgG4 concentrations are useful for differentiating between AIP and other conditions. CST has been considered to be contraindicated in pts with PSC because the therapy is generally ineffective and causes biliary infection. However, it is worthy of consideration that CST might be effective in those cases with increased IgG4.
**Nishino et al. (70) 2007, Japan**	IgG4 Peripheral Eo count	IgG4 < 135 mg/dL	JPS	AIP– AIC (n =24), PSC (n = 24)	The peripheral Eo count was significantly higher in the PSC group, and the serum IgG4 values were significantly higher in the SC–AIP group (463±355 mg/dl vs. 32± 10 mg/dl, p = 0.003).	ANA were positive in 80% of the SC–AIP pts and in 56% of the PSC pts (NS).	Based on the pts' age, blood chemistry findings, associated diseases, cholangiographic findings, liver biopsy findings, and clinical course, SC–AIP and PSC are different clinicopathological entities, and SC–AIP should be differentiated from PSC.
**Vujasinovic et al. (96) 2020, Sweden**	IgG4, IgG 2	Total IgG > 14.5 g/L IgG1 > 8.0 g/L IgG2 > 5.7 g/L	ICDC	PSC (n = 73), AIP (n = 69: AIP without AIC (n= 14); AIP with AIC (n = 55))	PSC vs. AIP (mean ±SD): IgG2 (g/L): 3.3±1.2 vs. 5.1±2.4, p < 0.0001; IgG4 (g/L): 0.4±0.4 vs. 2.0±3.9, p < 0.0001.	PSC vs. AIP: IgG2 (> 5.7 g/L): 2 vs. 21, p < 0.0001; IgG4 (> 1.25 g/L): 3/73 (4.1%) vs. 30/69 (43.5%), p < 0.0001; High IgG2 or high IgG4: 5 vs.	High IgG2 or IgG4 levels identify pts with AIP, while high IgG1 in those with low or normal IgG2, and IgG4 levels identifies pts with PSC.

	IgG3 > 1.25 g/L<br>IgG4 > 1.25 g/L		AIP without AIC vs. AIP with AIC (mean ±SD):<br>IgG2 (g/L): 4.6±2.0 vs. 5.2±2.4, p = 0.57;<br>IgG4 (g/L): 1.2±1.2 vs. 2.2±4.3 p = 0.76.<br><br>High IgG2 has a high Sp (97%) and PPV (91%) to identify pts with AIP, but a low Sn (31%). The combination of high IgG2 and IgG4 retains similar Sp (93%) and PPV (89%) but increases the Sn to 57%.	39, p < 0.0001.<br><br>AIP without AIC vs. AIP with AIC:<br>IgG2 (> 5.7 g/L): 3 vs. 18, p = 0.52;<br>IgG4 (> 1.25 g/L): 5/14 (35.7%) vs. 25/55 (45.5%), p < 0.0001;<br>High IgG2 or high IgG4: 6 vs. 33, p < 0.0001.

AIC: autoimmune cholangitis; PSC: primary sclerosing cholangitis; Sp: specificity; Sn: sensitivity; PPV: positive predictive value; NPV: negative predictive value; IgG4: immunoglobulin G4; JPS: Japan Pancreas Society; CST: corticosteroid treatment; CP: chronic pancreatitis; IgG2: immunoglobulin G2; ICDC: international consensus diagnostic criteria; SD: standard deviation; IgG: immunoglobulin G; ANA: anti-nuclear antibodies; NS: not statistically significant; Eo: eosinophils; SC-AIP: sclerosing cholangitis with autoimmune pancreatitis.

Supplemental Table S4. Role of biomarkers in distinguishing type 1 AIP from type 2 AIP.

**Author, Year, Country**	**Candidate Biomarker**	**Diagnostic method**	**Cohort (n)**	**Criteria**	**Marker cutoff value**	**Frequency of marker elevation in AIP (%) vs. frequency in controls (%)**	**Mean/Median Sn, Sp, PPV, NPV**	**Differences in marker levels in relation to clinical presentation**	**EPL**	**Steroid treatment response**	**Relapse rate**	**Steroid regimen**	**Conclusion**
**Kamisawa (49)<br>2005, Japan**	IgG4	Single radial immunodiffusion	AIP (n= 21, elevated IgG4 (n=14) and low IgG4 (n=7))	Histology, imaging, serology,	> 135 mg/dL (elevated vs. low)	Serum IgG4 concentrations were elevated in 667% (14/21) pts	High vs. low serum IgG4.<br><br>528 (280–1125) vs. 39 (34–	N/A	NS differences in the frequency of biliary stenosis, the presence of auto-Ab	N/A	N/A	Initial dose of 30 to 40 mg/d oral PSL. Tapering by 5 to	IgG4-related phenomena occurred in various organs of pts with AIP associated

	steroid trial		and ranged from 11 to 128 mg/dL in the other 7 pts.	119) $p < 0.01$.		(ANA or RF), or palpable salivary gland enlargement between the 2 groups. Abdominal lymphadeno pathy was more frequent in pts with high IgG4 ($p < 0.05$). Although there was no difference in number of IgG4-positive plasma cells infiltrating the pancreas, pts with elevated IgG4 tended to have more of these cells infiltrating the wall of the bile duct, abdominal lymph nodes, and the gastric mucosa ($p < 0.01$).			2.5 mg every 1 to 2 wks; MST: 5 mg/d of oral PSL.	with elevated IgG4, whereas these phenomena tended to be rather confined to the pancreas in pts with low serum IgG4.

**Sah (77) 2010, USA**	IgG, IgG4	N/A	Type 1 AIP (n=78, histology confirmed in 50 cases); type 2 AIP (n=19).	HISORt	IgG4 > 140 mg/dL	Greater prevalence of increased serum levels of IgG4 in type 1 AIP than in type 2 AIP (80% (47/59) vs. 17% (1/6); p <0.004).	N/A	N/A	Pts with type 1 AIP were more likely than those with type 2 AIP to have a higher prevalence of OOI (60% vs. 0%; p <0.0001) and association with IBD (6% vs. 16%; p<0.37).	N/A	During the median clinical follow-up periods of 42 and 29 months, 47% (37/78) of pts with type 1 and none of those with type 2 experienced a relapse (p<0.0001).	Initial therapy: oral PSL of 40 mg/d for 4 wks. Tapering: 5 mg/wk until gone.	Types 1 and 2 AIP have distinct clinical profiles. Pts with type 1 AIP have a high relapse rate, but pts with type 2 AIP do not experience relapse.
**Kamisawa (50) 2011, Japan**	IgG4, IgG, IgE, peripheral Eo count	Nephelometry	AIP (n= 58: IgG4-positive (n=45), IgG4-negative (n=13))	Asian	IgG4 > 135 mg/dL (IgG-positive vs. IgG4-negative) Serum IgE > 580 IU/ml Eosinophils > 600/mm³	Serum IgG4 elevated in 78% (45/58) AIP pts. There were no differences in presence of auto-Ab, serum IgE, and amylase levels, and peripheral	Mean serum IgG4 in IgG4-positive pts (n=45): 604.2 ± 526.0 mg/dL. Mean IgG4 in was 62.4 ± 40.5 mg/dL in the IgG4-negative pts (n=13).	As an initial symptom, obstructive jaundice was significantly more frequent in IgG4-positive AIP pts (71%, p = 0.002),	Sclerosing EPL, especially sclerosing cholecystitis and sclerosing sialadenitis, were frequently detected in IgG4-positive AIP pts (51%, p = 0.008). Acute pancreatitis was more	CST was effective in both groups (100% response). IgG4-positive AIP pts frequently underwent CST, and IgG4-negative AIP pts were sometimes	Relapse was detected only in IgG4-positive pts (14%, 5/36). No pts in IgG4-negative group (n=6) relapsed.	Initial therapy: 0.6 mg/kg/d of oral PSL, tapering gradually to a maintenance dose over a period of 3–6 months. MST: (2.5 mg–5	Clinicopathological features of IgG4-negative AIP differed from those of IgG4-positive AIP.

						Eo count between IgG4-positive nad IgG-negative group.	Mean serum IgG was higher in IgG4-positive group (2344.8 ± 966.3 than in IgG4-negative group 1396.2 ± 277.4 (p< 0.001).	and abdominal pain was more frequent in IgG4-negative AIP pts (38%, p = 0.01)	frequent in IgG4-negative AIP pts (23%, p = 0.45). Ulcerative colitis was associated in one IgG4-positive and one IgG4-negative AIP patient. There were NS differences in frequencies of DM and pancreatic PEI.	followed conservatively (31%, p = 0.019). Six IgG4-positive AIP pts who were followed conservatively at first were later treated with steroids because of AIP exacerbation.		mg/day) was performed for 1–3 years.	
**Balasubramanian (6) 2012, USA**	IgG4 Peripheral Eo	N/A	AIP (n=133): AIP 1 (n=90 seropositive; (n=69) seronegative (n=21)); AIP 2 (n=22).	HISORt ICDC	> 135 mg/dL (seropositive vs. seronegative) Eo > 500 cells/cu. mm	Of all subjects included, 52% (69/133) were classified as seropositive type 1 AIP, 16% (21/133) as seronegative type 1 AIP, and 17% (22/133) as type 2 AIP.<br><br>Eosinophil	IgG4 (mean± SD): Seropositive AIP1 618.9±604 Seronegative AIP1 63.4 ± 35.6 AIP2 51.3± 45.1	Seropositive type 1 AIP pts presented more frequently with obstructive jaundice when compared to that of type 2 AIP pts (77% (53/69) vs. 55% (12/22), p =0.03)) compare	A similar proportion of seropositive (51/69 (73.9%)) and seronegative (15/21 (71.4%)) type 1 AIP pts had other organ involvement, while none of the type 2 AIP pts had EPL (0/22 (0%)). IBD was seen in 3/22 (13.6%) pts with type 2 AIP. The seropositive	N/A	Among seropositive type 1 AIP, 31/69 (45%) suffered clinical relapse during follow-up compared to 7/21 (33%) of seronegative type 1 AIP pts. This difference was NS	Prednisone 40 mg orally for 4 wks followed by a 5 mg/wk taper.	Seronegative and seropositive type 1 AIP pts have similar clinical profiles which are distinct from that of type 2 AIP. Among the seronegative AIP group, pts are more likely to have type 1 AIP rather than type 2 AIP if they are older than 50 years or have EPL

						ia: The prevalence of eosinophilia was similar in seropositive type 1 AIP (15/69 (22%)), seronegative type 1 AIP (5/21 (24%)) and type 2 AIP (4/22 (19%))		d to that of seronegative type 1 AIP pts (77% (53/69) vs. 62% (13/21), p =0.14)). The proportion of pts in each group presenting with pancreatitis was similar. There was no difference in the prevalence of DM or new onset DM between the groups.	and seronegative type 1 AIP groups had 2 pts each, with associated IBD. There was no significant difference in the occurrence of associated IBD between all three groups.		(p = 0.25). None of the type 2 AIP pts suffered a relapse. Hence, there was a difference in disease relapse in seropositive and seronegative type 1 AIP when compared to type 2 AIP (p < 0.001 and p< 0.005, respectively).		or disease relapse.
**Paik (7) 2013, South Korea**	IgG4	N/A	Type 1 AIP (n=25): IgG4-positive type 1 AIP (n=17); IgG4-negative	ICDC	IgG4 > 134 mg/dL	There were 68% (17/25) pts with IgG4-positive type 1 AIP and 32% (8/25)	The median serum IgG4 level was 312 mg/dL (normal range, 145-4000	N/A	The pts of the IgG4-positive group were more likely to have OOI than those who were seronegative (59% vs. 26%,	All pts, except those who received surgical resection, received CST and the	The relapse rate was not different between the two groups (36% vs.	CST was performed at 0.6 mg/kg per day of PSL for one mo and gradually	The Sn of serum IgG4 was not sufficient (68%) for defining type 1 AIP. The demographic findings were

			type 1 AIP (n=8)			pts with IgG4-negative type 1 AIP.	mg/dL) in the IgG4-positive group and was 33 mg/dL (normal range, 6-75 mg/dL) in the IgG4-negative group (p= 0.03).		p = 0.02). Among the IgG4-positive group, sclerosing cholangitis was the most common (n=4) followed by sialoadenitis (n=3), retroperitoneal fibrosis, mediastinal lymphadenitis, and lacrimal gland involvement.	response rate was 100% in both IgG4-positive and IgG4-negative pts.	25% in IgG4-positive and -negative group, p = 0.8). The mean interval between CST and relapse was not different between the two groups (14 mo vs. 11 mo in IgG4-positive and -negative groups, p = 0.8).	tapered to a maintenance dose over three mos. MST (5 mg/d) was administered for 6 mo to prevent relapse.	similar between serum IgG4-positive and IgG4-negative type 1 AIP, but other OOI was more common in IgG4-positive than in -negative type 1 AIP. High serum IgG4 was associated with other OOI and tissue IgG4 concentration, but did not affect the relapse rate in type 1 AIP.
**Sánchez Castañón (79) 2015, Spain/Italy**	IgG4 Anti AMY-α 2A	Nephelometry for IgG4 ELISA for anti-AMY-α2A	AIP (n=25: type 1 AIP (n=14); type 2 AIP (n=3); NOS AIP (n=8)). Disease controls (n=84: chronic pancreatitis (n=31); acute pancreatitis (n=30);	ICDC	IgG4 ≥ 135 mg/dL AMY-α 2A: 0.805 x real CO value (3 SD over the mean absorbance value from 16 HC sera).	Increased IgG4 was detected in 52% of the AIP group, 5% of the control group, and 0% of the HC group. Anti-AMY-α2A was found	The mean IgG4 was 323 mg/dL (range, 16–1660 mg/dL). The median IgG4 was 362 mg/dL (range, 56 –1660 mg/dL), 64 mg/dL (range,	N/A	N/A	N/A	N/A	N/A	The present study shows similar Sp but a lower Sn of increased IgG4 for AIP diagnosis (94% and 50%) than previously reported. When the 2 serological markers were combined, Sp

	pancreatic adenocarcinoma (n=23); and HC (n=59).	in 76%, 36%, and 2%, respectively. IgG4 was elevated in 92% of the 13 pts with type 1 AIP, but in none of the 3 pts with type 2 or 8 with NOS. Anti-AMY-α2A was present in 79%, 67%, and 75% of type 1, type 2, and NOS AIP, respectively.	41–87 mg/dL), and 52 mg/dL (16–97 mg/dL) in the sera of the type 1, type 2, and NOS AIP pts, respectively. Sn and Sp of anti-AMY-α2A were 76% and 78%, and of IgG4 50% and 94%. By combining the 2 serological markers, Sn was 41%, and Sp was 99%, with a PPV of 82% and NPV of 92%.		rose to 99% and PPV 82%, although Sn fell to 41%. Anti-AMY-α2A may help to diagnose AIP and to differentiate AIP subtypes.

**Detlefsen (21) 2018, Denmark**	IgG 4, IgG Anti-PBP, anti-CA II, anti-LF, ANA, RF, ANCA	ELISA Nephelometry IIF	AIP (n=29: AIP 1 (n=19); AIP 2 (n=10)), pancreatic cancer (n=17), pancreatic neuroendocrine neoplasm (P-NEN, n=12), and alcoholic CP (n=41).	HISORt	IgG4 ≥ 1.4 g/L (AIP vs. pancreatic cancer/CP) IgG4 ≥ 1.09 g/L (AIP 1vs AIP 2). IgG ≥ 13.5 g/L (AIP vs. pancreatic cancer). Anti-PBP < 38.3 U (AIP vs. pancreatic cancer/CP).	IgG serum ≥ 1.4 g/L was present in 45% AIP pts (58% type 1 AIP pts and 20% type 2 AIP pts), 12% pancreatic cancer pts, and 17% alcoholic CP pts. Serum IgG ≥ 2.80 g/L was present in 28% AIP pts (37% of type 1 AIP pts and 10% of type 2 AIP pts), 6% pancreatic cancer pts, and 5% alcoholic CP pts.	Serum IgG4 was higher in AIP pts (mean 4.2g/L) and especially type 1 AIP pts (mean 6.0g/L) when compared with PC (mean 0.7g/L), P-NEN (mean 0.7g/L) and ACP (0.9 g/L) (p<0.001). There was a lower mean a-PBP value in the AIP group compared with the pancreatic cancer group, but this difference was NS. The Sn and Sp of IgG4 (cutoff 1.4g/L) for	No differences in serum IgG4 level in pts with or without obstructive jaundice.	EPL were found in 12 type 1 AIP pts (63%) but no type 2 AIP (p<0.005). IgG4-related sclerosing cholangitis (IgG4-SC) was most frequent, although not significantly. Two type 1 AIP pts (10.5%) had the involvement of 2 extrapancreatic organs. One type 1 AIP patient had the involvement of 4 extrapancreatic organs, including IgG4-SC, lymphadenopathy, perisplenitis, and lung disease. Eight type 1 AIP pts (42%) and three type 2 AIP pts (30%, p=0.7) had	N/A (the majority of the pts were already on steroids at the time of sera testing)	N/A	N/A	Serum IgG4 was the only serological marker where a significant difference in mean values between AIP and pancreatic cancer was found. However, the value of IgG4 for the distinction of AIP from pancreatic cancer was limited, probably in part due to the relatively high number of type 2 AIP patients in the study. The data from this study do not support a role of increased serum anti-PBP for the diagnosis of AIP. For the differentiation of type 1 and type 2 AIP, the only significant differences

		differentiating AIP from PC was 45% and 88%, but rose to 52% and 88% when using a cutoff of 1.09g/L. When using this cut-off, the Sn and Sp for differentiating type 1 AIP from PC was 68% and 88%. None of the other markers were significantly changed in AIP versus cancer. For the differentiation of type 1 and type 2 AIP, the only significant difference	other autoimmune diseases. Ulcerative colitis was exclusively observed in type 2 AIP (30% vs. 0%, p=0.03).		were IgG4 in type 1 AIP (p<0.01), with a Sn of 68% and a Sp of 80%, and c-ANCA elevations found in some type 2 AIP pts (p < 0.05).

							s were IgG4 in type 1 AIP (p<0.01), with a Sn of 68% and a Sp of 80%, and c-ANCA elevations found in some type 2 AIP pts (p<0.05).						
**Pattabathula (72) 2021, Australia**	IgG4	N/A	AIP (n= 23: type 1 = 13; type 2 = 10).	Confirmed histology, imaging, and serology.	IgG4 >1.40 g/L	IgG4 serum levels were increased in 69% (9/13) type 1 AIP pts but in no (0/10) type 2 AIP pts (p < 0.01).	N/A	N/A	N/A	N/A	N/A	N/A	Pts with type 2 AIP that were younger, had focal disease on cross-sectional imaging and normal IgG4 levels with concurrent IBD underwent more operative interventions than pts with type 1 AIP.

AIP: autoimmune pancreatitis; Sn: sensitivity; Sp: specificity; PPV: positive predictive value; NPV: negative predictive value; EPL: extrapancreatic lesions; IgG4: immunoglobulin G4; IgG: immunoglobulin G; IgE: immunoglobulin E; Eo: eosinophils; pts: patients; DM: diabetes mellitus; CST: corticosteroid treatment; MST: maintenance steroid treatment; N/A: not available; ANA: anti-nuclear antibodies; RF: rheumatoid factor; ICDC: international consensus diagnostic criteria; SD: standard deviation; OOI: other organ

involvement; mo: month; d: day; wk: week; anti-PBP: anti-plasminogen-binding peptide; anti-CA-II: anti-carbonic anhydrase-II; anti-LF: anti-lactoferrin; ANCA: anti-neutrophil cytoplasmic antibodies; ELISA: enzyme-linked immunosorbent assay; IIF: indirect immune fluorescence; P-NEN: pancreatic neuroendocrine neoplasm; CP: chronic pancreatitis; NS: not statistically significant; IgG4-SC: IgG4-related sclerosing cholangitis; anti-AMY-α2A: anti-amylase α antibodies; NOS: not-otherwise-specified AIP by ICDC; PDAC: pancreatic ductal adenocarcinoma; CO: cutoff, SD: standard deviation.

Supplemental Table S5. The role of biomarkers in differentiating between AIP and pancreatic cancer.

**Author, Year, Country**	**Candidate Biomarker**	**Diagnostic method**	**Cohort (n)**	**Marker cutoff value**	**Criteria**	**Mean/Median Sn, Sp, PPV, NPV**	**Frequency of marker elevation in AIP (%) vs. frequency in controls (%)**	**Conclusion**
**Ghazale (30) 2007, USA**	IgG4, CA 19-9	Nephelometry	AIP (n = 45: IgG-positive (n = 34), IgG4-negative (n = 11)), pancreatic cancer (n =135), pts with other pancreatic diseases (n = 268), and pts with no pancreatic disease (n = 62).	IgG4 >140 mg/dL<br>IgG4 >280 mg/dL<br>IgG >1500 mg/dL<br>CA 19-9 > 100 U/mL	HISORt	The mean concentration of serum IgG4 in all pts with AIP was 550 ± 98.6 mg/dL.<br><br>Diagnosis of AIP:<br>IgG4 > 140 mg/dL:<br>Sn 76%, Sp 93%, PPV 36%;<br>IgG4 > 280 mg/dL:<br>Sn 53%, Sp 99%, PPV 75%.<br><br>Total serum IgG had a lower Sn for AIP than serum IgG4 (42% vs. 76%, p = 0.001). Serum IgG4/IgG ratio was also calculated and using a cutoff of 13 (optimal cutoff based on ROC curve data), it had a Sn of 77% (p = 0.8 vs. IgG4 alone) and a Sp of 93%.	IgG4 >140 mg/dL:<br>AIP: 34/45 (76%);<br>normal pancreas: 3/62 (4.8%);<br>pancreatic cancer: 13/135 (9.6%);<br>benign pancreatic tumor: 3/64 (4.7%),<br>acute pancreatitis 5/58 (8.6%);<br>CP: 5/79 (6.3%),<br>miscellaneous: 3/67 (4.5%).<br><br>Serum IgG4 levels were elevated in 13/135 (10%) pancreatic cancer pts; however, only 1% had IgG4 levels >280 mg/dL compared with 53% of AIP. Compared with AIP, pancreatic cancer pts were	Elevated serum IgG4 levels are characteristic of AIP. However, mild (<2-fold) elevations in serum IgG4 are seen in up to 10% of subjects without AIP including pancreatic cancer and cannot be used alone to distinguish AIP from pancreatic cancer. Greater than 2-fold elevations in serum IgG4 are more specific for AIP.

							more likely to have CA19-9 levels of >100 U/mL (71% vs. 9%, p < 0.001).<br><br>Serum IgG4 levels were not different in those with and without obstructive jaundice (mean 586 ± 128 mg/dL vs. 449 ± 88 mg/dL, p = 0.5).	
**Kamisawa (47) 2008, Japan**	IgG4, CA19-9,<br>CEA,<br>Dupan II	N/A	AIP with a mass-like lesion on the pancreas head (n =17) and pancreatic head cancer (n = 70)	IgG4 > 135 mg/dL<br>CA19.9 > 37 U/mL<br>CEA > 5 ng/dL<br>Dupan II >150 U/mL	JPS	AIP vs. pancreatic cancer (mean± SD):<br>IgG4 (mg/dL) 450.2± 486.2 82.1 ± 76.1, p< 0.001;<br>CA19.9,(U/mL)101.4± 143.8 vs. 341.4± 798.0, p<0.001;<br>CEA (ng/dL): 2.8± 1.1 vs. 3.6± 2.8 , p= 0.188;<br>Dupan II (U/mL): 409.1± 650.8 vs. 366.0±584.9, p= 0.127.	AIP vs. pancreatic cancer:<br>IgG4> 135 mg/dL +/- 12/5 vs. 2/3, p < 0.001;<br>CA19.9> 937 U/mL +/- 10/7 vs. 47/23 p = 0.574;<br>CEA> 95 ng/dL +/- 1/16 vs. 20/50 p = 0.060;<br>Dupan II> 9150 U/mL +/- 5/8 vs. 31/21 p = 0.2888.<br><br>Serum IgG4 levels were frequently (p < 0.001) and significantly (p < 0.001) elevated in AIP pts.	When there is at least one positive imaging factor for AIP, serum IgG4 levels should be measured. When serum IgG4 levels are elevated (> 135 mg/dL), in cases with more than 2 positive imaging factors, the indication of CST should be considered under the provisional diagnosis of AIP, whereas in cases with one positive imaging factor, a biopsy guided by US or EUS-FNA should be performed for histological examination.<br>In pts with normal serum IgG4 who have more than 3

								positive imaging factors, an indication of CST should be considered, whereas cases with 1 or 2 positive imaging factors should be biopsied for histological examination. Because AIP responds so readily to CST, a poor response to CST suggests pancreatic cancer and the need for further reexamination, including laparotomy.
**Chari (17) 2009, USA**	IgG4	N/A	AIP (n=48), pancreatic cancer (n=100)	IgG4 >140 mg/dL<br>IgG4 >280 mg/dL<br>CA 19-9 >55 U/L<br>CA 19-9 >150 U/L	HISORt	AIP:<br>IgG4 >140 mg/dL: Sn 81%, Sp 90%<br>IgG4 >280 mg/dL: Sn 50%, Sp 100%<br><br>Pancreatic cancer:<br>CA 19-9 >55 U/L: Sn 77%, Sp 81.3%<br>CA 19-9 >150 U/L: Sn 61.5%, Sp 92.3%.	Pancreatic cancer vs. AIP:<br>IgG4 >140 mg/dL: 10% (10/100) vs. 81.3% (39/48),p < 0.0001;<br>IgG4 >280 mg/dL: 0% (0/100) vs. 50% (24/48), p < 0.0001;<br>CA 19-9 >55 U/L: 76.9% (70/91) vs. 23.1% (9/39), p < 0.0001;<br>CA 19-9 >150 U/L: 61.5% (56/91) vs. 7.7% (3/39) <.0001.	Pancreatic cancer can be distinguished from AIP by pancreatic imaging, the measurement of serum IgG4, and the determination of OOI. However, a pancreatic core biopsy, steroid trial, or surgery are required for diagnosis in approximately 30% of pts with AIP.

**Naitoh (65) 2010, Japan**	IgG, IgG4, CA 19-9, CEA	N/A	Mass-forming AIP (n = 36) and pancreatic cancer (n = 60)	IgG >1800 mg/dL<br>IgG4 >135 mg/dL	JPS<br>HISORt	AIP vs. pancreatic cancer (mean ±SD)<br>IgG4: 777 ± 821 vs. 43 ± 60, p < 0.001;<br>IgG: 2523 ± 1320 vs. 1236 ± 291, p < 0.001<br>CA19-9: 86 ± 143 vs. 1275 ± 3545, p < 0.001;<br>CEA: 2.62 ± 1.38 vs. 4.45 ± 5.79, p = 0.032;<br>IgG4 > 280 mg/dL: 23/30 (77%) vs. 1/60 (2%), p < 0.001.<br><br>The parameters with 100% Sp for AIP were a capsule-like rim during CT, skipped lesions on the main pancreatic duct during ERCP or MRCP, γ-globulin > 2 g/dL, EPL (extrapancreatic biliary stricture, salivary gland swelling and retroperitoneal fibrosis) and EUS-FNA. The findings with over 90% Sp were IgG4 > 280 mg/dL (98%), IgG > 1800 mg/dL (97%), maximal diameter of the upstream main pancreatic duct < 5 mm on MRCP (95%) and IgG4 > 135 mg/dL (94%), respectively.	AIP vs. pancreatic cancer:<br>IgG4 > 135 mg/dL: 26/30 (87%) vs. 4/60 (7%), p< 0.001;<br>IgG > 1800 mg/dL: 21/36 (58%) vs. 2/58 (3%), p < 0.001;<br>IgG4 > 280 mg/dL: 23/30 (77%) vs. 1/60 (2%), p < 0.001;<br>ANA > 80-fold: 20/36 (55%) vs. 10/47 (21%), p=0.003;<br>γ- globulin > 2 g/dL: 18/35 (51%) vs. 0/48 (0%), p< 0.001;<br>CA19-9 > 37 U/mL: 17/33 (52%) vs. 47/60 (78%), p=0.014;<br>CA19-9 > 150 U/mL: 4/33 (12%) vs. 34/60 (57%), p < 0.001;<br>CEA > 5 ng/dL: 3/32 (9%) vs. 19/60 (32%), p= 0.077.<br><br>Increased IgG4 (< 135 mg/dL), IgG (> 1800 mg/dL), γ-globulin (> 2.0 g/dL) and positive rates of ANA were observed more frequently in the pts with mass-forming AIP than in those with pancreatic cancer. On the other hand, increased CA19-9 (> 37 U/mL) and CEA	Clinical, imaging, serological, histological findings and EPL differed between mass-forming AIP and pancreatic cancer. Capsule-like rims during CT, skipped lesions on the main pancreatic duct during ERP or MRCP, IgG4 > 280 mg/dL, and EPL were highly specific findings for AIP. These findings are useful in the differential diagnosis of mass-forming AIP from pancreatic cancer.

							(> 5 ng/mL) were observed more frequently in the pts with pancreatic cancer. CA19-9 and CEA values were higher in the pts with pancreatic cancer than in those with mass-forming AIP.	
**van Toorenenbergen (95) 2010, Netherlands**	IgE<br>IgG4<br>IgE/IgG4 ratio	Fluorescence enzyme immunoassay<br>Nephelometry	AIP (n = 13), pancreatic carcinoma (n = 12), atopic allergy (n =14)	IgG4 > 1.4 g/L	HISORt	AIP (median level):<br>IgG4: 3.7 g/L,<br>IgE: 244 kU/L,<br>IgE/IgG4 ratio: 87 kU/g.<br><br>PDAC (median level):<br>IgG4: 0.62 g/L,<br>IgE: 24 kU/L,<br>IgE/IgG4 ratio: 49 kU/g.<br><br>Both total IgE and total IgG4 levels of the 13 pts with AIP were higher than those in 12 pts with pancreatic carcinoma (p = 0.0004 and p = 0.015, respectively).<br><br>The diagnostic power of serum IgE and IgG4 for discrimination between AIP and pancreatic carcinoma was as follows:<br>IgG4 (1.6 g/L): 69 % Sn and 92% Sp;<br>IgE (136 kU/L): 77% Sn and a 100% Sp;<br>AUC for IgE (0.923) > AUC for IgG4 (0.788). However,	N/A	Analysis of total IgE in serum might be useful in the differentiation between AIP and PDAC. There was a positive correlation between IgG4 and IgE in pts with AIP.

						this was NS probably due to the small sample.		
**Naitoh (64) 2012, Japan**	IgG, IgG4, γ-globulin, ANA, CA 19-9, CEA	N/A	Mass-forming AIP (n = 36) and pancreatic cancer without metastasis (n = 60).	IgG > 1800 mg/d,<br>IgG4 > 135 mg/dL<br>γ- globulin > 2.0 g/dL<br>ANA > 1:80<br>CA 19-9 > 37 U/mL<br>CEA > 5 U/mL.<br>* The rates of serum IgG4 > 280 mg/dL and serum CA19-9 > 150 U/mL were also evaluated.	Revised JPS<br>HISORt	AIP vs. pancreatic cancer (mean ±SD):<br>IgG4 (mg/dL): 777 ± 821vs 43± 60, p < 0.001;<br>IgG (mg/dL): 2523±1320 vs. 1236±291, p < 0.001;<br>CA19-9 (U/mL): 86±143 vs. 1275±3545, p = 0.032;<br>CEA (ng/dL): 2.62±1.38 vs. 4.45±5.79, p < 0.001.<br><br>Findings for diagnosing AIP:<br>IgG4 > 135 mg/dL: Sn 87%;<br>IgG4 > 280 mg/dL: Sn 77%, Sp 98%, accuracy 91%;<br>IgG > 1800 mg/dL: Sn 58%, Sp 97%, accuracy 82%;<br>γ- globulin>2 g/dL: Sp 100%, accuracy 80%.	AIP vs. pancreatic cancer:<br><br>IgG4 (> 135 mg/dL): 26/30 (87%) vs. 4/60 (7%), p < 0.001;<br>IgG (> 1800 mg/dL): 21/36 (58%) vs. 2/58 (3%), p < 0.001;<br>ANA (> 80-fold): 20/36 (55%) vs. 10/47 (21%), p = 0.003;<br>γ -globulin (> 2 g/dL 18/35 (51%) vs. 0/48 (0%), p < 0.001;<br>IgG4 (> 280 mg/dL): 23/30 (77%) vs. 1/60 (2%), p < 0.001;<br>CA19-9 (> 150 U/mL): 4/33 (12%) vs. 34/60 (57%), p < 0.001;<br>CEA (> 5 mg/dL): 3/32 (9%) vs. 19/60 (32%), p = 0.077.	Capsule-like rim during CT, skipped lesion on the main pancreatic duct during ERP or MRCP, IgG4 > 280 mg/dL, and other organ involvement were highly specific findings for AIP. These findings are useful in the differential diagnosis of mass-forming AIP from pancreatic cancer.

**Chang (16) 2014, Taiwan**	IgG4, CA 19-9, CEA	Nephelometry	AIP (n = 188), pancreatic cancer (n = 130), non-AIP CP (n = 86).	Level 1: IgG4 > 140 mg/dL;<br>Level 2: IgG4 > 280 mg/dL.	HISORt Asian ICDC	IgG4 (mean ± SEM):<br>AIP: 346.6 ± 56.2 mg/dL;<br>Pancreatic cancer: 119.2 ± 23.9 mg/dL;<br>Non-AIP CP: 69.5 ± 5.0 mg/dL.<br><br>CA19-9 (mean ± SEM):<br>AIP: 49.8 ± 8.8 U/mL;<br>Pancreatic cancer: 5593.8 ± 987.1 mg/dL,<br>Non-AIP CP: 25.6 ± 23.3 mg/dL.<br><br>CEA (mean ± SEM):<br>AIP: 2.17 ± 4.58 U/mL;<br>Non-AIP: 1.3 ± 1.1 U/mL;<br>Pancreatic cancer: 5.9 ± 20.9 U/mL.<br><br>The optimal cutoffs of IgG4 and CA19-9 to differentiate AIP from pancreatic cancer were 175 mg/dL and 85.0 U/mL based on ROC analysis. Combining IgG4 level > 280 mg/dL and CA19-9 < 85.0 U/mL yielded the best diagnostic accuracy (85.6%).	There were 105 65% (105/162) AIP pts with IgG4 >140 mg/dL. There were 30% (49/162) AIP pts with IgG4 > 280 mg/dL.<br>There were 5% (4/86) non-AIP CP pts with IgG4 > 140 mg/dL and no pts with non-AIP CP with IgG4 > 280 mg/dL. There were 20% (18/90) and 6% (5/90) pancreatic cancer pts with available IgG4 >140 mg/dL and IgG > 280 mg/dL, respectively.	Combined use of serum IgG4 (> 280 mg/dL) and CA19-9 9 (< 85.0 U/mL) together increases the diagnostic accuracy to distinguish AIP from pancreatic cancer non-invasively, especially in focal-type AIP.
**Talar-Wojnarowska (92) 2014, Poland**	IgG, IgG4, anti- CA-II	ELISA	PDAC (n = 45), type 1 AIP (n = 24) and CP (n = 55).	IgG4 >140 mg/dL;<br>IgG >16 g/l<br>Anti- CA-II >31 ng/mL.	ICDC	In AIP pts, the median IgG levels were 19.7 g/L, median IgG4 levels were 301.9 mg/dL, and median anti-CA-II levels were 81.82 ng/mL, compared to 10.6 g/L, 123.2 mg/dL and 28.6 ng/mL, respectively, in PDAC pts. Serum levels of IgG, IgG4 and anti-CA-II were significantly higher in	However, 16 (35.5%) pts with PDAC and 14 (25.4%) pts with CP had IgG4 levels > 140 mg/dL. Moreover, in 3 (6.67%) pts with PDAC, those values were greater than 280 mg/dL. No pts	IgG4 at the cutoff of 210 mg/dL showed the best Sn and Sp in AIP diagnosis compared to IgG and anti-CA-II Ab; however, elevations of serum IgG4 may be seen in subjects without AIP, including PDAC.

						pts with AIP compared to PDAC and CP pts.<br><br>There was no significant difference between IgG, IgG4 and anti-CA-II serum level in PDAC and CP pts. IgG4 for the cutoff 210 mg/dL showed the best Sn and Sp (84% and 90%) in AIP diagnosis compared to IgG (69% and 87%, respectively; cutoff 15 g/L) and anti-CA-II (45% and 74%; cutoff 38.4 ng/mL). AUC was 0.94 (95%CI 0.93–0.97) for IgG4, 0.82 (95%CI 0.8–0.9) for IgG and 0.63 (95%CI 0.60–0.72) for anti-CA-II.	with CP had IgG4 > 280 mg/dL.	Anti-CA-II had the lowest Sn and Sp compared to other analyzed markers, increasing not only in CP pts but also in pts with PDAC. Because high levels of anti-CA-II are frequently seen in subjects with PDAC, anti-CA II cannot be used to distinguish AIP from PDAC.
**van Heerde (94) 2014, Netherlands**	IgG4, CA 19-9	ECLIA Nephelometry	AIP (n = 33), pancreatic carcinoma (n = 53), other pancreato-biliary disorders (n = 145: cholangiocarcinoma (n = 32); CP (n = 52); PSC (n = 30); and SjS (n = 31).	CA 19-9 > 34 U/mL<br>IgG4 > 1.4 g/ L	ICDC Asian HISORt	Median CA 19-9:<br>AIP: 26 U/mL (IQR 12–108);<br>PDAC: 349 U/ mL (IQR 63–1588).<br><br>Median IgG4:<br>AIP: 4.7g/L (IQR 1.8–10.5);<br>PDAC: 0.5 g/L (IQR 0.2–1.1).<br><br>Using an upper level of 74 U/ mL, the assay for CA 19-9 identified pts with AIP with 73 % Sn and 74 % Sp. Using a lower level of 2.6 g/L, the assay for IgG4 identified these pts with 70 % Sn and 100 % Sp.	N/A	Serum IgG4 was significantly higher in pts with AIP than in all other groups. In particular, IgG4 was significantly higher in the AIP group compared to pts with pancreatic carcinoma. Pts with AIP have lower levels of CA 19-9 than those pts with pancreatic carcinoma. Measurements of either the CA 19-9 or the IgG4 level alone are not accurate enough for

						Combining data, low levels of CA 19-9 (< 74 U/mL) and high IgG4 (> 1.0 g/L) identified pts with AIP with 94 % Sn and 100 % Sp.		diagnosis. However, the combination of CA 19-9 < 74 U/mL and IgG4 > 1.0 g/l distinguishes pts with AIP from those pts with pancreatic carcinoma with 94 % Sn and 100 % Sp. Levels of bilirubin did not differ between AIP and malignancy. After adjustment for IgG4 and bilirubin, CA 19-9 remained an independent predictor of AIP against pancreatic carcinoma (OR 0.28; 95 % CI 0.13–0.59).
**Ngwa (68) 2015, USA**	IgG4	Nephelometry	AIP pts (n = 99); pancreatic cancer (n = 548).	IgG4 >121 mg/dL; IgG4 >140 mg /dL.	ICDC	Mean IgG4±SD: Pancreatic cancer: 57±38 g/dL; AIP: 392±183 g/dL.	Pts with AIP were more likely to have any elevation in IgG4 (65/99 (66%)) compared with patients with pancreatic cancer (57/548 (10%), p < 0.001). An elevation of IgG4 more than 2 times ULN was also more common in AIP versus pancreatic cancer (40% vs. 2.4%, p < 0.001).	Mild elevations in IgG4 cannot distinguish AIP from pancreatic cancer. Elevations more than 2 times the upper limit of normal appear more commonly in AIP. Serum IgG4 elevation has no prognostic significance in pancreatic cancer.

**Yan (98) 2017, China**	Globulin, CA 19-9, peripheral Eo count, Hemoglobin	ECLIA	Type 1 AIP (n = 25); pancreatic carcinoma (n = 100).	CA 19-9 > 37 U/mL<br>Eo percentage > 5%<br>Globulin > 35 g/L	ICDC	AIP vs. pancreatic carcinoma:<br>CA 19-9 (U/mL):<br>23.2 (7.6–113.1) vs. 349.8 (24.2–1964.6);<br>Eo%:<br>5.4 (1.8–6.5) vs. 2.0 (1.3–3.0);<br>Hb (g/L):<br>Male (M) 114 (105–131) vs. 133(121–144);<br>Female (F) 107 (94–115) vs. 127 (109–132);<br>Globulin (g/L):<br>34.4(26.1–39.0) vs. 23.1(25.6–28.3).<br><br>The suggested cutoff values for diagnosis of CA19-9 < 306.75 u/mL, Eo% > 4.15%, Globulin > 29.80 g/L and Hb (M) < 114.5 g/L or Hb (F) < 118.5 g/L showed relatively high Sn and Sp (92% and 79%) in AIP diagnosis, with AUC reaching to 0.93 (p < 0.001).	AIP: 44% (11/25) pts had elevated CA 19-9, 52% (13/25) of pts elevated Eo%, (19/25) of pts had decreased Hb 76%, and 48% (12/25) of pts elevated globulin.<br><br>Pancreatic carcinoma: 72% (72/100) pts had elevated CA 19-9, 10% (10/100) of pts had elevated Eo%, 40% (40/100) pts had decreased Hb, and 2% (2/100) of pts had elevated globulin.	Elevated serum Eo and globulin levels together with decreased Hb levels can be used as preoperative indicators for AIP and can help to avoid unnecessary operation.
**Detlefsen (21) 2018, Denmark**	Anti-PBP, anti-CA-II, anti-LF, ANA, RF, c-ANCA, IgG 4, IgG	ELISA, IIF, and nephelometry	AIP (n=29: type 1 AIP (n = 19), type 2 AIP (n=10)); pancreatic cancer (n=17), pancreatic neuroendocrine neoplasm (P–NEN, n=12), and alcoholic CP (n=41).	AIP vs. pancreatic cancer/alcoholic CP:<br>IgG4 ≥ 1.4 g/L<br>anti-PBP < 38.3; U:<br>IgG ≥ 13.5 g/L.<br><br>Type 1 AIP 1 vs type 2 AIP:<br>IgG4 ≥ 1.09 g/L.	HISORt	Serum IgG4 was significantly higher in AIP pts (mean 4.2g/L) and especially type 1 AIP pts (mean 6.0g/L) when compared with pancreatic cancer (mean 0.7g/L), P-NEN (mean 0.7g/L) and alcoholic CP (0.9 g/L). There was a lower mean a-PBP value in the AIP group compared with the	The ANCA analysis was performed in 17 out of 19 type 1 AIP pts and in all type 2 AIP pts. Samples from the type 1 AIP pts were c-ANCA-negative in all cases, whereas 30% of type 2 AIP samples were c-ANCA-positive (p < 0.05).	There was no statistically significant difference in mean a-PBP values in AIP compared with pancreatic cancer. Serum IgG4 was the only serological marker for which a statistically significant difference

						pancreatic cancer group, but this difference was NS.<br><br>AIP vs. pancreatic cancer:<br>a-PBP (cutoff 38 U): Sn 45%, Sp 71%;<br>IgG4 (cutoff 1.4 g/L): Sp 45%, Sn 88%;<br>IgG4 (cutoff 1.9 g/L): Sp 52%, Sn 88%.<br><br>Type 1 AIP vs. pancreatic cancer:<br>IgG4 (cutoff 1.9 g/L): Sp 68%, Sn 88%.<br><br>None of the other markers were significantly changed in AIP versus pancreatic cancer. For the differentiation of type 1 and type 2 AIP, the only differences were IgG4 in type 1 AIP ($p < 0.01$), with a Sn of 68% and a Sp of 80%	45% of AIP, 58% of type 1 AIP, and 12% of pancreatic cancer pts had serum IgG4 values above the cutoff of 1.4g/L. Elevated serum IgG4 levels were seen in all non-AIP patient groups (overall prevalence 7%, range 3–10%) including 9.6% (13/135) pancreatic cancer pts.<br><br>No differences in serum IgG4 level in pts with or without obstructive jaundice.	in mean values between AIP and pancreatic cancer was found. However, the value of IgG4 for the distinction of AIP from pancreatc cancer was limited, probably in part due to the relatively high number of type 2 AIP pts in the study. None of the other markers were significantly changed in AIP versus pancreatic cancer.
**de Vries (20) 2020, Netherlands**	IgG4/IgG RNA Ratio	qPCR	Suspected pancreaticobiliary malignancy (n = 213), Ig4 RD (n = 3)	IgG4/IgG RNA Ratio ≥ 5%	HISORt for IgG4 RD	Median IgG4: 0.48 (IQR, 0.25-0.88) g/L;<br>Median G4/IgG Ratio: 3.9 (IQR 2.0-8.0);<br>Median CA 19-9: 127 (IQR 19-1235) kU/L.<br><br>ROC curve analysis revealed an AUC for an IgG4/IgG RNA ratio of 0.79 to predict IgG4-related disease (Sn 100%; Sp 58.6%). An AUC for CA19-9 of 0.72 to predict	The blood IgG4/IgG RNA ratio was true positive in 3 patients with IgG4-RD (3.3%) and false positive (median IgG4/IgG RNA ratio 9.8%, IQR 6.3-15.2) in 87 patients (40.8%) with other benign or malignant disease. In 123 patients (58.6%) the	The Sn of blood IgG4/IgG RNA ratio was 100%, the Sp 58.6%, and the PPV 3.3%. In the setting of a high a priori risk of malignancy, an elevated IgG4/IgG RNA ratio did not accurately discriminate pancreato-biliary cancer from IgG4-RD as illustrated by

	malignant disease in this cohort (Sn 73.6%; Sp 70.6%) was reached.	test was true negative.	low Sp and concordant low positive predictive value. The authors advise against the use of this test to discriminate IgG4-RD from pancreato-biliary malignancies.

AIP: autoimmune pancreatitis; IgG: immunoglobulin G; IgG4: immunoglobulin G4; IgG4-RD: immunoglobin G4-related disease; IgE: immunoglobulin E; Eo: eosinophils; CEA: carcinoembryonic antigen; CA 19-9: cancer antigen 19-9; Sn: sensitivity; Sp: specificity; PPV: positive predictive value; NPV: negative predictive value; pts: patients; N/A: not available; SEM: standard error of the mean; SD: standard deviation; IQR: interquartile range; CI: confidence interval; qPCR: quantitative polymerase chain reaction; AUC: area under the curve; ROC: receiver operating characteristics; ULN: upper limit of normal; a-PBP: anti-plasminogen-binding peptide; anti-CA-II: anti-carbonic anhydrase-II; ANA: anti-nuclear antibodies; a-LF: anti-lactoferrin; RF: rheumatoid factor; ANCA: anti-neutrophil cytoplasmic antibodies; P-NEN: pancreatic neuroendocrine neoplasm; ELISA: enzyme-linked immunosorbent assay; IIF: indirect immune fluorescence; ECLIA: electro-chemiluminescence immunoassay; JPS: Japan Pancreas Society; ICDC: international consensus diagnostic criteria; PDAC: pancreatic ductal adenocarcinoma; CP: chronic pancreatitis; SjS: Sjögren's syndrome; OOI: other organ involvement; CST: corticosteroid treatment; US: ultrasound; EUS-FNA: endoscopic ultrasonography-guided fine-needle aspiration; CT: computed tomography; ERP: endoscopic retrograde pancreatography; MRCP: magnetic resonance cholangiopancreatography;EPL: extrapancreatic lesions.

Supplemental Table S6. Miscellaneous biomarkers in AIP.

Author Year, Country	Candidate biomarker	Diagnostic method	Cohort (n)	Criteria	Marker cutoff value	Frequency of marker elevation in AIP (%) vs. Frequency in controls (%)	Mean/Median Sn, Sp, PPV, NPV	Steroid treatment response	Conclusion

**Okazaki et al. (71) 2000, Japan**	Antibodies: anti-LF, anti-CA-II, ANA, ASMA, AMA, RF<br><br>Cytokines: IFN γ and IL-4<br><br>Peripheral blood lymphocytes: HLA–DR+CD8+ HLA–DR+CD4+	Antibodies: ELISA and IIF<br><br>Cytokines: FACS (intracellular) and ELISA (extracellular)<br><br>Cells: FACS	Antibodies: AIP (n = 17)<br><br>Cells: 13 of 17 pts with AIP, control pancreatitis (n = 17: 9 gallstone-related and 8 alcoholic pancreatitis), and 17 HC<br><br>Cytokines: Intracellular cytokines in 11 of 17 pts with AIP, 13 pts with control pancreatitis (7 gallstone-related and 6 alcoholic pancreatitis), and 13 HC.	Histology, imaging, serology, steroid trial	N/A	Serum ANA was detected in 76% (13/17) pts, anti-LF Ab in 13 (76%), anti-CA-II Ab in 10 (59%), RF in 5 (29%), and ASMA in 3 (18%), but AMA in none (0%). ALF, ACA-II, ASMA, and AMA were not present in the non-AIP and control groups. ANA was positive in one pt with alcoholic CP.<br><br>The ratios of HLA–DR+CD4+ (17.0%±4.5%) and HLA–DR+CD8+ (25.6%±4.8%) peripheral lymphocytes in AIP pts were significantly higher than those in control pancreatitis pts (HLA–DR+CD4+, 8.5%±1.5%; HLA–DR+CD8+, 10.2%±3.6%) or HC (HLA–DR+CD4+, 8.0%±3.3%; HLA–DR+CD8+, 13.2%±5.6%).<br><br>The ratios of CD4+ cells producing IFN γ in AIP (16.4% ± 5.6%) were significantly higher than those in control pancreatitis pts (7.5% 2.7%) and HC (7.2% ± 2.8%).	Mean serum anti-LF in pts with AIP (1.276± 0.276) was significantly higher than those in pts with gallstone-related pancreatitis (0.513±0.165), pts with alcoholic CP (0.628±0.113), and HC (0.369±0.122).<br><br>Mean serum levels of anti-CA-II in pts with AIP (1.032±0.445) were significantly higher than those in pts with gallstone-related pancreatitis (0.513 ±0.127), pts with alcoholic CP (0.613 ± 0.268), and HC (0.541 ± 0.122). The serum levels of anti-CA-II and anti-LF Ab were not correlated.	N/A	An autoimmune mechanism against CA-II or LF may be involved in AIP. The levels of both intra- and extracellular IFN γ were elevated in AIP pts compared to CP and HC. However, there were no differences in IL-4 levels between groups. This suggests the predominance of the Th 1 immune response in AIP.

						However, the ratios of CD4+ cells producing IL-4 were not different between AIP (2.7% ± 0.8%) and control pts (2.2% ± 0.9%) or HC (1.9% ± 0.7%).			
**Muraki et al. (63)**<br>**2006, Japan**	Complement components: C3, C4 and CH 50<br><br>Mannose-binding lectin (MBL)<br><br>Circulating immune complexes (CIC)	Complement: turbidimetric immunoassay and liposome method<br><br>MBL: ELISA<br><br>CIC: C1q assay and mRF assay	C3, C4, CH 50 analysis: AIP (n = 44) and CP (n = 22)<br><br>MBL analysis: AIP (n = 44), CP (n = 40), HC (n = 48)<br><br>CIC analysis: AIP (n = 44)	Histology, imaging, serology, and steroid treatment	Lower C3: < 86 mg/dL<br><br>Lower C4: < 17 mg/dL<br><br>Lower CH50: < 30 U/mL	AIP pts showed a decrease in complement components: 16 pts showed a decrease in C3 (36%), 16 pts in C4 (36%), and 7 pts in CH50 (17%). Among the controls, 18% (4/22) pts had decreased C3, 14% (3/22) decreased C4, and none decreased CH50.<br><br>Increase in serum CIC levels in AIP pts: 64% (28/44) had elevated CIC measured by the C1q assay, whereas in 73% (32/44) increased CIC was detected by the mRF assay.	Median (range) complement component levels in AIP vs. CP pts:<br>C3: 101 (33–238) mg/dL vs. 102 (66–128); (p = 0.82).<br>C4: 21.1 (5.9–95.6) mg/dL vs. 22.6 (14.2–40.0); (p = 0.49).<br>CH50: 44.3 (10.8–70.7) U/mL vs. 51.3 (33.1–77.3); (p=0.005).<br><br>The median serum MBL values were as follows: AIP, 2139 mg/L; ordinary CP, 4234 mg/L; and HC, 1387 mg/L. The MBL values in cases of both AIP and ordinary CP were significantly higher than those of the HC. There was no significant difference between AIP and ordinary CP in terms of the MBL values.	All treated pts (n = 32) responded favorably to CST, resulting in the improvement of clinical, laboratory, and image findings. MBL values of 32 pts with AIP were compared before and after CST, with no significant difference between these 2 groups. The elevated CIC serum values determined by both assays decreased significantly after CST, suggesting that CIC is associated	AIP exhibits a serum elevation of CIC and abnormalities in the complement activation system. The involvement of the MBL pathway and the possibility of an alternative pathway were excluded and instead suggest that there is an association between high serum CIC values and the classic pathway in the active state of this disease.

								with disease activity.	
**Kawa et al. (52) 2008, Japan**	IgG4 (against IgG1, IgG2, IgG3)<br><br>Novel RF (Ig–Ig interactions)	ELISA	AIP (n = 65), alcoholic or idiopathic CP (n = 111), pancreatic cancer (n = 96), AIH (n = 40), PBC (n = 39), PSC (n = 20), SLE (n = 13), SjS (n = 7), progressive systemic sclerosis (n = 3), and HC (n = 130).	JPS	N/A	Serum IgG4 bound to IgG1 was undetectable (or at very low levels) in 130 HC as well as in pts with other autoimmune diseases (n =119), CP and pancreatic cancer. Patents with AIP had a significantly elevated IgG4.	Median concentrations (range) in AIP pts:<br>IgG1–IgG4: 296 (0–4,185);<br>IgG2– IgG4: 306 (0–3,968);<br>IgG3–IgG4: 142 (0–4,995).<br><br>High serum IgG4 levels able to bind to IgG1 were detected in most samples from the pts with AIP, and the concentrations were significantly higher than those observed in other selected diseases. Assays of IgG4 bound to IgG2 or IgG3 were similar as results of the assays of IgG4 bound to IgG1. Inversely, however, neither IgG1, 2 or 3 showed any NRF activity (unpublished data), i.e., IgG subclasses other than IgG4 did not have the ability to bind to IgG1–4.	N/A	IgG4 auto-Ab interact in vivo with IgG autoantigen in an unprecedented topology. This Fc–Fc interaction departs from the present definition of RF as a Fab–Fc interaction. These data therefore collectively describe a novel RF (NRF) in AIP.

**Taguchi, M. et al. (89) 2009, Japan**	IgA, IgM IgG, IgG4	Nephelometry Turbidimetry	AIP (n = 20), AIH (n = 30), PBC (n = 26), CP (n = 21: alcoholic CP (n = 16), and idiopathic CP (n = 5)) and pancreatic cancer (n = 35)	JPS	IgG/IgM Ratio 15.51<br><br>IgG/IgA Ratio 6.51<br><br>IgG4 ≥ 135 mg/dl	In untreated AIP pts, 25% (5/20) had selective IgM deficiency.	Serum concentrations of IgA in the pts with untreated AIP (212±12 mg/dL) were lower than those of pts with untreated AIH, untreated PBC, CP and pancreatic cancer (391 ± 39, 371 ± 43, 294 ± 24, 314 ± 24, respectively; p<0.05). Serum concentrations of IgM in the pts with untreated AIP (85±11 mg/dL) were significantly lower than those of pts with untreated AIH or PBC (362 ± 59, 527 ± 76, respectively), but not those of pts with CP or pancreatic cancer. Mean IgG was 2,556±262 mg/dL in AIP pts.<br><br>The ratios of IgG/IgM and IgG/IgA in the pts with untreated AIP were significantly higher than those of pts with AIH, PBC, CP and pancreatic cancer. The diagnostic Sn of IgG/IgM and IgG/IgA was 0.80 and 0.95, and the Sp of each ratio was 0.70 and 0.73, respectively, in the differentiation of AIP from the other	Mean IgM was not changed after CST in the pts with AIP, while IgG, IgG4 and IgA significantly decreased. Mean values before vs. after CST:<br>IgG: 2,556±263 mg/dl vs. 1,387± 87 mg/dl (p>0.05);<br>IgA: 213 ±12 mg/dl vs. 183±16 mg/dl (p>0.0.5);<br>IgM: 85 ±11 mg/dl vs. 79 ±15 mg/dl (NS).<br><br>The ratio of serum concentrations of IgG/IgM in the pts with AIP was reduced after glucocorticoid treatment.	Low serum concentrations of IgM and IgA, which have negative correlations with IgG4, are rather specific for AIP. The ratios of serum concentrations of IgG to IgM and of IgG to IgA may serve as novel diagnostic markers in differentiating AIP from other hepatopancreatic diseases.

							diseases. The Sn of IgG4 (cutoff 135 mg/dl) was 0.82 in pts with untreated AIP. In the pts with untreated AIP, the serum concentrations of IgM and IgG or IgG4 were negatively correlated.		
**Takizawa et al. (90) 2009, Japan**	Anti-HSP–10	ELISA	AIP (n = 19), alcoholic CP (n = 24), pancreatic tumor (n = 24), FT1DM (n = 16), acute-onset T1DM (n = 40), T2DM (n = 50), Hashimoto sera (n = 54), HC (n = 71)	JPS	Anti-HSP–10 41.8 (cut-off value for positivity)	Of the pts with AIP (n = 12) who were newly diagnosed but not yet treated with corticosteroid, 92% were positive for HSP–10 auto-Ab. Only 8% (2/24) of pts with alcoholic CP had positive sera, and 8% (2/24) of pts with a pancreatic tumor had positive sera. Interestingly, of the 16 pts in whom FT1DM was newly diagnosed, 81% (13/16) were positive ($p < 0.0001$) for the HSP–10 auto-Ab, with titers nearly comparable to those of pts with AIP.	N/A	When 8 out of 12 pts with AIP were treated with CST, 4 pts (63%) were negative for auto-Ab.	Titers of the auto-Ab were high at onset, and rapidly decreased in response to corticosteroid treatment, suggesting that HSP–10 is a new diagnostic and clinical marker for AIP.
**Matsubayashi et al. (60) 2012, Japan**	Soluble IL-2 Receptor	ELISA	IgG4-positive AIP (n = 29) and IgG4-negative AIP (n = 6)	Asian	IL-2R (range 220–530 U/mL)	N/A	Average levels of serum markers were 1784 mg/dL in IgG, 892 mg/dL in IgG1, 404 mg/dL in IgG4, and 897 U/mL in sIL-2R. Of 6 cases of	Serum IL-2R was high in 20 (77%) of the 26 cases with AIP before steroid therapy, but was high only	The Sn of IL-2R was equal to the current best marker, IgG4 (77%), and it was higher than IgG (54%). In addition,

							sIgG4-negative AIP, 3 cases (50%) were high in sIL-2R serum. The Sn levels of the serum markers for AIP were 54% in IgG, 73% in IgG1, 77% in IgG4, and 77% in IL-2R, (IgG vs. IgG4, IgG vs. sIL-2R; p = 0.08)	in 7 (33%) of the 21 cases after therapy (p = 0.003). IL-2R serum level was significantly reduced after the therapy (t = 4.67, p = 0.0007). A strong correlation was recognized in the levels of serum IL-2R before and after CST (r = 0.72, P =0.001).	serum sIL-2R was significantly reduced, paralleling with CST, suggesting the possible role of IL-2R as an indicator of the need for maintenance therapy. Together with the strong correlation between serum IL-2R and the number of systemic lesions associated with AIP, IL-2R is proposed to be a potent serological marker for assessing the autoimmune activity of AIP.
**Du et al. (22) 2015, China**	Anti-prohibitin antibody	ELISA	IgG4–RD (n = 89: AIP (n = 34), Mikulicz's disease (n = 15), retroperitoneal fibrosis (n = 11), other probable IgG4–RD (n = 29)), SjS (n = 30), and HC (n = 70).	Japanese for IgG4-RD	The critical point for positive definition was a number with a higher value than that of the HC (mean + 3 SD).	Anti-PHB Ab were present in the sera of patients with definite autoimmune pancreatitis (25/34; 73.5%), Mikulicz's disease (8/15; 53.3%), retroperitoneal fibrosis (6/11; 54.5%), other probable IgG4–RD (26/29; 89.7%), and SjS (4/30; 13.3%), but not in HC (1/70; 1.4%). Of the 89 sera with IgG4–RD analyzed, 65 (73%)	N/A	N/A	One positive autoantigen was identified as PHB. ELISA analysis showed that a majority of pts with IgG4–RD have Ab against PHB. Anti-PHB Ab were present in the sera of pts with definite autoimmune pancreatitis, Mikulicz's

						were found to be reactive with PHB, but only 1 (1.4%) of the 70 HC samples was positive.			disease, retroperitoneal fibrosis, and other probable IgG4–RD and SjS, but not in HC.
**Hamada et al. (31) 2015, Japan**	miR-150-5p<br>miR-30-3p	Microarray<br>RT PCR<br>ingenuity pathway analysis	Microarray analysis:<br>AIP type 1 (n = 3, each before and after CST), CP (n = 5), pancreatic cancer (n = 5) or HC (n = 5).<br><br>RT PCR (the expression of miR-150-5p and miR-30-3p):<br>AIP, CP, PC and HC (n = 10 each)	ICDC	N/A	Microarray analysis identified miRNAs highly expressed in the serum of pts with AIP: 13 miRNAs vs. CP; 204 miRNAs vs. pancreatic cancer; and 19 miRNAs vs. HC. miR-150-5p was commonly upregulated in AIP compared to the other samples miR-30b-3p had a low expression level in the RT PCR.	N/A	No miRNA was identified whose expression was decreased to less than 1/3 after CST. The expression level of miR-150-5p did not change significantly after CST.	The expression level of miR-150-5p was significantly higher in AIP pts than in CP pts, pancreatic cancer pts and HC, suggesting that miR-150-5p might be a novel serum marker of AIP.
**Akamatsu et al. (11) 2016, Japan**	miR-7<br>miR-34a<br>miR-181d<br>miR-193b	RT PCR	PDAC (n = 69), IPMN (n = 26), AIP (n = 15)	Histology, clinical course, imaging	N/A	The PDAC and IPMN groups had higher amounts of all miRNAs than the AIP group. The fold change in the median value for miR-7 was 1.8 in the PDAC group (p = 0.009) and 2.4 in the IPMN group (p<0.001) relative to that in the AIP group. The corresponding value for miR-34a was 3.7 in the PDAC group (p<0.002) and 3.3 in the IPMN group (p<0.001). The corresponding values for miR-181d and miR-193b were 3.5	Distinguishing PDAC from AIP:<br><br>miR-7: AUC 0.72 (95% CI = 0.60–0.84), 72% Sn and 73% Sp.<br><br>miR-34a: AUC 0.84 (95% CI = 0.75–0.94), 81% Sn and 80% Sp.<br><br>miR-181d: AUC 0.89 (95% CI = 0.81–0.95), 81% Sn and 80% Sp.<br><br>miR-193b: AUC 0.80 (95% CI = 0.72 to 0.89), 79% Sn and 73% Sp.	N/A	A significant difference in miRNA expression was demonstrated between AIP (a non-neoplastic disease) and PDAC and IPMN (both neoplastic). Abnormality of MAPK-associated miRNAs in serum may have potential as novel noninvasive biomarkers for differentiating PDAC from AIP.

						(p<0.001), 5.1 (p<0.001), 4.1 (p<0.002), and 2.5 (p<0.001), respectively. There were no significant differences in these values between PDAC and IPMN.			
**Felix et al. (25) 2016, Germany**	Antibodies against CA I, CA II, PRSS1, enolase– 1, p–LIP, transferrin, annexin 2, transaldolase	ESI–mass spectrometry, Western blot, ELISA	Serum auto-Ab reactivity analysis: AIP (type 1 AIP (n = 3), type 2 AIP (n = 2));<br><br>Antibody concentration analysis:<br>AIP (type 1 AIP (n = 11), type 2 AIP (n = 9)), PDAC (n = 20 samples) and HC (n = 20).	HISORt	Cut-off of positive ELISA result was defined as value of control mean plus 2 SDs.	N/A	Mean values of auto-Ab (anti-p–LIP, anti-transferrin, anti-enolase 1, anti-PRSS–1) were significantly higher in pts with AIP than in PDAC. Levels of anti-CA–I, anti-CA–II, anti-anexin–2 were significantly higher in AIP than in PDAC grop. Mean levels of anti-TALDO levels were lower in type 1 AIP compared to type 2 AIP pts (1079±857 ng/mL vs. 4016±2568 ng/mL, p<0.01). Mean value of Anti-p–LIP in type 1 AIP was higher compared to type 2 AIP (1197± 885 vs. 381.2±369.4, p=0.0057).	N/A	The development of auto-Ab profiling arrays combined in a single multiplex test for the clinical routine able to screen patients' sera for elevated titers might be a valuable tool to assist diagnosis and help clinicians to recognize AIP. Particularly, antitransaldolase and anti-pancreatic lipase with higher titers in AIP type 1 are promising auto-Ab tools to distinguish type 1 AIP from type 2 AIP.

**Tomoda et al. (93) 2016, Japan**	N-glycan profile<br><br>(Glycans #3410, #3510, and #4510)	Glycoblotting	AIP type 1 pts before treatment with steroids (n = 21), disease controls (n = 205: PDAC (n = 86), CP (n = 40) and IPMN (n = 79)), HC (n = 60).	ICDC	IgG4 ≥ 210 mg/dL (2-fold higher than the upper level of normal)<br><br>IgG ≥ 1667 mg/dL	Among the 53 glycans examined, the expression of 14 glycans differed significantly between AIP patients and HC. All of these glycans, with the exception of glycan #6600, were upregulated in patients with AIP. However, glycans #3410, #3510, and #4510 showed the highest utility for discriminating AIP from HC.<br><br>In AIP pts, the median IgG4 level was 198 mg/dL (range, 109–362), and 52.4% (11/21) pts were categorized as having low IgG4 with the median IgG4 level of 198 mg/dL (range, 109–362).	The median concentrations of glycan #3510 were 7.6, 6.0, and 2.1 mM, and those of #4510 were 6.5, 8.0, and 3.4 mM in AIP pts with high IgG4, AIP pts with low IgG4, and in HC, respectively. For discriminating AIP from HC, glycans #3410, #3510, and #4510 showed high AUC values (0.96, 0.96, and 0.97, respectively).<br><br>The expressions of the three glycans in AIP were significantly higher than those in pancreatic cancer, CP and IPMN ($p < 0.0001$). The median values of glycan #3410 were 28.9, 11.8, 11.5 and 10.5 mM, the values of glycan #3510 were 6.2, 2.5, 2.5 and 4.2 mM, and those of #4510 were 7.6, 3.8, 3.9 and 4.7 mM in AIP, pancreatic cancer, CP and IPMN, respectively.	Sixteen AIP pts (76.2%) were administered CST and all pts responded well to the treatment. Changes in glycans by CST were tested in 6 pts. The glycan expressions were decreased by the treatment in most of the cases. Especially glycan #3410 was decreased in all cases.	Whole-serum N-glycan profiles are altered in AIP patients, and the particular glycans #3410, #3510, and #4510 were significantly elevated in AIP, indicating that the glycans might be valuable biomarkers of AIP. The expression level of #3410 was high in patients with high serum IgG, but other factors, such as the form of pancreatic enlargement, the presence of EPL, and serum IgG4 levels were not correlated with the expression levels of the three glycans.
**Ghassem–Zadeh et al. (29)**	IL-1β<br>IL-7	Serum and tissue	Sera: AIP (n = 29: type 1 AIP (n = 14) and type 2 AIP (n	HISORt Postoperative histology	Cutoff in regard to	N/A	Serum cytokines: Significantly higher concentrations of IL-7	N/A	The cytokines IL-1β, IL-7, and G–CSF can be

**2017, Germany**	IL-13<br>G–CSF	cytokine multiplexing	= 15)), CP (n = 17), and PDAC (n = 27).<br><br>Pancreatic tissue: AIP (n = 12: type 1 AIP (n = 6) and type 2 AIP (n = 6), CP (n = 12), and PDAC (n = 12).	ROC curve:<br><br>IL-7 10.02 pg/ml<br><br>G–CSF 9.92 pg/ml	(p = 0.001), IL-13 (p = 0.02), and G–CSF (p = 0.04) were found in type 1 AIP compared to PDAC.<br>Significantly higher levels of IL-1β (p = 0.02), IL-7 (p = 0.005), and G–CSF (p = 0.03) were found in type 2 AIP compared to PDAC.<br>Comparing type 2 AIP with CP, significantly higher levels were found for IL–6 (p = 0.04), for IL–17 (p = 0.04), and G–CSF (p = 0.003) in type 2 AIP.<br><br>Tissue cytokines: Both AIP subtypes were distinguished from another by two cytokines IL–8 (p = 0.02) and IFN–γ (p = 0.04) showing higher levels in the AIP–2 subtype. IL–10 also showed slightly higher levels in AIP–2 (p = 0.05).<br><br>IL–7 discriminated better than G–CSF AIP from PDAC (AUC of 0.78 vs. 0.69). Combination of both IL–7 and G–CSF for differentiation only	routinely measured in patients’ serum, providing an elegant and non-invasive approach for differential diagnosis. G–CSF is a good candidate to supplement the currently known serum markers in predictive tests for AIP and represents a basis for a combined blood test to differentiate AIP and particularly type 2 AIP from PDAC, enhancing the possibility of appropriate treatment.

							marginally improved the diagnostic value of the two markers (AUC = 0.78); addition of IL–1β showed no further improvement.		
**Kobayashi et al. (53) 2018, Japan**	Serum apolipoprotein A2 isoforms	ELISA	Type 1 AIP (n = 32)<br>HC (n = 36)	JPS 2011	IgG4 >135 mg/dL	IgG4 was elevated in 87.5% of AIP pts.	The serum level of homodimer apoA2–ATQ/ATQ in AIP pts was higher than in HC (52.3 mg/mL vs. 31.9 mg/mL, p < 0.0068). On the other hand, AIP pts displayed lower apoA2–AT levels than HC (27.5 mg/mL vs. 88.2 mg/mL, p <0.0001). The serum levels of heterodimer apoA2–ATQ/AT calculated based on the ELISA results were lower in the AIP pts than in HC (28.9 mg/mL vs. 45.5 mg/mL, p= 0.0024).	N/A	Reduced levels of heterodimer apoA2–ATQ/AT and a specific apoA2 isoform hypo-processing pattern in the sera of AIP pts were detected. This specific serum apoA2 isoform distribution pattern might facilitate the differential diagnosis of pancreatic diseases, including AIP and pancreatic cancer.
**Shiokawa et al. (83) 2018, Japan**	Anti-laminin 511– FL<br><br>Anti-laminin 511–E8 (truncated form)	ELISA	Training cohort: AIP (n = 10); HC (n = 10)<br><br>Validation cohort: AIP (n = 41); controls (n = 92); cancer, autoimmunity, or other diseases and HC (n = 20)).	ICDC<br>IgG4–RD	Optical density units (OD) of the mean + 3 SDs of the control sera	Validation cohort: IgG Ab against laminin 511–E8 were detected in 49% (20/41) AIP pts but in only 2% (2/112) of controls (p < 0.001) When the training and validation groups were combined, IgG Ab against laminin 511–E8 were present in 51% (26/51) AIP pts and in 2% (2/122) of the	N/A	Serum samples of five pts with laminin 511–E8 Ab before and after CST were compared. The laminin 511–E8 Ab titer decreased to under the cutoff value in	A truncated form of laminin 511 that retains full capability for binding to integrins a6b1 and a3b1 is a target antigen in pts with AIP. AIP patients with laminin 511–E8 Ab exhibited distinctive clinical

						controls ($p < 0.001$). Only one AIP patient had anti-laminin 511–FL (full-length) Ab.		association with a decrease in serum IgG4 concentration and the improvement of the pancreatic image.	features, as the frequencies of malignancies or allergic diseases were significantly lower in patients with laminin 511–E8 Ab than in those without. The auto-Ab against integrin α6β1 (a ligand for laminin 511) were found in 4 of 25 AIP pts who were negative for the laminin 511–E8 Ab.
**Ito et al. (43) 2020, Japan**	IL-35	Bio-Plex Pro Human Inflammatory Panel	AIP type 1 (n = 32); alcoholic CP (n = 16); and HC (n = 22)	ICDC	N/A	N/A	IL-35 levels in pts with AIP1 were higher than in ACP and in HC (260.97± 66.17, 219.57 ± 34.56, and 170.72 ±46.94 pg/mL, respectively). IL-29 levels were significantly higher in type 1 AIP than in alcoholic CP and HC (29.18 ± 9.893, 22.09 ±9.364, and 19.51± 8.051 pg/mL, respectively). IL-29 was positively correlated with IL-35. However, IL-35 and IL-29 did not correlate with the serum IgG4 levels of the pts with AIP. There was no	N/A	This study identified elevated expression of plasma IL-35 and tissue IL-35 subunits in pts with type 1 AIP. This might lead to inflammation suppression via activated Tregs. IL-35 might be associated with this anti-inflammatory role, especially against the Th2 response through several cytokines and the differentiation of Tregs in type 1 AIP.

							significant difference between IL-28A levels in type 1 AIP and alcoholic CP (IL-28A: type 1 AIP, ACP, and HC; 25.81±7.108, 24.15 ±4.428, and 14.52 ± 7.363 pg/mL, respectively; p<0.001 (type 1 AIP vs. HC), p<0.001 (ACP vs. HC)).		
**Minaga et al. (62) 2020, Japan**	INF-α IL-33	ELISA	Pts with type 1 AIP 1 and/or IgG4–RD (AIP only) (n = 7); pts with AIP + IgG4–RD (n = 12); IgG4–RD alone (n = 2)); definite alcoholic CP (n = 12); and HC (n = 8)	JPS 2011 IgG4–RD	Cutoff in regard to ROC curve: IFN-α 55 pg/mL IL-33 274 pg/mL IgG1 1054 mg/dL IgG4 182 mg/dL	N/A	Serum IFN-α concentration-discriminated type 1 AIP/IgG4–RD pts from CP pts with an AUC value of 0.93, a Sn of 86%, and a Sp of 92%. The AUC of IFN-α (0.93) was higher than that of IL-33 (0.91), IgG1 (0.79), or IgG4 (0.91). No significant difference was observed in the AUC between IgG4 and IFN-α or IL-33. The parameters obtained by ROC curve analysis showed that the serum levels of IFN-α and IL-33 may be useful as diagnostic biomarkers for type 1 AIP/IgG4–RD (in addition to IgG4).	Twelve pts with type 1 AIP/IgG4–RD were treated with prednisolone and entered remission. The induction of remission by CST markedly decreased the serum concentration of IFN-α and IL-33.	Serum IFN-α and IL-33 concentrations in pts who met the diagnostic criteria for definite type 1 AIP and/or IgG4–RD were significantly higher than in those with CP or HC. Strong correlations between serum IFN-α, IL-33, and IgG4 concentrations were observed. Diagnostic performance of serum IFN-α and IL-33 concentrations as markers of type 1 AIP and/or IgG4–RD was comparable to that of serum IgG4 concentration, as

									calculated by the receiver operating characteristic curve analysis.
**Nakamaru et al. (66) 2020, Japan**	miR-21e5p	Microarray, RT PCR, in situ hybridization	Microarray analysis: AIP type 1 (n = 10), CP (n = 10) and HC (n = 10).<br><br>RT PCR: AIP type 1 (n = 14), CP (n = 10) and HC (n = 10).<br><br>In situ hybridization: AIP type 1 (n = 3), CP (n = 3) and HC (n = 3).	JPS	N/A	miR-21e5p was more up-regulated in AIP than in HC (p = 0.035) and CP (p = 0.048). The number of miR-21e5p-positive inflammatory cells was significantly elevated in AIP compared in CP (p=0.014).	There were no positive correlations between miR-21e5p and clinical data on WBC, IgG, IgG4, IgE, CRP, P–Amy, CEA, and CA19–9.	N/A	miR–21e5p was highly expressed in pancreatic inflammatory cells in AIP when compared with those in HC and CP. miR-21e5p may be involved in the regulation of effector pathways in the pathophysiology of AIP, thus differentiating AIP from CP.
**Ikemune et al. (40) 2021, Japan**	Serum-free light chains (FLC)	Nephelometry	AIP type 1 (n = 37), alcoholic CP (n = 17), 21 idiopathic CP (n = 21), pancreatic cancer (n = 20) and HC (n = 21)	ICDC	κ–FLC (range: 3.3–19.4 mg/L)<br><br>λ–FLC (range: 5.7–26.3 mg/L)<br><br>κ/λ ratio 1.32<br><br>IgG4 ≥ 1350 mg/L	N/A	Active AIP pts have significantly higher serum levels of κ–FLC (median 30.97 (12.3–227.0) mg/L) and λ–FLC (median 20.53 (12.36–102.7) mg/L) than HC (κ–FLC; median 12.5 (3.1–52.1) mg/L), and λ–FLC: median 12.45 (5.4–39.5) mg/L; $p < 0.05$) correlating with raised serum IgG4 and significantly higher summated FLCs (median 53.09 (25.0–218.0) mg/L) than ICP pts (median 26.77 (15.0–89.2)	Pts in remission displayed lower levels of serum IgG4 than those with active disease (6 of 7 AIP pts in remission displayed normal serum IgG4 (median 1032.29 mg/L, range 482.0–3418.7 mg/L).<br><br>Pts in remission displayed	Pts with type 1 AIP have increased serum κ– and λ– FLC concentrations, summated with FLC and k/λ ratios. These novel biomarkers may be useful in the diagnosis of type 1 AIP and in monitoring disease activity.

	mg/L) and HC (median 24.43 (8.5–91.6) mg/L; $p < 0.05$). AIP pts (median 1.43 (0.84–3.24)) showed significantly higher κ/λ ratios than ACP (median 0.83 (0.42–1.18)), ICP (median 0.87 (0.47–2.16)), pancreatic cancer (median 0.90 (0.48–1.27)) and HC (median 0.963 (0.51–1.32)).<br>At the κ/ λ ratio cut-off of 1.32, as established by the ROC curve, the Sn and Sp were 54.1% and 96.2%, respectively. The Sn, Sp, PPV and NPV were highest for IgG4 when compared with κ and λ FLCs, FLC P and the κ/λ ratio, confirming the clinical use of serum values of IgG4 in aiding in the diagnosis of IgG4–RD.	lower levels of κ–FLC (median 16.5 mg/l , range 9.0–25.0 mg/l) and λ–FLC (median 13.9 mg/L , range 8.4–23l9 mg/L) than those with active disease.	

AIP: autoimmune pancreatitis; Sn: sensitivity; Sp: specificity; PPV: positive predictive value; NPV: negative predictive value; miR/miRNA: microRNA; RT PCR: real-time polymerase chain reaction; CP: chronic pancreatitis; HC: healthy controls; ICDC: international consensus diagnostic criteria; N/A: not available; PDAC: pancreatic ductal adenocarcinoma; IPMN: intraductal papillary mucinous neoplasm; AUC: area under the curve; CI: confidence interval; MAPK: mitogen-activated protein kinase; JPS:

Japan Pancreas Society; WBC: white blood cell; IgG: immunoglobulin G; IgG4: immunoglobulin G4; CA19-9: carbohydrate antigen 19-9; CEA: carcinoembryonic antigen; CRP: C-reactive protein; IgA: immunoglobulin A; IgM: immunoglobulin M; AIH: autoimmune hepatitis; PBC: primary biliary cirrhosis; CST: corticosteroid treatment; NS: not statistically significant; ELISA: enzyme-linked immunosorbent assay; IgG4-RD: IgG4-related disease; SD: standard deviation; SjS: Sjögren's syndrome; PHB: human prohibitin protein; OD: odds ratio; CA-II: carbonic anhydrase II; CA-I: carbonic anhydrase I; PRSS1: Serine Protease 1; ESI–mass spectrometry: electrospray ionization tandem mass spectrometry; TALDO: transaldolase; ROC: receiver operating characteristics; FT1DM: fulminant type 1 diabetes; T2DM: type 2 diabetes mellitus; HSP 10: heat shock protein 10; IL: interleukin; Th: T helper cells; Tregs: regulatory T cells; Bio-Plex Pro Human ALF: antilactoferrin; LF: lactoferrin; ACA-II: carbonic anhydrase II antibody; ANA: anti-nuclear antibodies; AMA: anti-mitochondrial antibodies; RF: rheumatoid factor; ASMAs: anti-smooth muscle antibodies; IFN: interferon; HLA: human leukocyte antigen; IF: indirect immune fluorescence; G-CSF: granulocyte colony-stimulating factor.

Supplemental Table S7. Role of serum biomarkers in CST monitoring and the prediction of relapse.

**Author Year, Country**	**Candidate biomarker**	**Cohort (n)**	**Criteria**	**Cutoff**	**Mean (±SD)/Median (range) Sp, Sn, PPV, NPV**	**Steroid treatment response**	**Relapse**	**Steroid regimen**	**Conclusion**
**Hamano (3) 2001, Japan**	IgG4, IgG, IgE, CIC	Sclerosing pancreatitis (n = 20), HC (n = 20), pancreatic cancer (n = 70), ordinary CP (n = 45), PBC (n = 20), PSC (n = 8) or SjS (n = 11).	Histology, imaging, serology and steroid response	IgG4 > 135 mg/dL IgG > 1883 mg/dL	Before vs. after treatment: IgG: 2389 (1349-4310) vs. 1138 (604-1573); p = 0.002. IgG4: 742 (265, 1150) vs. 223 (37, 433); p = 0.002. IgG4/IgG ratio: 0.28 (0.18, 0.50) vs. 0.18 (0.03, 0.34); p = 0.02. CIC: 30 (2, 58) vs. 3 (2, 10); p = 0.003. Serum IgG4 subclass of immune complexes: 1.0 (0.1, 1.7) vs. 0.2 (0.1, 0.5); p = 0.002.	Initial CST-induced clinical remission was seen in all (n = 20) sclerosing pancreatitis pts. The tested sera (n = 12) also showed a significant decrease in IgG4, IgG4/IgG ratio, CIC, and the IgG4 CIC subclass.	During the follow-up 10 to 63 mos after treatment discontinuation, two pts had recurrences. No data on relapse in relation to biomarker level.	Initial: 40 mg/d of oral PSL for 4 wks; Tapering: by 5 mg/wk over 7 wks until reaching 5 mg/d.	Pts with sclerosing pancreatitis have high serum IgG4 concentrations and the values are closely associated with disease activity.

**Hirano (37) Japan, 2007**	IgG4	AIP (n = 42: without CST at onset (n = 23) and with CST at onset (n = 19))	HISORt JPS	IgG4 ≥ 135 mg/dL	Event occurrence vs. event-free group: Mean IgG4 in pts with CST: 397±230 vs. 607±386; p = 0.222. Mean IgG4 in pts without CST: 545±683 vs. 543±226; p = 0.993.	Four wks after CST all pts achieved clinical and laboratory improvement. No significant difference was noted between the event occurrence and event-free groups in terms IgG4, initial dose of PSL, and cessation of CST.	Event occurrence vs. event-free group:<br><br>Mean IgG4 in pts with CST: 397±230 vs. 607±386; p = 0.222.<br><br>Mean IgG4 in pts without CST: 545±683 vs. 543±226; p = 0.993.	Initial: 25–50 mg/d of PSL for 2–4 wks. Tapering: by 5 mg every 2–4 wks until reaching 5 mg/d; MST: 2.5–5 mg/d.	Serum IgG4 levels at onset showed no correlation with prognosis. Although changes in IgG4 levels may give clinically useful information, singly tested IgG4 levels are not likely to be sufficiently informative for the prediction of severity or prognosis.
**Frulloni (27) 2009, Italy**	IgG4<br>CA 19-9	AIP (n=87: focal type (n=55); diffuse type (n=32))	Histology, imaging, serology, OOI, response to steroids	IgG4 > 135 mg/dL<br>CA 19-9 > 25U/I	The mean serum levels of IgG4 were higher in focal than in diffuse AIP (267.5±332 mg/dL vs. 78.2±65.8 mg/dL; p= 0.009). The highest observed value of CA 19-9 was 332 U/L.	90% (60/67) of AIP pts were treated with steroids, with no differences between focal and diffuse forms. All patients responded to CST.	Recurrences were observed in 25% (22/87) of cases, and were more frequent in focal AIP than in diffuse AIP (33% (18/55) vs. 12 % (4/32); p = 0.043), in smokers than in non-smokers (41% (14/34) vs. 15% (8/53); p = 0.011), and in patients with pathological serum levels of IgG4 compared to those with normal serum levels (50% (14/28) vs. 12% (4/28); p = 0.009). The OR to develop recurrences was 3.9 (CI 1.4 – 10.9) for smoking (no / yes) and 6 (CI 1.6 – 21.8) for IgG4 (< 135	Initial dose: 1 mg/kg of body weight/day for 2 – 3 wks.<br><br>Tapering: by 5 mg every wk up to suspension.	Focal-type and diffuse-type AIP differ regarding clinical symptoms and signs. Recurrences occur more frequently in focal AIP than in diffuse AIP. Disease recurrence is more frequent in older pts suffering from focal AIP, especially if they are smokers and in the presence of high serum levels of IgG4.

							mg / dl vs. >135 mg / dl).		
**Sandanayake (81) 2009, United Kingdom**	IgG, IgG4	AIP (n = 28), with 82% (23/28) having AIC/AIP	HISORt JPS 2006	IgG4 ≥ 1.3 g/L	Before treatment: Raised IgG4 was found in 68% (17/25) subjects with a median IgG4 of 2.06 g/L.<br>After treatment: 78% (12/15) pts had raised IgG4 with a median of 0.81 g/L in those who achieved and maintained remission.<br>The median serum IgG4 of the combined relapse/failure to wean the steroid group after initial treatment was 1.35 g/L. The difference between these groups was NS.	All 28 pts exhibited a disease response within 4-6 wks of starting CST. Steroids were reduced and stopped after disease remission in 23/28 (82%) pts after s median of 5 mos of therapy. Five pts failed the weaning of their initial steroid course.	35% (8/23) pts relapsed; 22% (5/23) pts failed to wean.	Initial: oral 30 mg/d of PSL with an individual reducing regimen;<br>Tapering: by 5 mg every 2 wks after the first 2 wks, with steroid cessation after 3-4 mos.	There was NS correlation between serum IgG4 levels and relapse. Further studies will be necessary before the monitoring of serum IgG4 levels can be used in clinical practice to tailor therapy, and there is insufficient evidence to support the continuation or reintroduction of immunosuppression on the basis of serum IgG4 alone. The presence of EPL, in particular IAC, predicted a lack of maintained disease control after an initial steroid course.
**Hirano (34) 2010, Japan**	IgE, IgG4, IgG	AIP (n = 67)	HISORt JPS	IgE > 170 IU/mL<br>IgG4 ≥ 135 mg/dL	The average value of IgE was 679±675 IU/mL (range, 67-3000 IU/mL).<br><br>Differences in mean before vs. after CST:<br>IgE (IU/mL): 723±744 vs. 673±660; p = 0.63.<br>IgG (mg/dL): 1891±566 vs. 1155±315; p < 0.0001.<br>IgG4 (mg/dL):	IgE measured before CST and during MST could be compared in 29 pts. IgE increased in 10, and decreased in 18 pts. High IgE was observed in 90% (26/29) before CST, and in 79% (23/29) after CST (p = 0.470). By	There was no difference in IgE in the pts with and without clinical relapse (457±346 IU/mL vs. 784±786 IU/mL, p = 0.374). Clinical relapse was observed in 5 of 33 pts. Their relapses occurred 16 mo after the initiation of steroid therapy, on	Initial: oral 30-40 mg/d of PSL for 2-4 wks;<br>Tapering: by 5 mg every 2-6 wks until 10 mg/d;<br>MST: 2.5-7.5 mg/d.	There were NS correlations between IgG, IgG4, and IgE. There was NS difference in IgE in the patients with and without EPL, with and without clinical relapse, and between the before and after CST groups. Although IgE does not

					557±429 vs. 229±112; p = 0.0002.<br><br>EPL were observed in 20 pts (48%). There was NS difference in IgE in the pts with and without EPL (526±531 IU/mL vs. 819±768 IU/mL, p = 0.163).	contrast, IgG and IgG4 did show significant differences before and after CST.	average (range, 3-26 mo). The mean follow-up period was the same between the pts with and without clinical relapse (53.0 mo vs. 51.8 mo, p = 0.929). Neither IgE, IgG nor IgG4, were related to later clinical relapses.		necessarily reflect the disease activity, IgE might be useful for the diagnosis of AIP in the inactive stage.
**Hirano (35) 2011, Japan**	IgG, IgG4	AIP treated with CST (n = 49): without previous aggravation (n = 28);<br>with previous aggravation (n = 21); relapse during maintenance (n = 9); relapse during follow-up with no CST (n = 12)).	Asian	IgG4 ≥ 135 mg/dL<br>IgG > 1800 mg/dL	At initial measurement, mean IgG was 1970±703 mg/dL and mean IgG4 was 530±482 mg/dL. No difference in mean IgG and IgG4 at the initial measurement between the pts with (n = 17) and without (n = 28) a history of relapse (p = 0.16).<br>To differentiate the aggravation phase from the remission phase: IgG (cut-off 1600 mg/dL) had a Sn of 77%, Sp of 94%, and accuracy of 85%; IgG4 (cut-off 244 mg/dL) had 81% Sn, 67% Sp, and 74% accuracy.	After remission, IgG was normal in 96% of pts (n = 46), whereas IgG4 was normal only in 31% pts (n = 13).	The average value of IgG before aggravation was significantly smaller than that at aggravation. No such significance was observed for IgG4 (n = 8). The average values of IgG and IgG4 at aggravation were significantly larger than that after remission.	Initial: oral 30-40 mg/d of PSL for 2-4 wks;<br>Tapering: gradually until 10 mg/d;<br>MST: 2.5-7.5 mg/d.	IgG and IgG4 reflect the disease activity of AIP. By measuring them during follow-up, it may become possible to predict future clinical relapse in a considerable proportion of the pts. Adjusting steroid dose so that the elevation of IgG and IgG4 may not occur might be useful for the prevention of clinical relapse.
**Kubota (54) 2011, Japan**	IgG4, IgG	Type 1 AIP (n = 70)	Asian	IgG4 ≥135 mg/dL<br>IgG ≥ 1800 mg/dL	IgG elevated in 60% pts (n = 70), IgG4 elevated in 75% pts (n = 64).	The median length of follow-up was 46.9 mos (range 6–120 mos). All of the 42 pts who received CST	Relapse was recognized in 34.3% (24/70) of pts overall, all of whom showed good response to repeat CST. Of the	Initial: 30–40 mg/d PSL;<br>Tapering: by 5 mg each wk until a daily dose of 5 mg was reached;	AIP pts with IgG4 seropositivity and jaundice are at a higher risk of relapse and they could, therefore, be

						showed good response. Spontaneous remission was noted in 65.0% (13/20) pts who neither received CST nor were treated by surgical resection (the median follow-up was 52.8 mos). IgG4 seropositivity is a significant independent factor predictive of spontaneous remission (OR 0.032; 95% CI 0.002–0.426; p = 0.01).	pts who received CST, relapse was noted in 45.2% (19/42). The relapse rate in AIP pts who received CST for 6–12 mos was 80% (12/15), and that in pts who received CST for more than 12 mos was 25.9% (7/27); (p < 0.01). IgG4 seropositivity (OR 10.5, p = 0.04) and the presence of jaundice (OR 6.9, p = 0.02) are significant independent factors predictive of relapse in AIP patients.	MST: continued for more than 6 mos.	candidates for over 3 years of MST. AIP pts with IgG4 seronegativity have a high likelihood of spontaneous remission. IgG4 seropositivity is a significant independent factor predictive of spontaneous remission. IgG4 seropositivity and the presence of jaundice are significant independent factors predictive of relapse in AIP patients.
**Matsubayashi (59) 2011, Japan**	IgG4, IgG, ANA and RAPA, AMA and SMA, SS-B, anti-DNA antibody, SS-A and anti-RNP antibody.	AIP (n=27: high IgG4 (n=20); normal IgG4 (n=7)	JPS 2006 Asian	Serum IgG ≥ 1800 mg/L, IgG4 ≥ 135 mg/dL, IgG1 ≥ 748 mg/dL, ANA and RAPA: ≥80-fold, AMA and SMA: ≥ 40-fold, SSB: >10 U/mL, anti-DNA antibody: >6 IU/ml, SS-A and	Normal IgG4 vs. high IgG4 AIP: Mean IgG4: 48.0±37.1 vs. 516.5±302.4 (p=0.0001); Mean IgG: 1318.9±405.2 vs. 1883.9±431.9 (p=0.006); Mean IgG1: 679.9±170.0 951.5±243.5 (p=0.01).	Response to steroids (n=23) was recognized in all cases, regardless of serum IgG4 level.	Relapse ratio was higher in high-IgG4 AIP (15.8%) than in normal-IgG4 AIP (0%), but was NS. MST was required more frequently amongst patients with elevated compared to normal IgG4 (85.7% vs. 33.3%, respectively; p = 0.04).	Initial: 30–40 mg/d of PSL.<br><br>Tapering at the recommended rate.<br><br>MST: was defined as >1 year of continuing steroid administration.	Compared to patients with normal serum IgG4 levels, patients with elevated IgG4 had a higher incidence of jaundice at onset, more frequent diffuse pancreatic enlargement at imaging, and more frequent EPL. Response to steroids was recognized regardless of serum IgG4 level; however, MST was required more frequently amongst patients

				anti-RNP antibody: any positive					with elevated compared to normal IgG4.
**Takuma (91) 2011, Japan**	IgG4	AIP (n = 50: initially treated with surgical procedures (n = 10); treated with CST (n = 29); conservatively followed up (n = 11)).	Asian	IgG4 > 135 mg/dL	The median serum IgG4: 562.0 mg/dL (range: 11–2490 mg/dL).<br><br>Pt with relapse vs. pts without relapse:<br>Median IgG4 before therapy:<br>450.0 (405.5–1825.0) vs. 289.0 (124.5–568.2);<br>p = 0.02.<br>Median IgG4 after therapy:<br>155.0 (69.5–422.5) vs. 86.0 (20.5–154.0);<br>p = 0.12.	All patients initially treated with steroids responded. The elevated serum IgG4 levels of 25 pts before CST decreased in all pts, but normalized (≤ 135 mg/dL) 2 mos after in only 11 pts (44%).	AIP relapsed in five pts during MST (n = 4) or after the cessation of steroids (n = 1) between 4 and 24 mos after the start of CST. All pts showed re-elevation of serum IgG4 levels at relapse. All relapsed pts responded to treatment with dose-up steroids (n = 4) or retreatment with PSL (30mg/d) (n = 1). None of the pts relapsed after retreatment. Univariate analysis indicated a significant association of relapse with the presence of hilar bile duct stenosis and elevated serum IgG4 levels before steroid therapy (p < 0.05). However, multivariate analysis revealed NS parameters.	Initial: oral 30- 40 mg/d of PSL for 2-3 wks;<br>Tapering: by 5 mg every 1-3 wks until reaching 5mg/d;<br>MST: 1.25–20 mg/d for a median of 38 mos.	Most AIP pts treated with CST have good short-term and long-term outcomes clinically, morphologically, and functionally. Hilar bile duct stenosis and elevated serum IgG4 levels were predictors of relapse of AIP after steroid therapy at univariate analysis. As some pts developed pancreatic stone formation or a malignancy during or after CST, AIP should be followed up strictly.
**Sugimoto (85) 2015, Japan**	IgG, IgG4	AIP type 1 (n = 24)	ICDC	IgG4 ≥ 135 mg/dL	There were NS differences between the oral and pulse	There were no significant differences	The 5-year cumulative relapse-free survival rate did	(1) Oral: Initial: oral 30 mg/d of PSL;<br>Tapering: by 5 mg	Serum IgG4 was reduced significantly after

					groups for serum IgG4 before treatment (367.5±287.2 vs. 476.0±409.3 mg/dL, p = 0.27) and PSL dosage until serum IgG4 reached a minimum level (3744.5 ±2690.9 vs. 4156.9±3344.5 mg, p = 0.33). The serum IgG4 level was significantly reduced from 367.5±287.5 mg/dL before treatment to 124.3±129.3mg/dL after treatment in the oral group (p = 0.012), and from 476.0±409.3 mg/dL before treatment to 110.3±147.6mg/dL after treatment in the pulse group (p < 0.001).	between the oral and pulse groups for serum IgG4 before treatment (367.5±287.2 vs. 476.0±409.3 mg/dL, P = 0.27) and PSL dosage until serum IgG4 reached a minimum level (3744.5 ±2690.9 vs. 4156.9 ±3344.5 mg, p = 0.33). The serum IgG4 level was significantly reduced from 367.5± 287.5 mg/dL before treatment to 124.3±129.3mg/dL after treatment in the oral group (p = 0.012), and from 476.0±409.3 mg/dL before treatment to 110.3±147.6mg/dL after treatment in the pulse group (p < 0.001).	not differ significantly between the oral and pulse groups (46.9% vs. 77.8%, p = 0.098). However, in a subset of cases with diffuse pancreatic swelling, this rate was significantly lower in the oral group (33.3% vs. 100.0%, p = 0.046).	every 4 wks down to 10 mg, then further reduced in 2.5-mg increments. (2) Pulse: Initial: intravenous 250 mg/d or 125 mg/d methylprednisolone, followed by 20 mg oral PSL; Tapering: as in (1).	therapy in both groups and the 5-year cumulative relapse-free survival rates in the two groups was NS. The differences were NS in the effect on disease activity between steroid pulse therapy and oral CST. However, steroid pulse therapy was more effective than oral CST in pts with diffuse pancreatic swelling, and these pts may be suitable candidates for steroid pulse therapy.
**Hirano (36) 2016, Japan**	IgG4, IgG	Type 1 AIP (n = 21)	Asian	IgG > 1600 mg/dL for defining serological relapse	Mean IgG values during aggravation and remission were 1719±444 and 1310±198 mg/ dL (p = 0.0197), respectively, whereas the corresponding IgG4 values were 493±406 and 224±143 mg/dL	N/A	During the 43-mo (range, 19 to 48 mo) follow-up period, clinical relapse occurred in 10 pts. Serological relapse was observed in 12 pts. Although clinical and serological relapse occurred	Serologically stable pts receiving CST therapy for at least 3 years were observed during the follow-up. Tapering: by 1mg every 8 to 10 wks until complete cessation.	Cessation of CST may lead to a high rate of clinical relapse, even in pts with long-term MST (> 3 y). Therefore, it is desirable to continue MST for a period > 3 years to prevent clinical

					(p = 0.0724), respectively. The IgG cutoff value of 1400 mg/dL had Sn, Sp, and accuracy of 90% (9/10), 73% (8/11), and 81% (17/21), respectively. The Sn and Sp of IgG values > 1600 mg/dL were 77% and 94%, respectively, whereas the corresponding IgG4 values > 244 mg/dL were 81% and 67%, respectively. On the basis of this, serological relapse was defined as IgG > 1600 mg/dL.		concomitantly in 3 pts, serological relapse preceded clinical relapse in 4 pts. Five pts experienced serological relapse alone, and no clinical or serological relapse occurred in 6 pts. IgG and IgG4 levels when starting to taper CST, as well as the IgG and IgG4 at the initial diagnosis of AIP, were NS predictors of relapse. The only significant factor for predicting relapse was duration of CST.	Thereafter, pts were reviewed every 3 mos. Upon resuming CST, the initial PSL dose administered was 10 mg/d for 8 to 12 wks; this was gradually tapered to 5 mg/d.	relapse. Tracking serological markers and adjusting CST accordingly may contribute to prevention in some pts, but this method is often ineffective.
**Shimizu (82) 2016, Japan**	IgG4	AIP type 1 (n = 47)	JPS ICDC	IgG4 g/dL before CST begins, Δ: measured within 4 mos after CST begins, Δ′: measured within 2 mos after CST begins, Cut-off for Δ′ value: 4.3, 10.7, and 13.2.	Relapse vs. non-relapse:<br>Median IgG4 before CST:<br>382 mg/dL vs. 566 mg/dL (p = 0.2).<br>Median first IgG4 levels measured within 4 mos after the start of CST:<br>162 mg/dL vs. 251 mg/dL (p = 0.3)<br>Δ value: 3.8 vs. 18.3 (p = 0.01).<br><br>AIP patient subgroup (n = 34).<br>Median IgG4 before CST:<br>433 mg/dL vs. 748	All pts responded to CST: relapse group (n = 17); non-relapse group (n = 30).	The AIP relapse group consisted of 17 pts. AIP pts with a rapid drop in IgG4 level in response to initial CST were less likely to experience a relapse. There was an association between the rate of decrease in the serum IgG4 level of AIP patients in response to initial steroid therapy and their relapse rate.	Initial: oral PSL 0.6 mg/kg/d for 2 wks;<br>Tapering: by 5 mg every 2 to 4 wks individually tailored until reaching 2.5 to 5 mg/d.<br>MST: dose of 2.5 to 5 mg/d, with a discontinuation within 3 years of the therapy start in those with no relapse.	Low rate of decrease in serum IgG4 levels before the start of CST compared to the first serum IgG4 measurement made within 2 mos after the start of steroid therapy was associated with a relapse of AIP, suggesting that the rate of decrease in serum IgG4 level may serve as a predictor of relapse of AIP.

					mg/dL (p = 0.1).<br>Median first IgG4 levels measured within 2 mos after the start of CST:<br>162 mg/dL vs. 261 mg/dL (p = 0.2)<br>Δ′ value:<br>3.8 vs. 16.4 (p = 0.01).<br><br>The AUC of the curve for discriminating between the 2 groups was 0.781, and at the 3 candidate Δ′ cutoff values of 4.3, 10.7, and 13.2, the Sn of the Δ′ values for discriminating between the relapse group and non-relapse group were 0.69, 0.85, and 0.92, respectively, and Sp values were 0.79, 0.63, and 0.58, respectively.				
**Lee (56) 2018, South Korea**	IgG, IgG4	AIP type 1 (n = 138)	ICDC	IgG > 1800 mg/dL<br>IgG4 > 135 mg/dL<br>IgG4 > 270 mg/dL	Initial serum IgG levels were elevated in 63 (45.7%) pts, IgG4 levels were elevated in 74 (53.6%) pts, and IgG4 levels were over twice the cut-off value in 47 (34.1%) pts. Serum IgG4 level was higher in relapsed pts than in those with no relapse (306.7 vs. 209.9 mg/dL, mean, p = 0.024).	All 138 pts achieved clinical remission with initial CST, and 66 (47.8%) experienced relapse during a median 60-month (range 24–197) follow-up. Among the relapsed pts, about 74% (49/66) relapsed within 3 years.<br>In 74 pts with	66 pts (47.8%) experienced relapse during a median 60-month (range 24–197) follow-up.<br>Among the relapsed pts, about 74% (49/66) relapsed within 3 years.<br>The relapse rate was significantly lower in pts who had normalized IgG4 levels at the end of tapering after CST	Initial: 30–40 mg/d of oral PSL for 1–2 mos;<br>Tapering: by 5–10 mg per mo;<br>MST: 5 mg/d continued for an average of 6 mos and then completely stopped.	Type 1 AIP has common relapses, and pts with OOI, especially OOI of the proximal bile duct, appear to be at increased risk for relapse.<br>The relapse rate was significantly lower in pts who had normalized IgG4 levels at the end of tapering after CST than in those who

						elevated IgG4 prior to CST, the serum IgG4 level decreased in 67 (90.5%) pts and returned to the normal range in 43 (56.6%) pts at the end of tapering after CST.	than in those who did not (41.9 vs. 74.2%, p = 0.006). Obstructive jaundice was not a significant predictor of relapse, although total bilirubin level was higher in the relapse group than in those with no relapse (4.5 vs. 2.3 mg/ dL, mean, p = 0.021). The involvement of the proximal bile duct at the first diagnosis was a significant independent predictor of relapse (HR 2.65; 95% CI 1.44–4.89; p = 0.002).		did not (41.9 vs. 74.2%, p = 0.006).
**Suzuki (86) 2018, Japan**	IgG4	AIP (n = 73)	ICDC	IgG4: Normal (< 135 mg/dL); Level 1 (≥ 270 mg/dL); Level 2 (135–269 mg/dL).	Median IgG4 levels before treatment (total 383 (237–520): Level 1: 616 (549–1010); Level 2: 220 (184–233); Normal: 72.3 (22.7–99.3).<br><br>Median IgG4 level after CST (total 165 (106–223): Level 1: 616 (549–1010); Level 2: 220 (184–233); Normal: 72.3 (22.7–99.3).	The median serum IgG4 levels decreased after CST in all groups. There were 39 (95%) cases in the level 1 group, 2 (12%) cases in the level 2 group, and 0 (0%) cases in the normal group that had IgG4 > 135 mg/dL after CST. The relative IgG4 decrease in the level 1 and level 2 groups was significantly greater than that	Relapse was detected in 24 (33%) cases. There were NS differences in relapse rates between the 3 groups. The average time from starting CST to relapse was 1084 days. There was no association between relapse and serum IgG4 levels before and after CST between the relapse and no-relapse cases. Relative rise in IgG4 levels over the period from 2 to 4 mos after starting	Initial: oral PSL 0.6 mg/kg/d for 2-4 wks; Tapering: by 5 mg every 1- 2 wks until an MST dose of 5 mg/d was reached.	Elevated serum IgG4 levels are closely associated with the existence of multiple IgG4-related EPL, but not with an increased risk of relapse. However, post-treatment serum IgG4 rises of more than 1.25 times during the follow-up period might be predictive of relapse. In cases where such rises are detected, the tapering or withdrawal of CST

					Relative rise in serum IgG4 levels in the relapse and non-relapse cases: At a 0.23674 cut-off value, there was 100% Sn and 53% Sp for relapse and non-relapse cases.	of the normal group.	CST and the time of relapse were significantly higher in the relapse cases than those in the non-relapse cases in all 3 groups.		should be carefully considered.
**Miki (61) 2019, Japan**	IgG4	AIP type 1 (n = 115)	ICDC	/	NS difference was found between median IgG4 in pts who relapsed and those who did not relapse (1957 (1030-4225) mg/dL vs. 1803.5 (966-2361) mg/dL). The IgG4 level was higher in pts with DS ($p = 0.013$), and the IgG level was higher in pts with SC and RF than in the groups without ($p = 0.009$ and $p = 0.005$, respectively).	Remission was achieved in all patients, except one.	No relationship was found between relapse and the number of EPL. No correlation was observed between serum IgG4 level and relapse.	Initial: oral PSL 0.6 mg/kg/d; Tapering: by 5-10 mg every 1 to 2 wks; MST: 2.5-12.5 mg/d was continued for over 6 mos.	The number of EPL and IgG4 levels at onset was positively correlated. However, no relationship was found between relapse and the number of EPL. Furthermore, NS difference was found between IgG4 in pts who relapsed and those who did not. The interval between symptom onset and steroid initiation, and the presence of retroperitoneal fibrosis at onset were significant risk factors for relapse with IgG4-related sclerosis cholangitis and IgG4-related retroperitoneal fibrosis, respectively.

**Fukiage (28) 2020, Japan**	Autotaxin (ATX) IgG4	AIP type 1 male pts (n = 24)	ICDC	Based on ROC curve: ATX 0.909 mg/L	Serum ATX was higher in females than in males (p = 0.002). The median value of serum ATX at diagnosis was 1.003 (IQR 0.807–1.109 mg/L in male pts (n = 24), whereas median IgG4 level was 351 (IQR 285–674) mg/dL. The ability of maintenance ATX (with a cut-off of 0.909 mg/L) in combination with elastase-1 at diagnosis (cut-off of 236.5 ng/dL) was evaluated by ROC curve analysis for predicting relapse. Sn, Sp, accuracy, PPV and NPV of this model were 100%, 91%, 95%, 91% and 100%, respectively. AUC was 0.98 (95% CI 0.92–1.00).<br><br>Of the 56 pts, 21 pts (37.5%) had EPL. However, none of EPL showed a significant association with AIP relapse or serum ATX level.	Induction ATX and maintenance ATX were decreased in comparison with baseline ATX. The maintenance ATX was slightly but significantly higher than induction ATX. Serum IgG4 showed similar trends to those seen in ATX, but the apparent increase from induction IgG4 to maintenance IgG4 was NS.	In the 12 pts who relapsed, relapse ATX was elevated compared with that of induction therapy (induction ATX in relapsed pts). Maintenance ATX was also higher than induction ATX in relapsed pts. On the other hand, IgG4 gradually increased between induction and relapse, but the change was NS.<br><br>Comparison of ATX and IgG4 between pts with and without relapse: A significant difference between the two groups was only observed in maintenance ATX .<br><br>No EPL showed a significant association with AIP relapse or serum ATX level.	Initial: oral PSL 0.6 mg/kg/d for 2-4 wks.<br><br>Tapering: by 5 mg gradually.<br><br>MST: 2.5–5 mg/d at the discretion of the attending physician.	In this study of Japanese type 1 AIP pts, serum ATX significantly decreased after induction therapy, whereas it increased at the time of relapse. The increase in ATX from induction to maintenance therapy was associated with the relapse rate. Serum ATX is a novel biomarker which might be useful to predict the relapse of AIP as well as to monitor the CST effect.
**Ishikawa (41) 2020, Japan**	IgG4 CA19-9	AIP (n = 123): definitive AIP type 1 (n = 102),	ICDC	IgG4 > 140 mg/dL; IgG4 > 280	During the median follow-up period of 55 mos (IQR, 27–98),	N/A	Levels of IgG4 (> 280 mg/dL) and CA 19-9 (> 40 mg/dL) at the	Initial: oral PSL 0.6 mg/kg/d for 2-4 wks.	Pts with AIP and focal pancreatic enlargement and/or

	probable AIP type 1 (n = 7), and NOS-AIP (n = 14).		mg/dL; CA 19-9 > 40 U/mL.	two pts (1.6%) were diagnosed with pancreatic cancer. The median IgG4 levels at appearance of pancreatic relapse vs. pancreatic cancer was 333 vs. 316 mg/dL (p = 0.9). The median CA19-9 at the initial dg of AIP was significantly higher in pancreatic cancer pts (117 and 324 U/mL) than in pancreatic relapse pts (21 U/mL).		onset of AIP did not predict relapse. There were NS differences in serology levels, including IgG4 and CA19-9 between the pts with pancreatic and extra pancreatic relapses. There were NS difference between AIP patients with relapse (n = 22) and AIP with pancreatic cancer (n = 2) in median IgG4 levels. However, median CA19-9 at initial AIP diagnosis was higher in AIP with pancreatic cancer (220.5 U/mL) than in AIP with relapse (21 U/mL) (p = 0.01). Lack of maintenance therapy (p = 0.02, HR 2.1), IgG4–sclerosing cholangitis (p = 0.04, HR 2.7), and IgG4–kidney disease (p = 0.02, HR 2.95) were identified as independent predictors of relapse.	Tapering: by 5-10 mg every 1 -2 wks until reaching the maintenance dosage of 2.5-10 mg/d.<br><br>MST: more than 3 years or until relapse.<br><br>* pts with focal tumorous lesions underwent a trial with CST for 2 wks and then received MST.	MPD dilation who exhibited high serum CA 19-9 levels at diagnosis should be closely monitored for the development of pancreatic cancer. Lack of maintenance therapy, IgG4–sclerosing cholangitis, and IgG4–kidney disease were identified as independent predictors of relapse.

CST: corticosteroid treatment; SD: standard deviation; Sp: specificity; Sn: sensitivity; PPV: positive predictive value; NPV: negative predictive value; IgG4: immunoglobulin G4; IgG: immunoglobulin G; IgE: immunoglobulin E; CIC: circulating immune complexes; HC: healthy controls; CP: chronic pancreatitis; PBC: primary biliary cirrhosis; PSC: primary sclerosing cholangitis; SjS: Sjogren's

syndrome; PSL: prednisolone; AIP: autoimmune pancreatitis; pts: patients; wk: weeks; mo: months; JPS: Japan Pancreas Society; MST: maintenance steroid therapy; AIC: autoimmune cholangitis; EPL: extrapancreatic lesions; OR: odds ratio; AUC: area under the curve; HR: hazard ratio; CI: confidence interval; DS: dacryosialadenitis; SC: sclerosing cholangitis; RF: retroperitoneal fibrosis; Δ: the rapidity of the decrease in serum IgG4 level in response to initial steroid therapy; CA19-9: carbohydrate antigen 19-9; NOS-AIP: not-otherwise-specified AIP by ICDC; IQR: interquartile range; ROC: receiver operating characteristics; OOI: other organ involvement; ANA: anti-nuclear antibodies; AMA: anti-mitochondrial antibodies; ASMAs: anti-smooth muscle antibodies; anti-RNP: anti-nuclear ribonucleoprotein antibodies; anti-SSA: anti-Sjögren's-syndrome-related antigen A; anti-SSB: anti-Sjögren's-syndrome-related antigen B; NS: not significant.

Supplemental Table S8. Role of autoantibodies in AIP.

Author, Year, Country	Candidate biomarker	Detection method	Cohort (n)	Criteria	Frequency of marker elevation in AIP (%) vs. controls (%)	Mean/median Sn, Sp, PPV, NPV	Conclusion
**Okazaki (71) 2000, Japan**	Anti-LF Anti-CA–II	ELISA and IIF	AIP (n = 17: primary AIP (n = 11), secondary AIP (n = 6), alcoholic CP (n = 17, gallstone–related CP (n = 17), HC (n = 17))	Histology, cytology, imaging, and response to steroids	In the AIP cohort, ANA was detected in 13 (76%) pts, anti-LF in 13 (76%), anti-CA–II in 10 (59%), RF in 5 (29%), ASMA in 3 (18%), but AMA in none (0%). The serum levels of ACA-II and LF Ab were not correlated. Anti-LF, anti-CA–II, ASMA, and AMA were not present in the non-AIP and control groups. ANA was positive in one pt with alcoholic CP.	Mean serum anti-LF in pts with AIP (1.276± 0.276) was significantly higher than in gallstone-related pancreatitis (0.513±0.165), alcoholic CP (0.628±0.113), and HC (0.369±0.122).<br><br>Mean serum levels of anti-CA–II in pts with AIP (1.032±0.445) were significantly higher than in gallstone-related pancreatitis (0.513 ±0.127), alcoholic CP (0.613 ± 0.268), and HC (0.541 ± 0.122).<br>* values expressed as OD 490 nm.	Study showed a high prevalence and serum levels of anti-CA–II and anti-LF in AIP in comparison with those in gallstone-related and alcoholic CP and in HC. This suggests that CA–II and LF may be candidates for the target antigens. However, the serum levels of anti-LF and anti-CA–II were not correlated with heterogeneous prevalence in different cohorts. Anti-CA–II and anti-LF are not necessarily specific for AIP.
**Nishimori (69) 2005, Japan**	Anti-CA–IV	ELISA	AIP definite (n=15), AIP probable (n=14), alcoholic CP (n=15), pancreatic cancer (n=14), SjS (n=20), HC (n=26).	JPS	Truncated CA IV-positive sera: definite AIP 26.7% (4/15), probable AIP 42.9% (6/14), alcoholic CP 13.3% (2/15), pancreatic cancer 14.3% (2/14), SjS 45% (9/20), HC 0% (0/26).<br>CA IV peptide 4 positive: definite AIP 40% (6/15), probable AIP 21.4% (3/14), alcoholic CP 13.3%	The most notable difference between the Ab-positive and -negative pts was serum levels of gamma-globulin (p<0.001). Moreover, there were significant differences in levels of IgG (p<0.001) and IgA (p<0.05). Furthermore, serum IgG levels showed correlations with Ab titers to	AIP pts with positive serum Ab to CA IV were shown to have increased serum levels of gamma-globulin, especially IgG and IgA, indicating that they were in a hyperimmune state. However, morphological changes in the pancreas, proposed as a clinical characteristic of AIP, were not

					(2/15), pancreatic cancer 7.1% (1/14), SjS 40.0% (8/20), HC 0% (0/26).	CA IV peptide 4 (r=0.80) and to N2/C2–CA IV protein (r=0.72).	associated with the presence of serum Ab to CA IV.
**Asada (14) 2006, Japan**	Anti-PSTI<br>Anti-LF<br>IgG4	ELISA, Western blotting. nephelometry.	AIP (n = 26), alcoholic CP (n = 10), idiopathic CP (n = 10), acute pancreatitis (n = 17), pancreatic ductal cell carcinoma (n = 16), and HC (n = 12).	JPS	Auto-Ab against PSTI were detected in 30.8% of the sera from patients with AIP by ELISA and 42.3% by Western blotting. Among pts with AIP, auto-Ab against PSTI were significantly positive in 42.3% (11/26) using Western blotting and in 30.8% (8/26) using ELISA, respectively. However, none of the control subjects were positive for anti-PSTI Ab.	The OD values of IgG against PSTI in pts with AIP (0.268 ± 0.190) were greater ($p < 0.05$) than those of pts with alcoholic CP (0.135± 0.029), idiopathic CP (0.118 ± 0.054), acute pancreatitis (0.138 ± 0.062), or pancreatic ductal cell carcinoma (0.122 ± 0.063) and those of HC subjects (0.125 ± 0.064).<br>Anti-LF seemed to be the most useful Ab for diagnosis. The diagnostic Sn was slightly increased from 73.1% to 76.9% with the combination of anti-LF and anti-PSTI Ab compared with anti-LF alone.	The present study showed the elevation of anti-PSTI Ab in 30% to 40% of pts with AIP, and 2 of 3 pts that were negative for both anti-LF and ant-CA-II were positive for anti-PSTI Ab. These findings suggest that some pts with AIP can be screened or diagnosed by only anti-PSTI Ab, although a combination of anti-LF and anti-PSTI Ab did not remarkably increase Sn.
**Hosoda (38) 2008, Japan**	Anti-CA–II<br>IgG4	ELISA, nephelometry	AIP (n = 9), SjS (n = 51), alcoholic CP (n = 24), IPMN (n = 24), pancreatic cancer (n = 32), HC (n = 40) .	JPS	Elevated anti-CA–II levels were shown in 89% of AIP pts, 74% of primary SjS pts, 53% of secondary SjS pts, 46% of alcoholic CP pts, and 16.7% of pts with IPMN, but not in (0%) pancreatic cancer pts. Compared with the results in HC, the Ab-positive prevalence rate was higher in pts with AIP ($p < 0.02$), primary SjS ($P < 0.01$), and alcoholic CP ($p < 0.01$).<br>Serum IgG4 was detected in 89% of AIP pts. Both serum IgG4 and anti-CA-II antibodies were detected in 78% of these pts with AIP. NS correlation was observed between the serum IgG4 levels and the anti-CA-II Ab levels in sera from pts with AIP ($r = 0.198$).	Mean values of anti-CA II in patents with AIP (0.153 ± 0.040), SjS (0.169 ± 0.072): primary SjS (0.180 ± 0.073), secondary SjS (0.148 ± 0.069), alcoholic CP (0.141 ± 0.062), IPMN (0.075 ± 0.045): main duct type (0.085 ±0.048), branch duct type (0.071 ± 0.045), pancreatic cancer (0.053 ± 0.030), and HC (0.081 ± 0.025).<br>*Results are expressed as OD 490 nm.<br><br>Sn and Sp of the anti-CA-II test for the differential diagnosis of AIP versus non-AIP were 89% and 63%, respectively, whereas those for inflammatory versus neoplastic disease of the pancreas were 67% and 95%, respectively.	Anti-CA-II Ab is probably not a specific marker of AIP because it was present at a higher frequency in the sera of pts with other diseases. Nevertheless, anti-CA-II Ab may be a useful tool for the differential diagnosis of AIP and pancreatic cancer. These results suggest that elevated serum IgG4 and anti-CA-II antibody levels may be manifestations compatible with AIP. However, no significant correlation was seen between the serum IgG4 levels and the anti-CA-II Ab levels ($r = 0.198$).

**Endo (23) 2009, Japan**	Anti-amylase α-2A, anti-LF, anti-CA–II	ELISA	AIP (n = 15), alcoholic CP (n = 25), pancreatic cancer (n = 25), fulminant T1bDM (n = 17), acute onset T1DM (n = 42), T2DM (n = 67), 100 HC (n = 100).	JPS	Positive IgG Ab against amylase α-2A were found all AIP pts, but in none of the CP and pancreatic tumor pts. HC were all negative except for one subject (1%) with positive anti-amylase α-2A IgG ($p<0.001$). Interestingly, 88% of pts with fulminant T1bDM, 21% of acute-onset T1DM, and 6% of T2DM were positive for anti-amylase α-2A.<br><br>Thirteen AIP samples were tested for anti-CA II and anti-LF: 66% (10/15) were positive for CA–II and 53% (8/15) were positive for LF.<br><br>* In two AIP pts, levels of anti-amylase α-2A decreased rapidly after CST parallel to IgG4. The Ab level did not increase even at the drug maintenance dose in both cases.	To obtain the cut-off value for anti-amylase α-2A positivity, ROC analysis of the HC (n=100) and fulminant T1bDM (n=17) was performed; when the value was set at 34 (area under the ROC 0.92; p=0.0001) Sn, Sp, and PPV were 88%, 99% and 94%, respectively.	Anti-amylase α-2A is a novel diagnostic marker for both AIP and fulminant T1bDM, which are clinically and immunologically closely related. The Ab against amylase α-2A might be a more sensitive marker for AIP than that of CA–II, LF, or PSTI.
**Frulloni (26) 2009, Italy**	Anti-PBP IgG4	DELFIA, ELISA, Western blotting	Training group: AIP (n = 20), pancreatic, cancer (n = 40), alcohol-induced CP (n = 21), IPMN (n = 18), systemic sclerosis (n = 17), rheumatoid arthritis (n =20), HC (n =40).<br><br>Validation group: AIP (n =15) and	Histology, cytology, imaging, response to steroids.	When the training and validation groups were combined, 94% pts with AIP (n = 35) and 5% with pancreatic cancer (n =110) had a positive test for Ab, with a Sn of 94% and a Sp of 95%.<br>IgG4 Ab was positive in 54% of AIP pts (n= 35), whereas 10% (n =110) of pancreatic cancer pts were IgG4-positive.	The Sn and Sp of the quantitative analysis of the assay, with a cut-off value of 32,000 IU, were 95% and 97%, respectively. The AUC in the ROC analysis was 0.99 (95% CI, 0.97 to 1.00; $p<0.001$).<br>When comparing AIP with only pts with pancreatic cancer, the Sn and Sp were 95% and 90%, respectively, with an AUC of 0.97 (95% CI, 0.94 to 1.00; $p<0.001$).<br>Employing the validation group, an AUC of 0.99 (95% CI, 0.96 to 1.00; $p<0.001$) was reported. Overall, the test had a Sn of 94% and a Sp of 95%.	After merging the training and validation groups, the assay exhibited an outstanding diagnostic performance (Sn 94%, Sp 95%) in discriminating between AIP and pancreatic cancer. Because 5–10% pts with pancreatic cancer were reactive, the test was not perfect for AIP diagnosis. Interestingly, there were > 80% pts with H. pylori-positive serology status in both the training and validation AIP groups.

			pancreatic cancer (n = 70).				
**Löhr (58) 2010, Germany and Italy**	Ab against Trypsinogens (PRSS1, PRSS2) and anti-PSTI/SPINK1	Genomics and proteomics, immunohistochemistry, Western blotting, and ELISA.	AIP (n = 19), non-AIP CP pts (n = 23), HC (n = 121)	HISORt, histopathological criteria	N/A	Anti-PRSS1 (ng/mg): 68.9±39.6 (AIP), 37.9±11.9 (alcoholic CP), 30.4±12.9 (HC);<br>Anti-PRSS2 (ng/mg): 25.6±10.1 (AIP), 19.4±7.5 (ACP), 9.2±5.6 (HC);<br>Anti-SPINK1 (pg/mg): 7.1±2.8 (AIP), 5.6±4.5 (ACP), 3.3±1.9 (HC);<br>Ab serum data had a predictive accuracy of 80% for distinguishing pts with AIP from those with non-AIP CP, and an accuracy of 86% for AIP pts vs. HC. The Sn of the Ab test was 68% for AIP vs. non-AIP CP and 79% for AIP vs. controls. The Sp was 90% for AIP vs. non-AIP CP and 95% for AIP vs. controls.	Acinar cells, in addition to ductal cells, are a target of the immune-related inflammatory process characterizing AIP. Clinically, the loss of the acinar cells is associated with elevated Ab titers for PRSS1, PRSS2 and PSTI. The detection of these Ab by ELISA may help to distinguish AIP from other types of pancreatitis such as alcoholic pancreatitis. It seems that the loss of acinar cells and the occurrence of auto-Ab to trypsinogens are findings typical for both subtypes of AIP.
**Sanchez–Castanon (80) 2012, Spain**	Anti-CA–II,<br>anti-LF<br>anti-amylase α-2A<br>IgG, IgG4	ELISA, nephelometry	AIP (n = 12), CP (n = 23), idiopathic CP (n = 26), acute pancreatitis (n = 11), pancreatic cancer (n = 21), SjS (n = 9) and T1DM (n = 40), HC (n = 45).	HISORt	Serum levels of both total IgG and IgG4 were elevated in 50% (6/12) AIP pts. There was one patient with only total IgG increased and another one with only IgG4 increased.<br>Both anti-CA–II and anti-α-amylase α-2A were positive in 8 of 10 pts with AIP. There was significant difference in prevalence of anti-CA–II in AIP vs. different groups: 9% in CP, in 50% idiopathic CP, 54% acute pancreatitis, 29% in pancreatic cancer, 67% in SjS, 32% in T1DM, and 8% in HC. A significant difference in anti-α-amylase α-2A levels was also shown comparing AIP pts with other groups: 13% in CP, in 31% idiopathic CP, 18% acute pancreatitis, 0% in pancreatic	In AIP pts, the median IgG and IgG4 level were 1550 mg/dL (range 785–3110) and 136 mg/dL (range 26–4990), respectively.<br>When diagnosing AIP, a combination of anti-CA–II and anti-amylase α-2A had the highest Sn (83%) although anti-amylase α-2A (89%) was more specific than anti-CA–II (75%). The presence of increased IgG4 levels was the most specific serological marker (94%), but it had the lowest Sn (58%). The combination of the three markers altogether had the highest Sp (99%) and PPV (86%), but they had a rather low Sn (50%). The combination of anti-CA–II and anti-amylase α-2A without IgG4 levels yielded the highest Sn (75%) and NPV (98%) but the Sp and the PPV decreased to 93% and 50%, respectively. Importantly,	The presence of serum anti-CA–II and anti-amylase α-2A with increased IgG4 is useful in the differential diagnosis of AIP from pancreatic cancer.

					cancer, 23% in SjS, 22% in T1DM, and 2% in HC. Interestingly, none of the pts with AIP were positive for anti-LF.	anti-amylase α-2A was not detected in pancreatic cancer.	
**Felix (24) 2013, Germany**	Ab to 15 different *H. pylori* proteins IgG4 and IgG	ELISA, nephelometr y	PDAC (n = 270), alcoholic CP (n = 290), AIP (n = 32), HC (n =127), other GI cancers (n =165).	HISORt	H. pylori seropositivity was defined as seropositivity to >3 H. pylori proteins. H.pylori seroprevalence was 43.8% in AIP, 56.9% in CP, 64.7% in PDAC, 69.7% in different GI cancers, and 50.4% in controls.<br>From the analyzed AIP samples (n= 32), three pts were characterized with abnormal total IgG (>16 g/l), and 18 samples (59%) showed elevated IgG4 levels (> 1.4 g/L, range 1.8–16.6 g/L).	N/A	The present results may form the basis of a combined blood test to discriminate/differentiate AIP from pancreatic cancer in the future. Such a test would be of great value particularly for patients where the CA 19–9 marker is in normal range (< 37 U/ml). The evaluation of present biomarkers candidates in the sera of patients with normal CA 19–9 values revealed significant differences between AIP and PDAC for apolipoprotein A–I, tetranectin, and transthyretin.<br>Among pts with pancreatic disease, there was no significant association of H.pylori infection with AIP.
**Talar–Wojnarowska (92) 2014, Poland**	Anti-CA–II IgG, IgG4	ELISA	PDAC (n = 45), type 1 AIP (n = 24) and CP (n = 55).	ICDC	However, 16 (35.5%) pts with PDAC and 14 (25.4%) pts with CP had IgG4 levels greater than 140 mg/dL. Moreover, in 3 (6.67%) pts with PDAC, those values were greater than 280 mg/dL. No pts with CP had IgG4 more than 280 mg/dL.	In AIP pts, the median IgG levels were 19.7 g/L, IgG4 levels were 301.9 mg/dL, and anti-CA-II levels were 81.82 ng/mL, compared to 10.61 g/L, 123.2 mg/dL and 28.6 ng/mL, respectively, in PDAC pts. Serum levels of IgG, IgG4 and anti-CA-II were significantly higher in pts with AIP compared to PDAC and CP pts.<br><br>IgG4 with the cut-off of 210 mg/dL showed the best Sn and Sp (83.8% and 89.5%) in AIP diagnosis compared to IgG (69.3% and 87.3%, respectively; cutoff 15 g/L) and anti-CA-II (45.3%	IgG4 at the cut-off of 210 mg/dL showed the best Sn and Sp in AIP diagnosis compared to IgG and anti-CA-II-Ab; however, elevations of serum IgG4 may be seen in subjects without AIP, including PDAC.<br>Anti-CA–II had the lowest Sn and Sp compared to other analyzed markers, increasing not only in CP pts but also in pts with PDAC. Because high levels of anti-CA–II are frequently seen in subjects with PDAC, anti-CA II cannot be used to distinguish AIP from PDAC.

						and 74.3%; cut-off 38.4 ng/mL). AUC was 0.940 (95%CI 0.926–0.968) for IgG4, 0.818 (95%CI 0.8–0.897) for IgG and 0.632 (95%CI 0.596–0.719) for anti-CA–II.	
**Yanagisawa (99) 2014, Japan**	Antibody against E. coli antigens anti-CA–II and anti-LF	ELISA, Western blotting	AIP (n = 14), pancreatic diseases other than AIP (n = 16), CP (n = 6), pancreatic cancer (n = 19), and pancreatic disease-free individuals (n = 25).	JPS	Elevations in IgG, IgG4, and IgE were observed in 3 (21%), 13 (93%), and 13 (93%) of the AIP patients, respectively. Sera obtained from AIP pts reacted to FliC from E. coli at a significantly higher level than the levels for CP, pancreatic cancer and pancreatic disease-free controls. Furthermore, the anti-FliC titers obtained in sera from CP and pancreatic cancer were significantly higher than that of pancreatic disease-free controls. The correlation coefficients for the anti-FliC from E. coli titers versus IgG, IgG4, IgE, ANA, and steroid administration in the AIP patient group were NS.	N/A	In conclusion, FliC from E. coli was capable of inducing AIP-like pancreatitis in mice. The titers of a specific Ab for FliC from E. coli were significantly elevated in pts with AIP. Bacterial protein itself might be directly pathogenic or might act as a molecular mimic of an unknown autoantigen in the generation of AIP-like inflammation. A reconsideration of the importance of commensal bacteria as an environmental factor capable of inducing autoimmunity may provide an important insight to overcoming AIP.
**Aparisi (13) 2015, Spain**	Anti-CA–II IgG4, IgG	ELISA, Western blotting, nephelometry	Idiopathic CP (n = 54), alcoholic CP (n = 86), SjS (n = 33), and HC (n = 54).	Score based upon clinical manifestation, imaging, serology, and histology	The percentage of pts with increased serum anti-CA–II was higher in the idiopathic CP group (28%) than in the controls (2%) and in pts with alcoholic CP (11%), but lower than in pts with SjS (64%). The proportion with elevated IgG4 levels was higher in the idiopathic CP group (15%) compared with HC (2%) and SjS (0%) but not significantly different from alcoholic CP (8%). The percentage of cases with elevated levels of IgG was significantly higher in pts with alcoholic CP (36%), idiopathic CP (43%), or SjS (52%) than in HC (20%).	Serum levels of anti-CA–II were significantly higher in pts with alcoholic CP, idiopathic CP, or SjS than in HC. Anti-CA–II levels were also higher in pts with SjS than in those with idiopathic CP.	Results indicate that most cases with increased anti-CA–II exhibited clinical and morphological parameters compatible with AIP. Thus, a significant association was found between increased serum anti-CA–II levels and elevated serum IgG levels or associated autoimmune disorders in the idiopathic CP group. The majority of pts (10/15) with elevated anti-CA–II levels also had increased IgG levels.

					Among the 54 idiopathic CP pts, 13% had a simultaneous increase in anti-CA–II and IgG4 levels while 15% had increased anti-CA–II but normal IgG4 levels. The proportion of cases with elevated IgG4 levels in the idiopathic CP group were significantly higher among pts with increased anti-CA–II than in those with normal values.		
**Sánchez Castañón (79) 2015, Spain and Italy**	Anti-amylase α-2A IgG4	ELISA, nephelometry	AIP (n =25: type 1 AIP (n = 13), type 2 AIP (n = 3) and NOS–AIP (n = 8)), disease control groups (n = 84: CP (n = 31), acute pancreatitis (n = 30), PDAC (n = 23)), and HC (n = 59).	ICDC	Increased IgG4 was detected to be present in 52% of AIP pts, 5% of disease controls, and 0% of HC. IgG4 was elevated in 92% of the 13 pts with type 1 AIP, but in none of the 3 with type 2 or 8 with NOS–AIP.<br>Anti-amylase α-2A was present in 76% of AIP, 36% of disease controls, and 2% of HC. Anti-amylase α-2A was present in 79%, 67%, and 75% of type 1, type 2, and NOS–AIP, respectively.<br><br>Anti-amylase α-2A at the optimal cut-off value was detected in the sera of 29%, 50%, and 26% of pts with CP, AIP, and PDAC, respectively (significant p values). Anti-amylase α-2A was present in 79%, 67%, and 75% of type 1, type 2, and NOS AIP, respectively.	The mean of IgG4 was 323 mg/dL (range, 16–1660 mg/dL). The median of IgG4 was 362 mg/dL (range, 56–1660 mg/dL), 64 mg/dL (range, 41–87 mg/dL), and 52 mg/dL (16–97 mg/dL) in the sera of the type 1, type 2, and NOS AIP pts, respectively.<br>The Sn and Sp for diagnosing AIP of anti-amylase α-2A were 76% and 78%, and for IgG4, 50% and 94%. By combining the 2 serological markers, Sn was 41% and Sp was 99%, with a PPV of 82% and NPV of 92%.	When the combination of both IgG4 and anti-amylase α-2A was employed, Sp increased to 99%, although Sn fell to 41% (PPV and NPV were 82% and 92%, respectively). However, because the main clinical problem is to differentiate AIP from PDAC, the combination of the 2 markers may help in using steroids more safely in pts with a focal AIP where a diagnosis of pancreatic cancer needs to be excluded. Anti-amylase α-2A may help to diagnose AIP and to differentiate AIP subtypes.
**Buijs (15) 2016, Netherlands**	Anti-PBP	ELISA	AIP type 1 (n = 34), PDAC (n = 29), CP (n =17), PSC (n = 16), 9 H. pylori-negative HC (n = 9) and H.	HISORt ICDC	Elevated serum IgG4 (>1.40 g/l) was present in 81% of AIP pts (n =32). No significant difference in the detection of Ab against the PBP peptide was found among the different patient groups or HC (p =0.69).	No differences were found when comparing the median reactivity in ng/mL of AIP pts (20.1 (IQR 10.0–48.9)) vs. PDAC (17.2 (IQR 5.9–37.2); p =0.27), CP (17.8 (IQR 4.1–38.6); p =0.28), PSC (34.8 (IQR 8.0–69.1); p =0.81), or HC (24.7 (IQR 6.4–88.5); p =0.92). In addition, the sera from H.	There was no significant difference in detecting anti PBP Ab between HC and AIP pts. There was also no difference in the median values of Ab between AIP and PDAC, CP or PSC. There was also no significant difference in median values in relation to PBP status (H. pylori

			pylori –positive HC (n = 9).			pylori-positive controls did not show higher reactivity towards the PBP peptide than H. pylori-negative sera (13.71 (IQR 3.73–145.26) vs. 35.61 (IQR 7.66–63.90), p =0.47).<br>The Sn and Sp of the assay of anti-PBP Ab for differentiating between AIP and PDAC (AUC: 0.58) using a cut-off of 1SD resulted in a Sn of 9% and a Sp of 97%.<br><br>The assay of anti-PBP Ab for differentiating between AIP and other patient groups (AUC: 0.55) using a cut-off of 1SD resulted in a Sn of 9% and a Sp of 93%.	antigen positivity vs. H. pylori negativity). There were no differences in reactivity between AIP pts with or without CST at the moment of sampling. The detection of serum anti-PBP Ab cannot be considered as a potential useful diagnostic tool in AIP.
**Liu (57) 2016, China**	Col IV, Col VII Ab to type IV collagen (anti-Col IV)	ELISA	Type 1 AIP (n =22), PDAC (n = 18), CP pts (n = 14), pts with AIH (n = 14), acute pancreatitis (n = 16), Crohn's (n = 9) and HC (n = 21).	Histology, cytology, serology, imaging	All of the pts with type 1 AIP showed increased serum IgG4 (6.40 g/L; range, 3.23–18.52 g/L). Among the 22 sera with the AIP studied, 12 were positive for anti-Col IV by the immunoblotting method. This was also found in 2 of 18 pts with pancreatic cancer and 3 of 9 pts with Crohn's disease but not by serum specimens from HC and acute pancreatitis.	The concentration of Col IV was significantly higher in AIP pts (199.55±89.10 ng/ mL; p<0.001 pg/mL) compared with the non-collagenous domain, but it was significantly lower than that in the pts with CP. The serum concentration of Col VII was significantly lower in AIP (45.10±7.93 pg/mL) compared with HC (68.53±17.07 pg/mL; p<0.001), AIH (63.71±21.83 pg/mL; p<0.01), and pts with PDAC (63.10 21.07 pg/mL; p<0.001). It was interesting that the concentration of anti-Col IV was also significantly higher in AIP pts (5.49±1.62 μg/mL) compared with CP (4.05±0.69 μg/mL; p<0.05) and PDAC (2.54±0.95 μg/mL; p<0.001), acute pancreatitis (2.42±0.30 μg/mL; p<0.001), AIH (2.59±0.67 μg/mL; p<0.001), and HC (2.80±0.61 μg/mL; p<0.001), but it was not different between Crohn's disease and AIP.	The results of the present study clearly demonstrate high Col IV and anti-Col IV IgG expression in pancreatic tissue from patients with AIP surrounding perivascular fiber, resulting in obliterative vasculitis. In addition, anti-Col IV level elevation may contribute to AIP interstitial collagens deposited. It needs to be determined whether anti-Col IV in AIP is an epiphenomenon due to newly exposed antigens (Col IV) as a consequence of inflammation.

**Detlefsen (21) 2018, Denmark**	Anti-PBP, anti-CA–II, anti-LF, ANA, RF, c–ANCA IgG 4, IgG	ELISA, IIF, nephelometry	AIP (n=29: type 1 AIP (n = 19), type 2 AIP (n=10)), pancreatic cancer (n=17), pancreatic neuroendocrine neoplasm (P–NEN, n=12), and alcoholic CP (n=41).	HISORt	There was a higher number of AIP pts with a low a–PBP than pancreatic cancer pts when using a cutoff of 38.3 U, but the Sn and Sp were very low.<br><br>Elevated serum IgG4 levels (cut-off 1.4g/L) were found in 45% of AIP, 58% of type 1 AIP, and 12% of pancreatic cancer pts. Elevated serum IgG4 levels were seen in all non-AIP patient groups (overall prevalence 7%, range 3–10%), including 9.6% (13/135) pancreatic cancer pts.	There was a lower mean a–PBP value in the AIP group compared with the pancreatic cancer group, but this difference was not significant. There was NS difference in mean a–PBP values in AIP compared with pancreatic cancer. A ROC curve showed that, when using a cut-off of 38.3U, low values of a–PBP had a Sn and Sp of 45% and 71% for differentiating AIP from pancreatic cancer.<br>Median concentration of anti-CA–II was higher in the pancreatic cancer and alcoholic CP groups when compared with AIP, but the difference was not significant.<br><br>Serum IgG4 was significantly higher in AIP pts (mean 4.2 g/L) and especially type 1 AIP pts (mean 6.0 g/L) when compared with pancreatic cancer (mean 0.7 g/L), P–NEN (mean 0.7 g/L) and alcoholic CP (0.9 g/L). The Sn and Sp of IgG4 (cutoff 1.4g/L) for differentiating AIP from pancreatic cancer was 45% and 88%, but rose to 52% and 88% when using a cutoff of 1.09 g/L. When using this cutoff, the Sn and Sp for differentiating type 1 AIP from pancreatic cancer was 68% and 88%. None of the other markers were significantly changed in AIP versus pancreatic cancer. For the differentiation of type 1 and type 2 AIP, the only significant differences were IgG4 in type 1 AIP ($p<0.01$), with a Sn of 68% and a Sp of 80%, and c–ANCA elevations found in some type 2 AIP pts ($p<0.05$).	The only serological marker with a statistically significant difference in mean values between AIP and pancreatic cancer was IgG4. However, the value of IgG4 for the distinction of AIP from pancreatic cancer was limited, probably in part due to the relatively high number of type 2 AIP pts in the study. In accord with recent publications, data did not support a role of increased serum a–PBP for the diagnosis of AIP.

AIP: autoimmune pancreatitis; Sp: specificity; Sn: sensitivity; PPV: positive predictive value; NPV: negative predictive value; anti-LF: antilactoferrin; LF: lactoferrin; CA-II: carbonic anhydrase II; ELISA: enzyme-linked immunosorbent assay; IIF: indirect immune fluorescence; CP: chronic pancreatitis; PDAC: pancreatic ductal adenocarcinoma; HC: healthy controls; SjS: Sjögren's Syndrome; RF: rheumatoid factor; ASMA: anti-smooth muscle antibodies; AMA: anti-mitochondrial antibodies; ANA: anti-nuclear antibodies; anti-PSTI: anti trypsin inhibitor; IgG4: immunoglobulin G4; OR: odds ratio; anti-PBP: anti-plasminogen-binding peptide; DELFIA: dissociation-enhanced lanthanide fluorescence immunoassay; IPMN: intraductal papillary mucinous neoplasm; AUC: area under the curve; ROC: receiver operating characteristics; CI: confidence interval; FT1DM: fulminant type 1 diabetes; T2DM: type 2 diabetes; PRSS1: Serine Protease 1; PRSS2: Serine Protease 2; SPINK1: serine peptidase inhibitor Kazal type 1; NOS-AIP: not-otherwise-specified AIP by ICDC; IgG: immunoglobulin G; SjS: Sjogren's syndrome; PSC: primary sclerosing cholangitis; SD: standard deviation; AIH: autoimmune hepatitis; CA19-9: carbohydrate antigen 19-9; ANCA: anti-neutrophil cytoplasmic antibodies; CST: corticosteroid treatment; NS: not significant.

Supplemental Table S9. Role of Eosinophils and IgE in AIP

Author Year, Country	Candidate biomarker	Diagnostic method	Marker cutoff value	Cohort (n)	Mean (± SD)/Median Sn, Sp, PPV, NPV	Frequency of marker elevation in AIP (%) vs. frequency in controls (%)	Criteria	Differences in marker levels in relation to clinical presentation	Extrapancreatic lesions (EPL)	Other autoimmune/allergic diseases	Conclusion
**Kamisawa (45) 2009, Japan**	IgE, pheripheral Eo count, IgG4	N/A	IgE > 580 IU/mL; Peripheral eosinophilia > 600 cells/mm3.	AIP (n = 45: allergic type (n = 20); nonallergic type (n=25)).	Allergic vs. nonallergic AIP: Serum IgG4 (mg/dl) 298 (198–450) vs. 368 (126–796) p = 0.6. Serum IgE	Elevated serum IgG4 levels were detected in 84% (16/19) of allergic-type AIP pts, and in 82% (18/22) of nonallergic	JPS	Obstructive jaundice as an initial symptom was less frequent in allergic-type AIP pts (p = 0.012). Abdominal pain was	There were no significant differences between the two types in the frequency of associated EPL, such as sclerosing sialadenitis	Twenty pts (allergic-type AIP) had histories of allergic diseases, such as acute allergic rhinitis (n = 11), including rose and hay fever, atopic dermatitis (n = 5), bronchial asthma	Allergic manifestations were detected in about half of the AIP pts, and allergic mechanisms may be related to the occurrence of AIP in these pts. Serum IgE

					(IU/ml) 794 (311–1288) vs. 176 (99–221); p < 0.001.<br><br>Eosinophilia (cells/mm3) 293 (106–582) vs. 160 (104–284); p = 0.048.	-type AIP pts. Serum IgE levels were elevated in 80% (12/15) of allergic-type AIP pts, and in none of 20 nonallergic-type AIP pts (p < 0.0001). Peripheral blood eosinophilia was identified in five (25%) of the allergic-type AIP pts and in none of the nonallergic-type AIP pts (p = 0.013).		detected in only five pts with allergic-type AIP (p = 0.013).	and retroperitoneal fibrosis.	(n = 3), drug allergy (n = 2), and hypersensitivity pneumonitis (n = 1).	levels and the peripheral Eo count were significantly higher in allergic-type AIP patients. There were NS differences between the two types in terms of age at diagnosis, sex ratio, serum IgG4 levels, positivity of auto-Ab, and frequency of associated EPL, such as sclerosing sialadenitis and retroperitoneal fibrosis.
**Hirano (34) 2010, Japan**	IgE	Fluorescence enzyme immunoassay	IgE > 170 IU/mL	AIP (n = 67)<br>*42 sera tested	The average value of IgE was 679 ± 675 IU/mL (range, 67-3000 IU/mL).	IgE was elevated in 86% (36/42) pts. Elevation of IgG and IgG4 were observed in 20 (47%) and 39 (93%) pts, respectivel	HISO Rt JPS	N/A	There was NS difference in IgE in the pts with and without EPL (526 ± 531 IU/mL vs. 819 ± 768 IU/mL, p = 0.163).	Concomitant allergic disease was observed in 7 pts (allergic rhinitis n = 3, bronchial asthma n = 3, and urticaria n = 1). There was NS difference in average IgE values between these seven pts and	There were NS correlations between IgG, IgG4, and IgE. There was NS difference in IgE in the patients with and without EPL, with and without clinical relapse, and

						y. The correlation coefficient of IgG and IgE was -0.168 (NS, p = 0.3). The correlation coefficient of IgG4 and IgE was -0.188 (NS, p = 0.2). The correlation coefficient of IgG and IgG4 was 0.698 (p < 0.0001).				other 35 pts (970 ± 775 IU/mL vs. 621 ± 650 IU/mL, p = 0.2). The frequency of high IgE was 100% (7/7) in these pts and 63% (29/35) in the others; however, this was NS (p = 0.6).	before and after CST. Although IgE does not necessarily reflect the disease activity, IgE might be useful for the diagnosis of AIP in an inactive stage.
**Sah (78) 2010, USA**	IgG4, peripheral Eo count, tissue eosinophilia	The number of Eo per HPF was counted for five fields and average count was taken. Areas with the highest density of eosinophilic infiltrate were evaluated.	Tissue Eosinophilia > 0.5×10^9/L; average peripheral Eo count severe: ≥ 10 moderate: ≥ 5 and < 10.	AIP (n = 97: type 1 (n = 78), type 2 (n = 19)), HC (n = 100)	Median Eo counts in examined resection AIP specimens with peripheral Eo (median count: 5) and without peripheral Eo (median count: 7); p = 0.39.	Among 78 type 1 AIP pts, peripheral eosinophilia at presentation was diagnosed in 12% and allergic disorders in 15% (vs. 0 and 4% in controls, p = 0.0004 and 0.006, respectively). There was NS	HISORt	There was NS association between peripheral eosinophilia and clinical presentation (acute pancreatic, diabetes mellitus, steatorrhea, imaging (focal features/diffuse swelling), IgG4 elevation, EPL).	NS association between either peripheral eosinophilia and proximal bile duct affection only, nor for non-IAC EPL. There was no association between CST and number/onset of peripheral Eo. There was no	Prevalence of allergic disorders did not differ between AIP pts with and without peripheral eosinophilia. Of the AIP pts with allergic disorders, nine had asthma and three had other allergic disorders.	Peripheral eosinophilia and allergic disorders are associated with AIP, with a prevalence of about 28% and 15%, respectively. Pts with and without peripheral eosinophilia were similar in clinical profile. Moderate-to-severe Eo infiltration was present in 67 %

						difference in the number of pts with IgG4 elevation between the group with peripheral Eo and the group without (73.3% (11/15) vs. 81.8% (36/44), p = 0.712). In addition, no correlation was observed between absolute IgG4 counts and peripheral blood Eo counts at presentation (r = 0.22, p = 0.11).			definite responsiveness of eosinophilia to CST. There was no association between eosinophilia at presentation and subsequent relapse.		of pancreas resection specimens and did not correlate with peripheral eosinophilia. Type 2 AIP did not differ from type 1 AIP in any of these parameters.
**van Toorenenbergen (95) 2010, Netherands**	IgE<br>IgG4<br>IgE/IgG4 Ratio	Fluorescence enzyme immunoassay, nephelometry	IgG4 > 1.4 g/L	AIP (n = 13), pancreatic carcinoma (n = 12), atopic	Both total IgE and total IgG4 levels of the 13 pts with AIP were significantly	N/A	HISORt	N/A	N/A	NS difference was observed for total IgE and total IgG4 between the 13 pts with AIP and 14 pts with atopy (p = 0.14 and p = 0.07,	Analysis of total IgE in serum might be useful in the differentiation between AIP and PDAC.

				allergy (n = 14).	higher than those in 12 pts with pancreatic carcinoma (p = 0.0004 and p = 0.015, respectively).<br><br>The diagnostic power of serum IgE and IgG4 for discrimination between AIP and pancreatic carcinoma: IgG4 (1.6 g/L): 69 % Sn and 92% Sp; IgE (136 kU/L): 77% Sn and a 100% Sp; AUC for IgE (0.923) > AUC for IgG4 (0.788), but NS (p = 0.15) probably due to the small sample.					respectively). Total IgE level in the atopic group was significantly higher than that in the pts with pancreatic carcinoma (p < 0.0001); this was not the case for total IgG4 (p = 0.21). The IgE/IgG4 ratio for sera from pts with atopy was significantly different (p = 0.0012) from this ratio in sera from pts with AIP and also different from pts with pancreatic carcinoma (p < 0.0001).	There was a positive correlation between IgG4 and IgE in pts with AIP.
**Kamisawa (50) 2011, Japan**	IgG4, IgG IgE,	Nephelometry	IgG4 > 135 mg/dL (IgG-	AIP (n = 58: IgG4-positive	Mean serum IgG levels were higher	Serum IgG4 levels were	Asian	As an initial symptom, obstructive	EPL, especially sclerosing	NS differences in allergic disease history were	Clinicopathological features of IgG4-negative

	peripheral Eo	positive vs. IgG4-negative sera) Serum IgE > 580 IU/ml Eosinophilia > 600/mm³	group (n = 45), IgG4-negative group (n = 13)).	in the IgG4-positive group (2344.8 ± 966.3 (1220–5580) than in IgG4-negative group 1396.2 ± 277.4 (984–1836). IgG4-positive group vs. IgG4-negative group: Eo (cell/mm³): 283.3 ± 214.6 vs. 354.0 ± 247.6; p = 0.4. Serum IgE (IU/ml): 838.4 ± 1022.8 vs. 1429.1 ± 3266.7; p = 0.2. Auto-Ab: 20/24 (45%) vs. 7/5 (58%); p = 0.552. There were NS differences in the presence of auto-Ab,	elevated in 78% (45/58) of AIP pts (mean, 604.2 ± 526.0 mg/dl).<br><br>IgG4-positive pts vs. IgG4-negative pts: serum IgE (>580 IU/ml): 10/13 (43%) vs. 1/6 (14%); p = 0.2. Peripheral Eo (>600/mm³) : 3/33 (8%) vs. 3/10 (23%); p = 0.3.		jaundice was significantly more frequent in IgG4-positive AIP pts (71%, p = 0.002), and abdominal pain was more frequent in IgG4-negative AIP pts (38%, p = 0.01).	cholecystitis and sclerosing sialadenitis, were frequently detected in IgG4-positive AIP pts (51%, p = 0.008). Acute pancreatitis was more frequent in IgG4-negative AIP pts (23%, p = 0.45). No difference in the frequency of ulcerative colitis, diabetes or PEI between IgG4-positive and IgG4-negative AIP pts.	found between groups.	AIP differed from those of IgG4-positive AIP. There were NS differences in the presence of auto-Ab, serum IgE, and peripheral Eo count.

					serum IgE, or peripheral Eo count.						
**Kuruma (55) 2014, Japan**	IGg4, IgE, peripheral Eo, allergen-specific IgE, Ab serologic assays	Radioallergosorbent test (RAST), multiple-antigen simultaneous test (MAST)	IgG4 < 135 mg/dL IgE < 250 IU/mL; peripheral Eo count < 600 cells/mm3.<br><br>Allergen-specific IgE Ab serologic assays: RAST positive IgE > 0.34 UA/mL MAST positive > 1 class.	AIP type 1(n = 67)	N/A	Peripheral eosinophilia and elevation of serum IgE levels were detected in 16% (10/62) and 60% (32/53) of the pts, respectively. Six (19%) of thirty-one pts with elevated serum IgE level had eosinophilia, while 14% (3/21) pts without an elevated IgE level had eosinophilia (p = 0.9).<br><br>Allergen-specific IgE Ab serologic assays were positive in	ICDC	There were NS differences between the assay-positive and -negative patient groups in obstructive jaundice and acute pancreatitis. There were NS differences between the assay-positive and -negative patient groups in steroid responsiveness and recurrence of AIP.	NS differences between the assay-positive and -negative patient groups in EPL.	Active allergic disease and/or a past history of allergic disease were found in 24 (36%) AIP pts, as follows: hay fever with acute allergic rhinitis, 15 pts (22%); bronchial asthma, 8 pts (12%); drug allergy, 4 pts (6%); atopic dermatitis, 2 pts (3%); and mackerel allergy, house dust allergy and hamster allergy, 1 patient each (1.5%).<br><br>Positive assay vs. negative assay: allergic disease n = 6 vs. n = 1, p = 0.50.	There were NS differences between the assay-positive and -negative patient groups in age, gender, steroid responsiveness, recurrence of AIP, allergic disease, obstructive jaundice, acute pancreatitis, OOI, elevation of serum IgG, IgG4 or IgE levels, or eosinophilia.

						87% (13/15) of pts tested (RAST, 6 of 7 and MAST, 7 of 8).<br><br>Positive assay vs. negative assay: elevation of serum IgG: 7 (54%) vs. 0; elevation of serum IgG4: 9 (69%) vs. 1 (50%); elevation of serum IgE: 8 (62%) vs. 1(50%); eosinophilia: 5 (38%) vs. 0.					
**Yan (98) 2017, China**	Globulin, CA 19-9, Eo, hemoglobin	ECLIA		CA 19-9 > 37 U/ml Eo percentage > 5% Globulin > 35 g/ L	AIP type 1 (n = 25), pancreatic carcinoma (n = 100)	AIP vs. pancreatic carcinoma: CA 19-9 (U/ml): 23.2 (7.6–113.1) vs. 349.8 (24.2–1964.6); Eo%: 5.4 (1.8–6.5) vs. 2.0 (1.3–3.0);	AIP: 44% (11/25) of pts had elevated CA 19-9. Elevated Eo%: 52% (13/25) of pts. Decreased Hb: 76% (19/25) of pts.	ICDC	Jaundice presented more frequently in the AIP group (64%) than the pancreatic carcinoma group (18%), p < 0.001.	N/A	N/A	Elevated serum Eo and globulin levels together with decreased Hb levels can be used as a preoperative indicator for AIP and can help to avoid unnecessary operation.

Hb (g/L): male (M) 114 (105–131) vs. 133 (121–144), female (F) 107 (94–115) vs. 127 (109–132); globulin (g/L): 34.4 (26.1–39.0) vs. 23.1 (25.6–28.3).<br><br>The suggested cut-off value for diagnosis: CA19-9 < 306.75 u/ml, Eo% > 4.15%; globulin > 29.80 g/L and Hb (M) < 114.5 g/L or Hb (F) < 118.5 g/L showed relatively high Sn and Sp (92% and 79%) in AIP diagnosis, with AUC reaching 0.93 ($p < 0.001$).	Elevated globulin: 48% (12/25) of pts.<br><br>Pancreatic carcinoma: 72% (72/100) pts had elevated CA 19-9. Elevated Eo%: 10% (10/100) of pts. Decreased Hb: 40% (40/100) pts. Elevated globulin: 2% (2/100) of pts.

IgE: immunoglobulin E; AIP: autoimmune pancreatitis; Eo: eosinophils; IgG4: immunoglobulin G4; SD: standard deviation; Sp: specificity; Sn: sensitivity; PPV: positive predictive value; NPV: negative predictive value; Eo: eosinophils; N/A: not available; JPS: Japan Pancreas Society; EPL: extrapancreatic lesions; NS: not statistically significant; PDAC: pancreatic ductal adenocarcinoma; AUC: area under the curve; CST: corticosteroid treatment; HPF: high power filed; HC: healthy controls; IAC: IgG4-associated cholangitis; ICDC: international consensus diagnostic criteria; PEI: pancreatic exocrine insufficiency; OOI: other organ involvement; Hb: hemoglobin; CA19-9: carbohydrate antigen 19-9.
